# Influence of Intraoral Scanners, Operators, and Data Processing on Dimensional Accuracy of Dental Casts for Unsupervised Clinical Machine Learning: An In Vitro Comparative Study

**DOI:** 10.1155/2023/7542813

**Published:** 2023-11-22

**Authors:** Taseef Hasan Farook, Saif Ahmed, Jamal Giri, Farah Rashid, Toby Hughes, James Dudley

**Affiliations:** ^1^Adelaide Dental School, The University of Adelaide, Adelaide, Australia; ^2^Department of Electrical and Computer Engineering, North South University, Dhaka, Bangladesh

## Abstract

**Purpose:**

This study assessed the impact of intraoral scanner type, operator, and data augmentation on the dimensional accuracy of in vitro dental cast digital scans. It also evaluated the validation accuracy of an unsupervised machine-learning model trained with these scans.

**Methods:**

Twenty-two dental casts were scanned using two handheld intraoral scanners and one laboratory scanner, resulting in 110 3D cast scans across five independent groups. The scans underwent uniform augmentation and were validated using Hausdorff's distance (HD) and root mean squared error (RMSE), with the laboratory scanner as reference. A 3-factor analysis of variance examined interactions between scanners, operators, and augmentation methods. Scans were divided into training and validation sets and processed through a pretrained 3D visual transformer, and validation accuracy was assessed for each of the five groups.

**Results:**

No significant differences in HD and RMSE were found across handheld scanners and operators. However, significant changes in RMSE were observed between native and augmented scans with no specific interaction between scanner or operator. The 3D visual transformer achieved 96.2% validation accuracy for differentiating upper and lower scans in the augmented dataset. Native scans lacked volumetric depth, preventing their use for deep learning.

**Conclusion:**

Scanner, operator, and processing method did not significantly affect the dimensional accuracy of 3D scans for unsupervised deep learning. However, data augmentation was crucial for processing intraoral scans in deep learning algorithms, introducing structural differences in the 3D scans. *Clinical Significance*. The specific type of intraoral scanner or the operator has no substantial influence on the quality of the generated 3D scans, but controlled data augmentation of the native scans is necessary to obtain reliable results with unsupervised deep learning.

## 1. Introduction

The reliable use of artificial intelligence (AI) in dental research in making accurate diagnostic predictions has been hindered by its inability to perform well on datasets created by different imaging modalities, such as computed tomography, magnetic resonance imaging, and clinical photography across different dental research centers [1–3]. Even with the most reliable operator-driven imaging modalities and 3D scanning tools, inter-rater reliability and agreements are adversely affected by operator-dependent biases [3–5]. As dental research attempts to integrate various forms of 2D datasets, including caries diagnoses from oral photographs and microscopy [6] and endodontic treatment status monitoring from radiographs [7], for AI implementation, it is imperative to determine whether 3D data derived from different clinics and disparate intraoral scanners would impact the dimensional accuracy of the datasets intended to develop AI models.

The types of AI currently applied in medical sciences can be broadly classified into supervised and semisupervised learning [8]. To summarize the fundamental concept of supervised AI in healthcare [9], AI models require clinicians and operators to classify regions of interest in the training dataset of an AI model, which is then applied to test a dataset that the AI model has not seen before [10, 11]. Recent advances in medical AI have introduced the possibility of unsupervised or self-supervised deep learning, which can learn from 3D scans without relying on human-labeled datasets [12]. Historically, human-labeled datasets in dentistry were a source of low reliability and high biases [2]. One such architecture that makes this approach possible is called 3D vision transformer (3DViT), which is a novel deep learning network for processing 3D data. It comprises large transformer layers that perform tasks such as image classification and smaller subunits called attention heads that focus on different parts of the 3D model, providing a 3D reference for image classification [13]. The fundamentals of vision transformer computing have been described in the supplementary materials.

To better understand research data and discover patterns, relationships, and anomalies, exploratory data analysis (EDA) is conducted in data sciences [14]. In this context, the preferred metrics for analysis include validation accuracy, which is determined by the algorithm's correct classification of images from a test dataset labeled by humans, and cross-entropy loss, which quantifies the disparity between predicted class probabilities and true class probabilities. These measurements provide valuable insights into the model's performance and the alignment between predicted and actual outcomes [15]. Yet, to the authors' knowledge, such an approach to quantify scanning performance has not previously been validated on 3D intraoral scans.

The practice of data augmentation is commonplace in medical deep learning as it enriches and diversifies datasets by introducing slight variations [16]. Data augmentation helps in deep learning by artificially expanding the training dataset through various transformations and modifications, which improves model generalization and robustness and reduces overfitting without introducing nonbiological variation that might bias subsequent analysis [17]. Overfitting refers to a scenario in machine learning where a model becomes excessively specialized in training data, performing well on it but failing to generalize to new, unseen data due to learning noise or irrelevant patterns specific to the training set [18]. However, extensive transformation to 3D intraoral scans is discouraged as they can compromise dimensional accuracy, which is essential for prosthetic rehabilitation. Consequently, such modifications have not been previously applied to 3D intraoral scans, as clinical research often relies on the native software provided with the scanners for model processing [19]. Proprietary scanner software themselves have a wide range of preadjusted and locked variables that substantially impact fine-scale 3D vertex details and alignment processes within postprocessing [20]. This limitation hinders further controlled augmentation, which is crucial for achieving predictable machine-learning outcomes that otherwise may not be achievable through native scans alone. In addition, the proprietary nature of most scanning software, often requiring subscription fees, can further prevent controlled augmentation [21]. In order to overcome these limitations, a novel augmentation method was employed in the current study to preprocess completed 3D scan data prior to utilizing AI. This method involved adding layers to the internal surfaces of the natively exported scans, ensuring uniform volumetric depth without modifying the external surface details or texture. One documented approach in the field of medicine to achieve this outcome is through the utilization of an open-source workflow known as “extrusion,” which extends specific regions of the 3D scan along a designated dimension, creating new geometry that is visible to AI models like 3DViT but remains imperceptible to human operators [22]. Notably, this approach has not been previously reported or validated for intraoral scans, making it an innovative contribution to the field of digital dentistry.

The present study aimed to address the lack of published studies exploring biases in the rapidly growing field of dental AI caused by using different intraoral scanning (IOS) to generate scans for data augmentation and deep learning. To achieve this, an unsupervised 3DViT model was implemented where EDA was repurposed to quantify the performance of 3D intraoral scans. The datasets were also subjected to controlled augmentation to explore the effectiveness of the proposed technique.

The objective of this study was to evaluate the dimensional accuracy of in vitro cast model scans acquired by various operators using two distinct handheld intraoral scanners, with a benchtop laboratory scanner being treated as the criterion standard. Additionally, the study aimed to assess the suitability of the data for unsupervised machine learning after employing a customized augmentation procedure. It was hypothesized that there would be no significant differences or interactions among different operators, scanners, and augmentation methods as independent variables impacting the dataset created for deep learning purposes.

## 2. Materials and Methods

**Figure 1:**
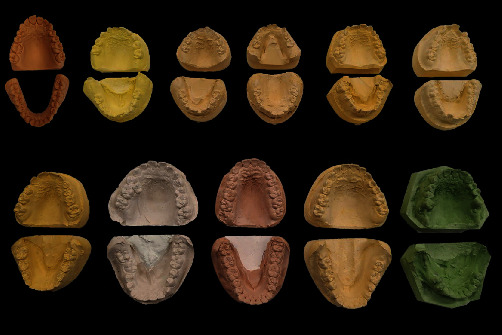
Physical cast model dataset.

The present study utilized a diverse dataset comprising one set of 3D-printed casts and 10 sets of deidentified gypsum casts obtained from the University of Adelaide's repository. The gypsum casts introduced conventional variations in arch morphology, while the 3D-printed casts introduced modern variations to materials for cast manufacturing [23]. In order to minimize variations caused by salivary reflections and manual dexterity during cheek retraction techniques, in vivo tests of intraoral scans were replaced with the use of multicolored casts in vitro [24, 25]. The study was deemed exempt from ethical review as no identifiable data were used, and no human participants were recruited for the task. Each set of casts included upper and lower arches, resulting in 22 distinct cast models. The casts belonged to individuals aged between 6 and 33 years, encompassing primary, mixed, and permanent dentitions. The randomly assigned dataset exhibited variations in tooth alignment, ethnicity (as determined from the repository), abnormalities in tooth and palatal development, and dental trauma and were constructed using gypsum material of varying colors (Figure 1).

Data collection involved the use of two handheld scanners, IOS1 (TRIOS 3; 3Shape) and IOS2 (AoralScan 3; Shining3D), operated by two specified dental operators (OP1 and OP2). To account for operator-dependent biases, a laboratory desktop 3D scanner (E4; 3Shape) was also incorporated into the workflow alongside the handheld scanners to facilitate a control standard. This was rationalized by the laboratory scanner not requiring constant manual inputs to generate data and, therefore, was likely free from potential operator biases. The current study conducted a power analysis based on an effect size (*F* = 0.40) [26, 27] with *α* = 0.05 and 1 – *β* = 0.80, recommending a sample size of 80 cast models distributed among five groups (*n* = 16) with each operator scanned the 22 models with both scanners. The scanning groups were classified as Lab scanner, IOS1 + OP1, IOS1 + OP2, IOS2 + OP1, and IOS2 + OP2, and yielded a total sample size of 110 virtual models that were classified as “native scans” as they were not augmented in any capacity.

The scanning methods were adapted from previously published reports to ensure some degree of homogeneity across operators [28]. The IOS method involved scanning the occlusal surfaces first, followed by facio-buccal and then the lingual. Secondary scans were obtained by angulating the scanning lens mesially to account for tooth surface metamerism and capture reflective surfaces that might create voids during perpendicular scanning techniques [28, 29]. It is worth noting that all scanners were calibrated according to the manufacturers' recommendations prior to the commencement of data collection. All scanned models were exported as standard tessellation language (.stl) files. Comparisons were made for Hausdorff's distance (HD) [30] and root mean squared error (RMSE) [31] by considering the dataset derived from the benchtop scanner as standard. HD is employed to assess the maximum deviation between two surfaces, such as dental models, encompassing both overestimation and underestimation. In contrast, RMSE calculates the average disparity between corresponding points of the two surfaces, highlighting the overall accuracy of the scan without distinguishing between overestimation and underestimation [30, 31].

**Figure 2:**
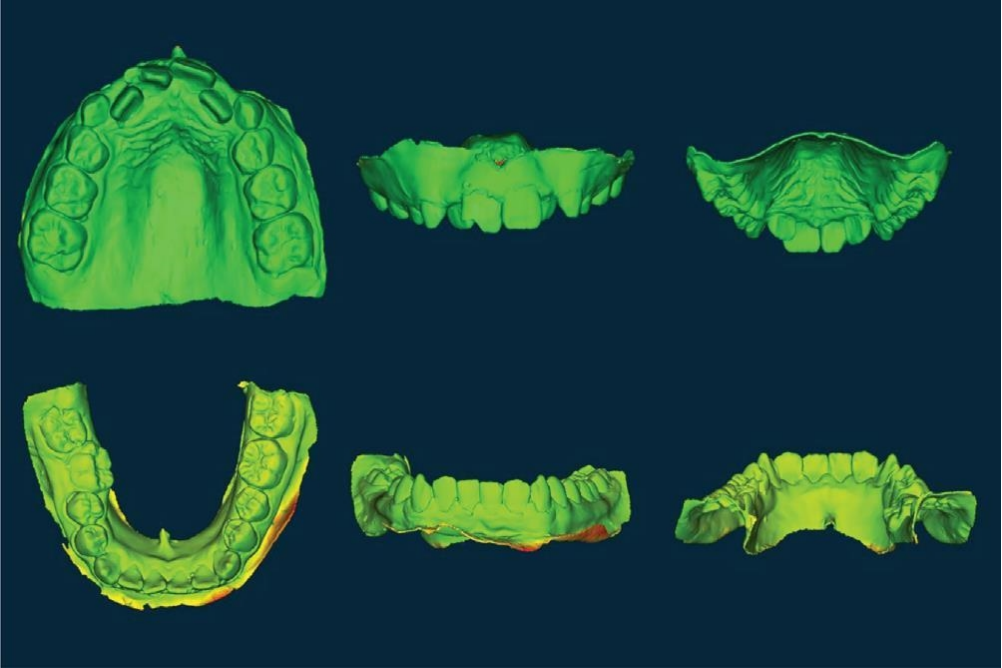
Heatmaps generated following Hausdorff's distance and RMSE evaluations (individual heatmaps and histograms presented within the supplementary file).

**Figure 3:**
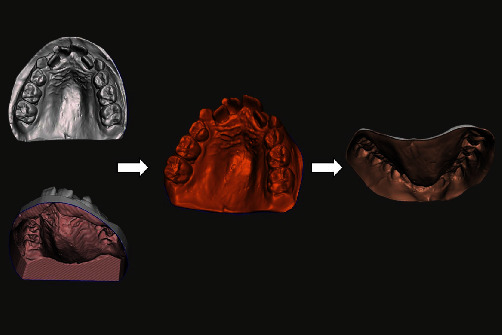
The augmentation process and the list of commands that provided uniform extrusion in Meshmixer: “select” all > “extrude −0.5 mm” > “extrude along normal axis.”.

To test the method of data augmentation, a new dataset was generated by segmenting the dental arch from the base and extruding the native scan files to a 0.5 mm inner thickness against the “normal” axis in CAD (Meshmixer; Autodesk Inc.). (Figure 2) The new 3D models formed the “augmented dataset,” whose resultant shape and form were overlapped and compared once against their native scan counterparts and once against the augmented dataset of the laboratory scanner using HD and RMSE. (Figure 3) A Kruskal–Wallis 1-way nonparametric test was performed to compare HD differences between the native scans and their corresponding augmented scans.

In the current study, it is important to note that the use of HD and RMSE was intended to offer a relative context regarding the influence of the factors examined. They were employed in processing the outcomes through a 3-factor analysis of variance, serving as a means of comparison rather than an absolute measure of dimensional accuracy. The distribution of the residuals was evaluated and deemed to be normally distributed with a *z*-score = 2.131 within the current sample size of 110.

**Figure 4:**
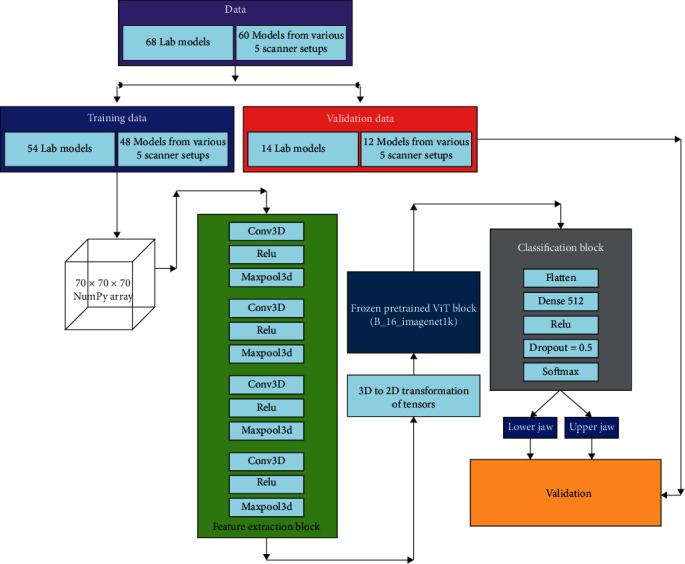
Architecture of the neural network classifier, its components, and class prediction process.

The scanned models were introduced to the 3DViT that trained itself to differentiate features between upper and lower jaws. As the native scans could not be used for training the 3DViT model, 68 additional synthetic jaw models were randomly scanned using the intraoral scanners and augmented to assist in the training process. All cast scans were randomly allocated into train and test sets, where the test sets were completely unseen to the AI model during the initial training. As shown in Figure 4, the neural network model comprised a feature selection block with four layers of 3D convolution, a rectified linear unit (ReLU), and Maxpool3D filters, which were responsible for converting 3D data into 2D tensors. These 2D tensors were then fed into a ViT block pretrained on the ImageNet dataset, which includes 1,000 object classes and a total of 1,281,167 training images, 50,000 validation images, and 100,000 test images. The latent vectors produced by this block were subsequently directed to a classification block, which involved flattening the output vectors, transferring them to a dense layer, followed by a ReLU activation, connected to a dropout layer, and finally passed through a SoftMax layer for the final binary classification.

The model's training process was guided by accuracy and binary cross-entropy loss since the dataset consisted of only two classes. While the parameters of the feature selector and the classification block were trained during the process, the ViT block was left frozen. A validation accuracy test was performed on 50 unseen scanned models (10 from each group: LAS, IOS1 + OP1, IOS1 + OP2, IOS2 + OP1, and IOS2 + OP2).

The overall process followed a single-blinded approach where the data sources were anonymized prior to machine-learning modeling to mitigate any cognitive or prejudicial biases [32]. Errors in scanning technology were concurrently estimated while undertaking RMSE and HD evaluations. The training parameters were as follows: epochs = 50, batch size = 2, learning rate = 0.00001, a weight decay of 0.0000000001, and momentum of 0.999. The test accuracy of the model obtained after two epochs was 96.2%, and the loss was 0.0806, which caused the training procedure to have an early stopping.

## 3. Results

Table 1 shows that there were no significant differences or interactions observed in the evaluation of HD across all three variables. The dimensional differences between the two scanners ranged from 0.16 ± 0.22 to 0.21 ± 0.48 mm when compared to the lab scanner. Similarly, the differences between the two clinical operators were small, ranging from 0.17 ± 0.34 to 0.19 ± 0.41 mm. Data for each individual cast have been provided within supplementary materials (Tables S1 and S2). Across the four groups of handheld scanners, there were no significant differences (*H* = 4.243, *p*=.236) observed when comparing the outcomes of augmentation for cast models to their corresponding scan models (Table S3). There were, however, significant differences in RMSE between native and augmented model datasets across both operators and scanners. Regardless, there were no significant interactions between the type of scanner used, operator-dependent variables, or the application of data augmentation. (Table 2) The lack of a significant interaction effect across the varying combinations of variables suggests that the current dataset remained unaffected by the variations produced by the difference in scanner technology and operators' capabilities. Metadata contributing to the outcomes described have been documented within supplementary materials (Table S4 and S5).

The deep-learning network was unable to train or predict outcomes with the native scans owing to a lack of volumetric depth when processed through the numerical Python programing (NumPy) array. Native images captured by intraoral scanners, although appear 3D, were seen to lack depth along the *z*-axis that was obtained in the current study following extrusion. As was observed with the native scans, the lack of volume resulted in only surface geometry being registered and, therefore, were not treated as 3D models by the NumPy array during 3DViT implementation. The process flowchart has been described in Figure 4. The 3DViT model achieved a validation accuracy of 96.2% across individual group (LAS, IOS1 + OP1, IOS1 + OP2, IOS2 + OP1, and IOS2 + OP2) performances when tested the augmented dataset. A detailed breakdown of the validation outputs for cast models has been mentioned below and detailed in the supplementary materials (Table S6).

- Lab scanner (standard): All 10 models were accurately classified by the model.
- IOS1 + OP1: All 10 models were accurately classified by the model.
- IOS1 + OP2: 9 out of the 10 models were accurately classified by the model.
- IOS2 + OP1: 8 out of the 10 models were accurately classified by the model.
- IOS2 + OP2: 9 out of the 10 models tested were accurately classified by the model.

## 4. Discussion

**Figure 5:**
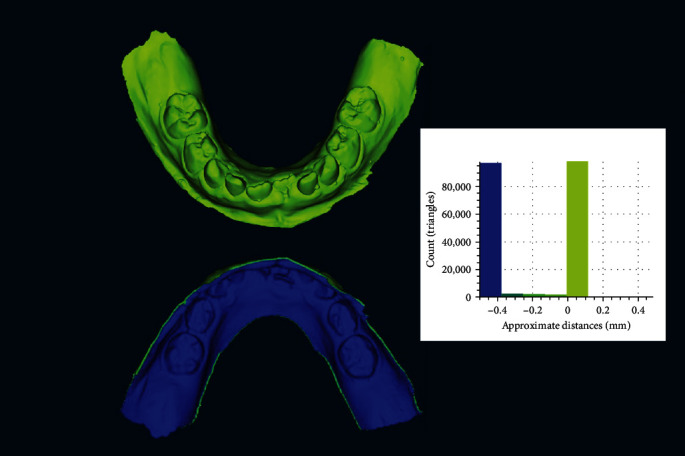
A qualitative visualization to demonstrate the volumetric characteristics of the augmented scans. The blue regions were produced from the augmentation process, providing a 0.5 mm inner thickness to the 3D casts without influencing any of the clinical features (green regions) superficially.

This study assessed the impact of variables, including scanners, operators, and data augmentation, on the dimensional accuracy of in vitro cast model scans. Additionally, it evaluated the performance of an unsupervised machine-learning model trained with these scans. The results demonstrated significant differences in RMSE between the native and augmented datasets, rejecting the initial hypothesis. However, no significant differences or interactions were found in the HD evaluations across the three independent variables. These findings indicate that scanner and operator choices had no significant influence on dimensional accuracy, while the augmentation procedure significantly altered the internal structure of the 3D scans (Figure 5), enabling their use in unsupervised deep learning with visual transformers.

The scanners were in separate facilities with distinct environmental and temperature controls and were operated by two individuals, thereby simulating a multicentre approach [1, 33]. Test validations were conducted in groups representing different scanners and operators that may be encountered in various clinics. Validation accuracy is an important metric to assess how well the model is performing and to make decisions about model architecture, hyperparameter tuning, and potential overfitting. In the context of prosthetic dentistry, overfitting would result in poor performance when making predictions on different dental casts, as the model has become too specialized and memorized the training data, leading to higher error rates on newly introduced 3D intraoral scans [34]. A high validation accuracy in the current model, however, suggests that the model is learning the patterns and features in the data effectively and is likely to perform well on unseen patient data.

The study encompassed a wide range of scenarios encountered in a clinical research facility by incorporating variations in the environment and the cast models, including differences in age, ethnicity, and dentition status. It is important to note that in real-world scenarios affected by ambient light, the resolution of full arch oral scans is typically lower compared to single tooth or single quadrant scans, leading to potential deviations from the advertised accuracy observed in controlled laboratory environments as was seen in the current experiment [35, 36]. The primary discrepancies were observed in the buccal, labial, and lingual sulci, with additional variations noticed on the palatal aspect of a cast from a patient with a deep palate, particularly in the palatal rugae. The heatmaps and histograms provided in the supplementary materials illustrate these discrepancies. Considering that HD and RMSE measurements were not significant, it is likely that these discrepancies were caused by the scanning technique and limitations set by the operators, rather than the scanners themselves, implying cognitive biases at play. However, it is important to note that these discrepancies did not have a significant impact on the dimensional accuracy of the scans or the subsequent development of deep learning models.

The 3D scanning industry has been dominated by a small number of industry leaders who have imposed premium yearly subscriptions for their postprocessing tools. However, the current market is witnessing the emergence of affordable intraoral scanners without subscription requirements, offering native software suites [19]. While minimal differences were observed among the scanners used in the study, it is essential to note that both scanners adequately performed the basic scanning operation in their designated facilities. To ensure controlled augmentation, third-party modification tools must be utilized. This raises the question of whether it is necessary to continue investing in maintaining industry standards or instead focus on integrating and fine-tuning newer, more cost-effective scanning methods into research-academia protocols.

The scans underwent a uniform normalization technique without any postprocessing features, utilizing a 0.5 mm inner-thickness extrusion. This approach aimed to replicate the appropriate layer thickness observed in medical bioprinting of knee models [37]. Nevertheless, the ideal thickness for 3D-printed cast models remains unexplored and warrants future investigation as a potential recommendation.

The native model-building function provided by the scanner's software suite was not utilized to ensure that equal volume was added across all scan media. Interestingly, prior to this augmentation, the 3DViT model was unable to render the 3D models in their original format due to the scan's lack of depth and solid dimension. It is also important to note, however, that prior to the study, the 3DViT model had been developed and trained on 60 synthetic dental arches, ensuring its suitability for handling full oral arches. This highlights the essential role of software-based postprocessing and consistent scanning outcomes in clinical deep learning. However, the subscription model imposed by manufacturers, coupled with researchers' limited funds, may hinder the progress of such advancements. Additionally, obtaining timely support for proprietary software and devices can be time-consuming and costly [38]. The inability of the 3DViT model to render directly exported 3D models in their native formats underscores the necessity of the augmentation method, particularly for researchers who lack subscriptions to the scanner manufacturers' software add-ons. Consequently, the current study faced limitations in validating outcomes using the native dataset as the numerical processes involved with the 3DViT model were unable to compute the third dimension of the native scans directly exported without any native postprocessing.

Lower accuracies were observed in the IOS1 + OP2 and IOS2 + OP1 groups. Further analysis revealed that the misclassified casts exhibited no discernible pattern across the different age groups and dentition status, except that three out of the four misclassifications were associated with lower arches. This suggests that displacement of tongue position during impression-taking, which then manifests in the poured gypsum casts, could introduce minute discrepancies not typically encountered when directly scanning the patient's jaw [25, 39]. It can, however, be argued that placement of impression trays, disparate to IOS, is technique sensitive, and uniform application of pressure could possibly yield even displacement of the buccal mucosa and result in similarly effective outcomes [40]. The lack of a significant interaction among the three variables implies the absence of a multifactorial explanation for the misclassifications, indicating that distinct anatomical variations or a relatively small dataset could be contributing factors [25]. While the current EDA sheds light on the impact of the responsible variables, it fails to elucidate the underlying cause of the seemingly randomized misclassifications. It is entirely possible that variations between operator and scanner combinations in terms of physical access to anatomical landmarks of the lower jaw and the capture of relevant data points could potentially affect differentiation. Future studies could employ an explainable AI model that is capable of providing transparent and interpretable explanations for its predictions or decision-making process [41] to gain deeper insights into the degree of impact specific 3D jaw landmarks contribute to misclassifications in the AI model [42].

Scanner calibration can be challenging due to the generation of heat during real-time scanning, making it susceptible to temperature fluctuations and could have contributed to some of the variations experienced in the current study [33]. In regions with diverse weather conditions, recalibration may be required more frequently unless located in a controlled laboratory environment, which would increase the overhead cost. IOS1 demonstrated validation accuracies comparable to laboratory scanners, while IOS2 exhibited significantly faster scanning speed compared to IOS1. Although this did not impact on the current study's cast model dataset, it could introduce operator biases in an in vivo setting due to subjective factors such as scanner weight and speed. The overall scanning accuracy often remained mostly unaffected by scanner versions or generations, with additional features mainly contributing to user convenience [19, 43]. Moreover, since each scanning system uses different tools for processing models, attempts to unify the augmentation process using a single set of tools resulted in varying changes in RMSE when compared to the native scan files. This is because the algorithm that converts the .stl files into layers of images and concatenates them to form 3D arrays that interpret the data from each scanner differently, as the scanners have different algorithms of mesh generation [44].

The current research design has certain limitations, as it did not account for variables such as temperature, ambient light control, or cast material characteristics. Additionally, the impact of relocating the scanners to a new facility on dimensional accuracy was not explored, which could be considered as a limitation and a recommendation for future investigation. To address these limitations, it is recommended that future studies incorporate in vivo scans from diverse geographic locations and consider factors like medical and dental history, as well as supplementary radiographic data, during the training of 3DViTs. This approach would leverage unsupervised learning to gather valuable information and facilitate informed diagnoses of patient-specific occlusal features.

## 5. Conclusion

The choice of scanner, operator, and processing method did not have a significant impact on the dimensional accuracy of 3D dental cast scans for unsupervised deep learning. However, data augmentation played a crucial role in processing intraoral scans, introducing structural differences in the 3D scans.

## Figures and Tables

**Table 1:** 3-Factor ANOVA to evaluate the relationship between scanners, operators, and processing workflow and subsequent Hausdorff's distance (mm).

List of independent factors			
3D scanner	*F* = 0.94, *p*=.33	IOS1 = mean ± SD = 0.16 ± 0.22	
		IOS2 = mean ± SD = 0.21 ± 0.48	
Augmentation	*F* = 0.61, *p*=.44	Native dataset = mean ± SD = 0.21 ± 0.47	
		Augmented dataset = mean ± SD = 0.16 ± 0.25	
Clinical operator	*F* = 0.14, *p*=.71	Operator 1 = mean ± SD = 0.19 ± 0.41	
		Operator 2 = mean ± SD = 0.17 ± 0.34	

Native dataset			

	Operator 1 (mean ± SD)		Operator 2 (mean ± SD)
IOS1	0.14 ± 0.21		0.18 ± 0.24
IOS2	0.28 ± 0.71		0.22 ± 0.52

Augmented dataset			

	Operator 1 (mean ± SD)		Operator 2 (mean ± SD)
IOS1	0.17 ± 0.22		0.14 ± 0.23
IOS2	0.19 ± 0.28		0.15 ± 0.28

Interaction effect			

(1) 1. 3D scanner vs. augmentation: *F* = 0.45, *p*=.50 (2) 2. 3D scanner vs. clinical operator: *F* = 0.21, *p*=.65 (3) 3. Augmentation vs. clinical operator: *F* = 0.03, *p*=.86 (4) 4. 3D scanner vs. augmentation vs. clinical operator: *F* = 0.18, *p*=.67			

^*∗*^Significant *p* < .05.

**Table 2:** 3-Factor ANOVA to evaluate the relationship between scanners, operators, and processing workflow and subsequent RMSE outcomes.

List of independent factors			
3D scanner	*F* = 2.23, *p*=.14	IOS1 = mean ± SD = 34.25 ± 5.89	
		IOS2 = mean ± SD = 33.02 ± 4.93	
Augmentation	*F* = 4.90, *p*=.028^*∗*^	Native dataset = mean ± SD = 34.54 ± 5.80	
		Augmented dataset = mean ± SD = 32.73 ± 4.94	
Clinical operator	*F* = 0.24, *p*=.62	Operator 1 = mean ± SD = 33.43 ± 5.63	
		Operator 2 = mean ± SD = 33.83 ± 5.30	

Native dataset			

	Operator 1 (mean ± SD)		Operator 2 (mean ± SD)
IOS1	35.01 ± 7.21		35.18 ± 5.66
IOS2	33.63 ± 5.21		34.35 ± 5.17

Augmented dataset			

	Operator 1 (mean ± SD)		Operator 2 (mean ± SD)
IOS1	33.10 ± 5.66		33.70 ± 4.95
IOS2	31.99 ± 3.89		32.12 ± 5.25

Interaction effect			

(1) 1. 3D scanner vs. augmentation: *F* = .021, *p*=.88 (2) 2. 3D scanner vs. clinical operator: *F* <.001, *p*=.98 (3) 3. Augmentation vs. clinical operator: *F* = .003, *p*=.96 (4) 4. 3D scanner vs. augmentation vs. clinical operator: *F* = .097, *p*=.76			

^*∗*^Significant *p* < .05.

## Data Availability

The link to access the codes and scripts utilized for running the model can be found at https://github.com/aguynamedSaif/EDA_various_jaw_scanner_and_jaw_type_classifier (accessed on October 31, 2023). Additionally, all the data utilized in reaching the conclusions have been provided as a unified supplementary file.

## Supplementary Materials

Supplementary Materials A single supplementary file has been provided, which describes the fundamentals of transformer-based AI technology and contains tabulated metadata used in the current study to reach the conclusions described. Tables S1–S5: consisting of data generated for evaluations of Hausdorff's distance and root mean square error across both native and augmented scans. Table S6: outlines the validation accuracies obtained for each individual cast model following deep learning using 3DViT. For each comparison made, individual heatmaps of native and augmented scans have also been generated and are included within the same supplementary file.

## References

[1] Nozawa M., Ito H., Ariji Y. (2022). Automatic segmentation of the temporomandibular joint disc on magnetic resonance images using a deep learning technique. *Dentomaxillofacial Radiology*.

[2] Rousseau M., Retrouvey J.-M. (2022). Machine learning in orthodontics: automated facial analysis of vertical dimension for increased precision and efficiency. *American Journal of Orthodontics and Dentofacial Orthopedics*.

[3] Farook T. H., Dudley J. (2023). Automation and deep (machine) learning in temporomandibular joint disorder radiomics: a systematic review. *Journal of Oral Rehabilitation*.

[4] Farook T. H., Rashid F., Ahmed S., Dudley J. (2023). Clinical machine learning in parafunctional and altered functional occlusion: a systematic review. *The Journal of Prosthetic Dentistry*.

[5] Hung K., Yeung A. W. K., Tanaka R., Bornstein M. M. (2020). Current applications, opportunities, and limitations of AI for 3D imaging in dental research and practice. *International Journal of Environmental Research and Public Health*.

[6] Khanagar S. B., Alfouzan K., Awawdeh M., Alkadi L., Albalawi F., Alfadley A. (2022). Application and performance of artificial intelligence technology in detection, diagnosis and prediction of dental caries (DC)—a systematic review. *Diagnostics*.

[7] Hasan H. A., Saad F. H., Ahmed S., Mohammed N., Farook T. H., Dudley J. (2023). Experimental validation of computer-vision methods for the successful detection of endodontic treatment obturation and progression from noisy radiographs. *Oral Radiology*.

[8] Leite A. F., de Vasconcelos K. F., Willems H., Jacobs R. (2020). Radiomics and machine learning in oral healthcare. *Proteomics–Clinical Applications*.

[9] Meskó B., Görög M. (2020). A short guide for medical professionals in the era of artificial intelligence. *npj Digital Medicine*.

[10] Salahin S. M. S., Ullaa M. D. S., Ahmed S., Mohammed N., Farook T. H., Dudley J. (2023). One-stage methods of computer vision object detection to classify carious lesions from smartphone imaging. *Oral*.

[11] Tareq A., Faisal M. I., Islam M. S. (2023). Visual diagnostics of dental caries through deep learning of non-standardised photographs using a hybrid YOLO ensemble and transfer learning model. *International Journal of Environmental Research and Public Health*.

[12] Benčević M., Habijan M., Galić I., Pizurica A. (2022). Self-supervised learning as a means to reduce the need for labeled data in medical image analysis.

[13] Lin K., Wang L., Liu Z. End-to-end human pose and mesh reconstruction with transformers.

[14] Milo T., Somech A. Automating exploratory data analysis via machine learning: an overview.

[15] Park E., Ahn J., Yoo S. Weighted-entropy-based quantization for deep neural networks.

[16] Chlap P., Min H., Vandenberg N., Dowling J., Holloway L., Haworth A. (2021). A review of medical image data augmentation techniques for deep learning applications. *Journal of Medical Imaging and Radiation Oncology*.

[17] Cui X., Goel V., Kingsbury B. (2015). Data augmentation for deep neural network acoustic modeling. *IEEE/ACM Transactions on Audio, Speech, and Language Processing*.

[18] Dietterich T. (1995). Overfitting and undercomputing in machine learning. *ACM Computing Surveys*.

[19] Amornvit P., Rokaya D., Sanohkan S. (2021). Comparison of accuracy of current ten intraoral scanners. *BioMed Research International*.

[20] Vlasic D., Baran I., Matusik W., Popović J. (2008). Articulated mesh animation from multi-view silhouettes. *ACM Transactions on Graphics*.

[21] Boulanger A. (2005). Open-source versus proprietary software: is one more reliable and secure than the other?. *IBM Systems Journal*.

[22] Kurenov S. N., Ionita C., Sammons D., Demmy T. L. (2015). Three-dimensional printing to facilitate anatomic study, device development, simulation, and planning in thoracic surgery. *The Journal of Thoracic and Cardiovascular Surgery*.

[23] Park J.-M., Jeon J., Koak J.-Y., Kim S.-K., Heo S.-J. (2021). Dimensional accuracy and surface characteristics of 3D-printed dental casts. *The Journal of Prosthetic Dentistry*.

[24] Patzelt S. B. M., Emmanouilidi A., Stampf S., Strub J. R., Att W. (2014). Accuracy of full-arch scans using intraoral scanners. *Clinical Oral Investigations*.

[25] Parize H., Tardelli J. D. C., Bohner L., Sesma N., Muglia V. A., dos Reis A. C. (2022). Digital versus conventional workflow for the fabrication of physical casts for fixed prosthodontics: a systematic review of accuracy. *The Journal of Prosthetic Dentistry*.

[26] Faul F., Erdfelder E., Lang A.-G., Buchner A. (2007). G^*∗*^ Power 3: a flexible statistical power analysis program for the social, behavioral, and biomedical sciences. *Behavior Research Methods*.

[27] Farook T. H., Rashid F., Jamayet N. B., Abdullah J. Y., Dudley J., Khursheed Alam M. (2022). A virtual analysis of the precision and accuracy of 3-dimensional ear casts generated from smartphone camera images. *The Journal of Prosthetic Dentistry*.

[28] Richert R., Goujat A., Venet L. (2017). Intraoral scanner technologies: a review to make a successful impression. *Journal of Healthcare Engineering*.

[29] Rashid F., Farook T. H., Dudley J. (2023). Digital shade matching in dentistry: a systematic review. *Dentistry Journal*.

[30] Farook T. H., Abdullah J. Y., Jamayet N. B., Alam M. K. (2022). Percentage of mesh reduction appropriate for designing digital obturator prostheses on personal computers. *The Journal of Prosthetic Dentistry*.

[31] El Barhoumi N., Hajji R., Bouali Z., Ben Brahim Y., Kharroubi A. (2022). Assessment of 3D models placement methods in augmented reality. *Applied Sciences*.

[32] Lum K. (2017). Limitations of mitigating judicial bias with machine learning. *Nature Human Behaviour*.

[33] Revilla-León M., Gohil A., Barmak A. B. (2023). Influence of ambient temperature changes on intraoral scanning accuracy. *The Journal of Prosthetic Dentistry*.

[34] Cox G. M., Gibbons J. M., Wood A. T. A., Craigon J., Ramsden S. J., Crout N. M. J. (2006). Towards the systematic simplification of mechanistic models. *Ecological Modelling*.

[35] Lepidi L., Galli M., Mastrangelo F. (2021). Virtual articulators and virtual mounting procedures: where do we stand?. *Journal of Prosthodontics*.

[36] Rashid F., Jamayet N. B., Farook T. H. (2022). Color variations during digital imaging of facial prostheses subjected to unfiltered ambient light and image calibration techniques within dental clinics: an in vitro analysis. *PLoS ONE*.

[37] Zhang M., Lei M., Zhang J. (2022). Feasibility study of three-dimensional printing knee model using the ultra-low-dose CT scan for preoperative planning and simulated surgery. *Insights into Imaging*.

[38] Farook T. H., Barman A., Abdullah J. Y., Jamayet N. B. (2021). Optimization of prosthodontic computer-aided designed models: a virtual evaluation of mesh quality reduction using open source software. *Journal of Prosthodontics*.

[39] Nejatian T., Firouzmanesh P., Syed A. U. Y. (2019). Dental gypsum and investments. *Advanced Dental Biomaterials*.

[40] Thomas C. J. (1990). Impression material consistency and peripheral tissues. *Australian Dental Journal*.

[41] Shin D. (2021). The effects of explainability and causability on perception, trust, and acceptance: implications for explainable AI. *International Journal of Human-Computer Studies*.

[42] Xu F., Uszkoreit H., Du Y., Fan W., Zhao D., Zhu J. Explainable AI: a brief survey on history, research areas, approaches and challenges.

[43] Kihara H., Hatakeyama W., Komine F. (2020). Accuracy and practicality of intraoral scanner in dentistry: a literature review. *Journal of Prosthodontic Research*.

[44] Erozan Ç., Ozan O. (2020). Evaluation of the precision of different intraoral scanner-computer aided design (CAD) software combinations in digital dentistry. *Medical Science Monitor*.

