# Supplementary material for: Influence of Intraoral Scanners, Operators, and Data Processing on Dimensional Accuracy of Dental Casts for Unsupervised Clinical Machine Learning: An In Vitro Comparative Study
Source: Int J Dent. 2023 Nov 22;2023:7542813. doi: 10.1155/2023/7542813 (PMC10686707; doi:10.1155/2023/7542813)
Supplement: Supplementary Materials — A single supplementary file has been provided, which describes the fundamentals of transformer-based AI technology and contains tabulated metadata used in the current study to reach the conclusions described. Tables S1–S5: consisting of data generated for evaluations of Hausdorff's distance and root mean square error across both native and augmented scans. Table S6: outlines the validation accuracies obtained for each individual cast model following deep learning using 3DViT. For each comparison made, individual heatmaps of native and augmented scans have also been generated and are included within the same supplementary file. [file 7542813.f1.pdf]

Influence of intraoral scanners, operators, and data processing on dimensional accuracy of dental casts for unsupervised clinical machine learning: An invitro comparative study

Supplementary File

Contents

The fundamentals of vision transformer computing 2

Table S1. Hausdorff's Distance Data table of native scans (Lab scanner considered standard) 3

Table S2. Hausdorff's Distance Data table of augmented scans (Lab scanner considered standard) 4

Table S3. Hausdorff's Distance Data table comparing native dataset vs augmented dataset 5

Table S4. Root mean square error (RMSE) value of native scans (Lab scanner considered standard) 6

Table S5. Root mean square error (RMSE) value of augmented scans (Lab scanner considered standard) 7

Heatmaps of Native scans generated through Hausdorff's distance evaluation 8

IOS1 + OP1 8

IOS1 + OP2 16

IOS2 + OP1 24

IOS2 + OP2 32

Heatmaps of Augmented scans generated through Hausdorff's distance evaluation 40

IOS1 + OP1 40

IOS1 + OP2 48

IOS2 + OP1 56

IOS2 + OP2 64

Table S6. Validation Accuracy outcomes from 3DViT 72

Additional References 73

The fundamentals of vision transformer computing

Vision Transformers[i] break an image into patches, which are flattened and linearly projected to obtain their representations. These representations are then processed by a transformer encoder, a powerful neural network architecture for sequence modeling. The transformer encoder[ii] captures spatial relationships and dependencies between patches, enabling the model to understand the image's content. Finally, a multilayer perceptron[iii] (MLP) head is used to make predictions based on the encoded information.

The linear projection of flattened patches is obtained by multiplying the patch matrix with a learnable weight matrix. This projection transforms the pixel-level information into a higher-dimensional representation, enhancing the model's ability to capture complex patterns.

The transformer encoder consists of multiple layers, each composed of self-attention mechanisms[iv] and feed-forward neural networks. Self-attention allows the model to weigh the importance of different patches based on their relationships, while the feed-forward networks introduce non-linear transformations to enhance feature representation[v].

The MLP head takes the encoded features and performs additional transformations and computations to produce the final predictions. It typically includes multiple layers with nonlinear activation functions to learn complex decision boundaries[vi].

Patch + Position embedding[vii] is used to provide spatial information[viii] to the model. It combines the patch embeddings with positional encodings, which encode the spatial location of each patch. This enables the model to understand the arrangement and context of patches within the image, improving its ability to recognize objects and understand the image's structure.

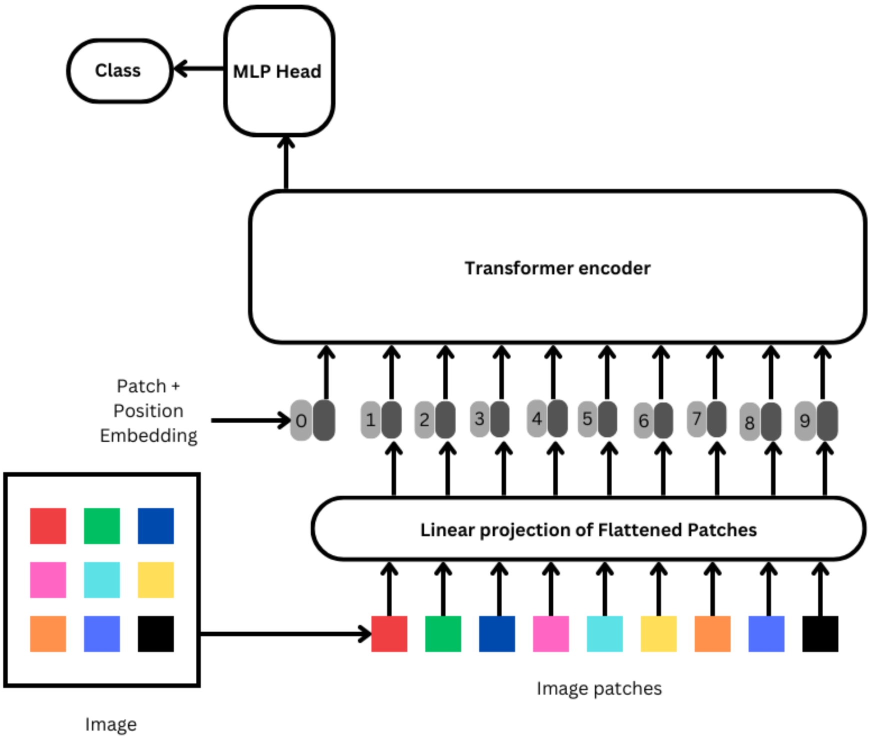

Table S1. Hausdorff's Distance Data table of native scans (Lab scanner considered standard)

Sample	IOS1 + OP1		IOS1 + OP2		IOS2 + OP1		IOS2 + OP2	
	Mean	*SD*	*Mean*	*SD*	*Mean*	*SD*	*Mean*	*SD*
3D printed lower	0.67	2.00	0.77	1.33	2.01	2.66	2.28	2.27
3D printed upper	0.52	1.22	0.47	1.11	2.78	0.72	0.68	1.41
01 Lower	0.06	0.29	0.25	0.59	0.12	0.59	1.04	2.17
01 Upper	0.01	0.10	0.29	0.83	0.12	0.60	0.18	0.65
02 Lower	0.03	0.16	0.38	0.60	0.09	0.25	0.11	0.25
02 Upper	0.00	0.03	0.21	0.44	0.30	0.66	0.12	0.28
03 Lower	0.39	0.74	0.81	1.92	0.66	1.34	0.16	0.35
03 Upper	0.08	0.22	0.31	0.99	0.01	0.07	0.04	0.13
04 Lower	0.00	0.03	0.17	0.48	0.01	0.08	0.00	0.05
04 Upper	0.00	0.03	0.20	0.71	0.01	0.08	0.12	0.64
05 Lower	0.00	0.04	0.00	0.05	0.01	0.05	0.05	0.26
05 Upper	0.64	2.55	0.02	0.11	0.05	0.14	0.02	0.08
06 Lower	0.09	0.26	0.02	0.10	0.02	0.09	0.00	0.05
06 Upper	0.00	0.03	0.00	0.03	0.01	0.06	0.02	0.07
07 Lower	0.09	0.30	0.01	0.06	0.00	0.06	-0.00	0.11
07 Upper	0.00	0.11	0.00	0.12	0.01	0.13	0.06	0.31
08 Lower	0.02	0.12	0.01	0.43	0.00	0.07	0.01	0.06
08 Upper	0.08	0.33	0.01	0.04	-0.01	0.06	0.00	0.06
09 Lower	0.05	0.23	0.01	0.07	0.00	0.05	-0.01	0.09
09 Upper	0.07	0.38	0.00	0.06	-0.00	0.09	0.00	0.06
10 Lower	0.18	0.53	0.00	0.03	0.01	0.08	0.00	0.07
10 Upper	0.09	0.28	0.00	0.04	0.02	0.09	0.01	0.07

Table S2. Hausdorff’s Distance Data table of augmented scans (Lab scanner considered standard)

Sample	**IOS1 + OP1**		**IOS1 + OP2**		**IOS2 + OP1**		**IOS2 + OP2**	
	Mean	*SD*	*Mean*	*SD*	*Mean*	*SD*	*Mean*	*SD*
3D printed lower	0.85	0.88	0.91	0.96	1.13	1.06	1.22	1.12
3D printed upper	0.64	1.21	0.58	1.15	0.32	0.74	0.55	1.11
01 Lower	0.25	0.49	0.15	0.43	0.03	0.17	0.16	0.43
01 Upper	0.42	0.68	0.40	0.70	0.82	1.01	0.03	0.19
02 Lower	0.06	0.27	0.04	0.24	0.05	0.22	0.02	0.13
02 Upper	0.00	0.10	0.02	0.13	0.25	0.49	0.00	0.05
03 Lower	0.07	0.31	0.28	0.64	0.02	0.13	0.47	0.69
03 Upper	0.03	0.19	0.03	0.17	0.09	0.31	0.01	0.11
04 Lower	0.06	0.32	0.17	0.49	0.15	0.47	0.09	0.34
04 Upper	0.00	0.06	0.04	0.26	0.25	0.58	0.09	0.41
05 Lower	0.33	0.74	0.13	0.44	0.31	0.63	0.06	0.26
05 Upper	0.04	0.24	0.03	0.18	0.05	0.27	0.01	0.11
06 Lower	0.27	0.57	0.04	0.20	0.05	0.26	0.07	0.30
06 Upper	0.16	0.50	0.02	0.16	0.00	0.06	0.03	0.23
07 Lower	0.04	0.21	0.04	0.27	0.19	0.51	0.20	0.52
07 Upper	0.02	0.29	0.01	0.14	0.01	0.11	0.01	0.12
08 Lower	0.22	0.57	0.03	0.21	0.26	0.67	0.23	0.64
08 Upper	0.18	0.55	0.01	0.09	0.02	0.19	0.00	0.06
09 Lower	0.01	0.07	0.05	0.29	0.09	0.31	0.05	0.29
09 Upper	0.00	0.07	0.00	0.09	0.00	0.80	0.00	0.06
10 Lower	0.01	0.11	0.04	0.28	0.01	0.15	0.06	0.31
10 Upper	0.04	0.23	0.02	0.14	0.00	0.08	0.02	0.16

Table S3. Hausdorff's Distance Data table comparing native dataset vs augmented dataset

Sample	IOS1 + OP1		IOS1 + OP2		IOS2 + OP1		IOS2 + OP2	
	Mean	*SD*	*Mean*	*SD*	*Mean*	*SD*	*Mean*	*SD*
Standard Scan Lower	-0.01	1.27	-0.19	0.56	-0.25	0.24	-0.25	0.26
Standard Scan Upper	-0.24	0.24	-0.25	0.24	-0.25	0.25	-0.25	0.25
01 Lower	-0.34	1.03	-0.19	0.62	-0.25	0.24	-0.25	0.25
01 Upper	-0.28	0.91	-0.15	0.31	-0.25	0.25	-0.25	0.25
02 Lower	-0.18	0.41	-0.24	0.24	0.04	0.32	-0.24	0.25
02 Upper	-0.35	1.53	-0.03	0.44	-0.24	0.24	-0.25	0.25
03 Lower	-0.24	0.24	-0.33	0.69	-0.24	0.25	-0.24	0.24
03 Upper	-0.24	0.24	-0.40	0.39	-0.24	0.24	-0.24	0.25
04 Lower	-0.24	0.24	-0.04	0.94	0.11	1.15	-0.24	0.24
04 Upper	-0.25	0.24	-0.25	0.25	-0.25	0.25	-0.25	0.25
05 Lower	-0.06	0.64	0.63	1.08	-0.25	0.24	-0.25	0.24
05 Upper	-0.22	0.37	0.39	0.65	-0.25	0.25	-0.25	0.25
06 Lower	-0.09	0.73	-0.11	0.40	-0.24	0.24	-0.24	0.24
06 Upper	-0.16	1.66	-0.02	0.28	-0.24	0.24	-0.24	0.24
07 Lower	-0.26	0.60	-0.12	0.69	-0.25	0.25	-0.24	0.25
07 Upper	-0.25	0.24	-0.26	0.33	-0.25	0.25	-0.25	0.24
08 Lower	-0.24	0.24	-0.25	0.24	-0.08	0.79	-0.25	0.25
08 Upper	-0.25	0.25	-0.21	0.51	-0.25	0.24	-0.25	0.25
09 Lower	-0.29	0.83	-0.47	1.17	-1.51	2.67	-0.25	0.24
09 Upper	-0.38	1.34	-0.80	0.92	-0.25	0.25	-0.25	0.25
10 Lower	-0.29	0.69	-0.24	0.25	-0.25	0.25	-0.25	0.25
10 Upper	-0.34	0.64	0.57	0.67	-0.25	0.25	-0.25	0.25

Table S4. Root mean square error (RMSE) value of native scans (Lab scanner considered standard)

Sample	IOS1 + OP1	IOS1 + OP2	IOS2 + OP1	IOS2 + OP2
3D printed lower	45.592983	38.9476	31.056149	32.168015
3D printed upper	42.052273	37.73743	35.311436	39.21446
01 Lower	23.070724	26.402708	35.60772	33.52077
01 Upper	31.969397	31.011934	32.658222	32.65341
02 Lower	23.083122	29.666405	27.338987	27.462332
02 Upper	26.099634	27.454845	25.079912	32.09955
03 Lower	29.106987	29.765272	24.014301	24.339241
03 Upper	32.56765	29.22785	32.80361	27.956861
04 Lower	33.072926	31.765627	24.806719	31.952013
04 Upper	32.69865	32.925186	32.43568	34.8358
05 Lower	23.372362	28.37013	33.99208	28.571321
05 Upper	34.46011	34.004467	36.85757	28.254255
06 Lower	36.63631	32.888386	29.925455	33.902706
06 Upper	32.107513	35.434784	35.682487	34.608845
07 Lower	39.02781	38.91666	35.74593	36.64801
07 Upper	32.42329	38.787815	42.57793	44.75692
08 Lower	44.34486	43.341362	36.067356	34.568687
08 Upper	38.134407	42.669678	36.4228	38.708588
09 Lower	44.88963	36.243538	40.705765	41.046017
09 Upper	42.817547	43.990326	42.82798	40.398357
10 Lower	40.534733	40.532368	35.96939	38.32908
10 Upper	42.13337	43.919724	31.883114	39.756035

Table S5. Root mean square error (RMSE) value of augmented scans (Lab scanner considered standard)

**Sample**	**IOS1 + OP1**	**IOS1 + OP2**	**IOS2 + OP1**	**IOS2 + OP2**
3D printed lower	40.876526	35.67165	38.4983	41.759415
3D printed upper	37.7054	37.7874	36.2992	33.30922
01 Lower	29.085054	30.982618	29.137104	28.051573
01 Upper	29.311043	31.835842	30.377565	28.87961
02 Lower	25.306435	24.397924	28.33862	26.252106
02 Upper	24.893188	26.910402	26.556591	25.77872
03 Lower	28.179375	26.505123	26.551205	28.077421
03 Upper	27.749464	29.717903	28.262337	27.2906
04 Lower	28.944967	30.578037	27.357243	26.916603
04 Upper	28.906042	32.10417	30.43464	28.674036
05 Lower	27.283772	30.540447	27.920826	28.335173
05 Upper	28.718754	29.854498	30.50984	27.815712
06 Lower	36.119183	36.08557	29.566982	30.200222
06 Upper	32.807705	33.448742	31.276346	32.635025
07 Lower	33.718903	41.9308	37.44348	41.159588
07 Upper	41.12976	38.338356	35.214233	37.727325
08 Lower	42.583145	38.304127	34.7351	39.37779
08 Upper	39.3416	40.844105	34.54722	37.043526
09 Lower	33.9195	37.704353	34.870102	36.272152
09 Upper	42.10982	41.194107	38.42807	38.704857
10 Lower	35.2847	31.48099	33.068676	28.518589
10 Upper	34.283962	35.07485	34.43549	33.76017

Heatmaps of Native scans generated through Hausdorff's distance evaluation

IOS1 + OP1

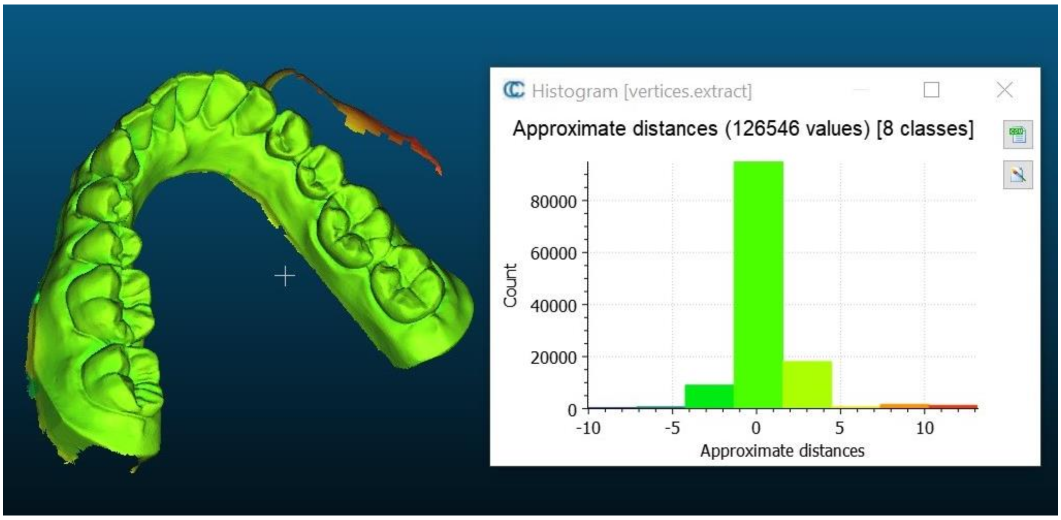

Fig 1: J standard model lower jaw.

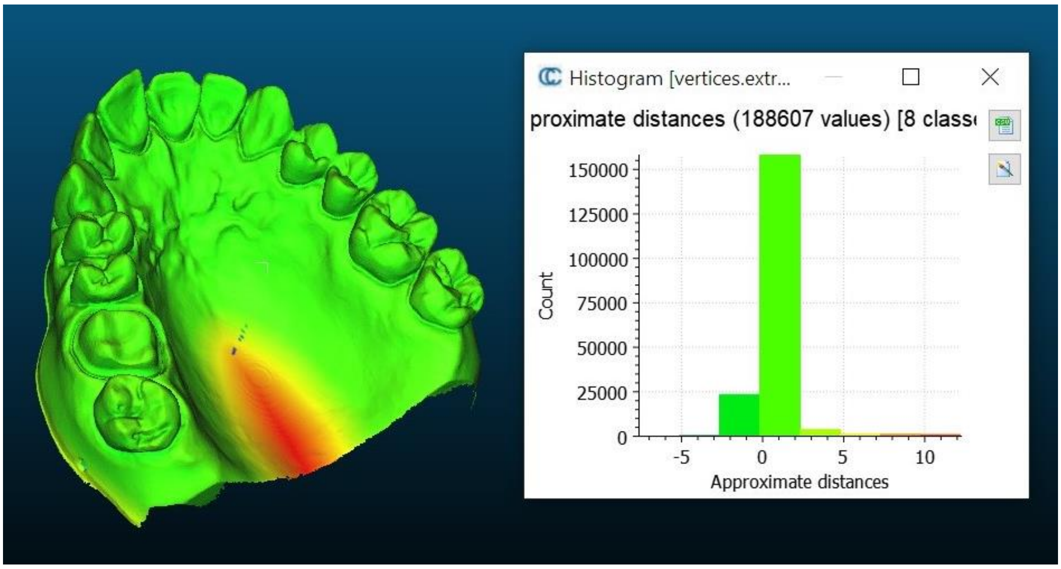

Fig 2: J standard model upper jaw .

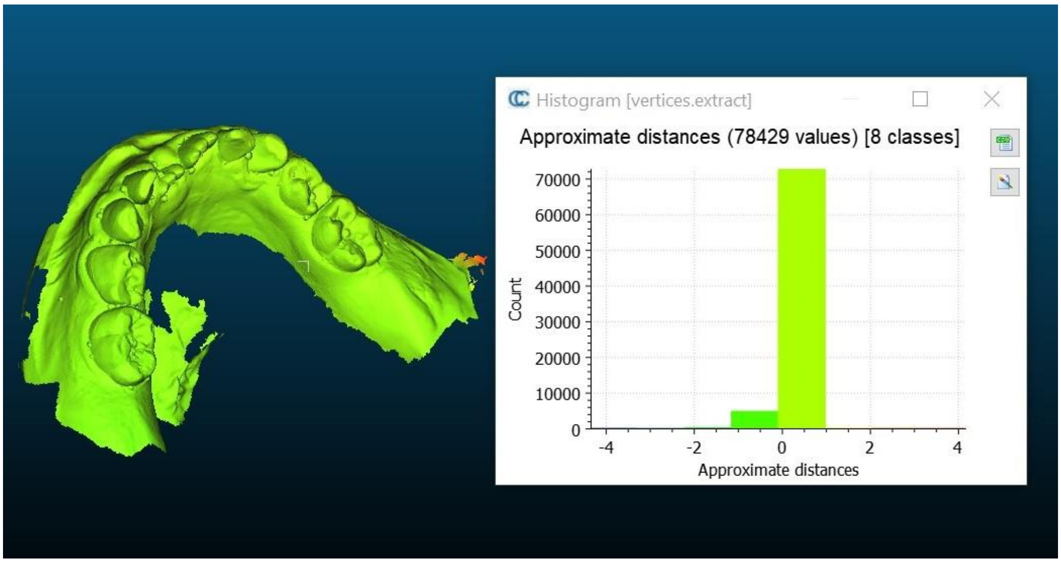

Fig 3: J01 lower jaw .

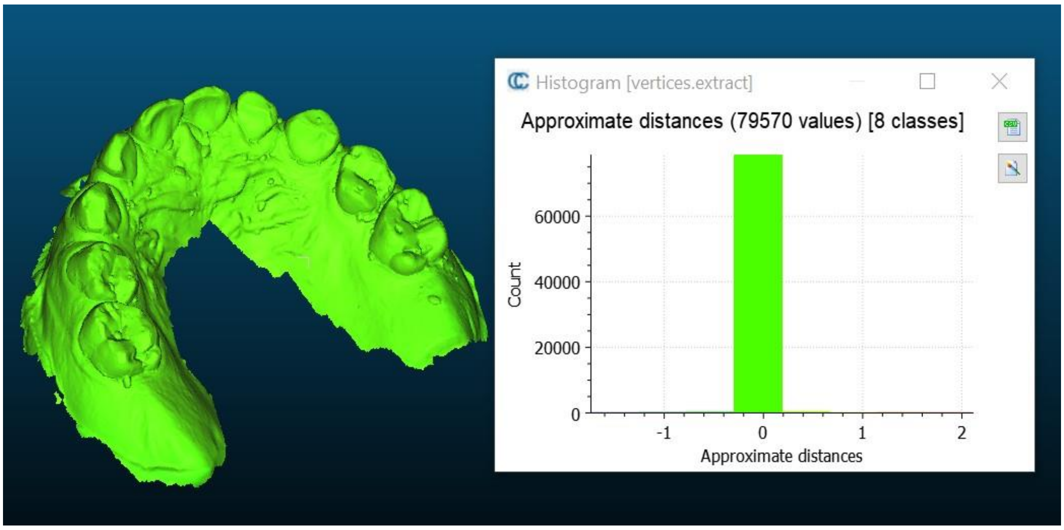

Fig 4: J01 upper jaw .

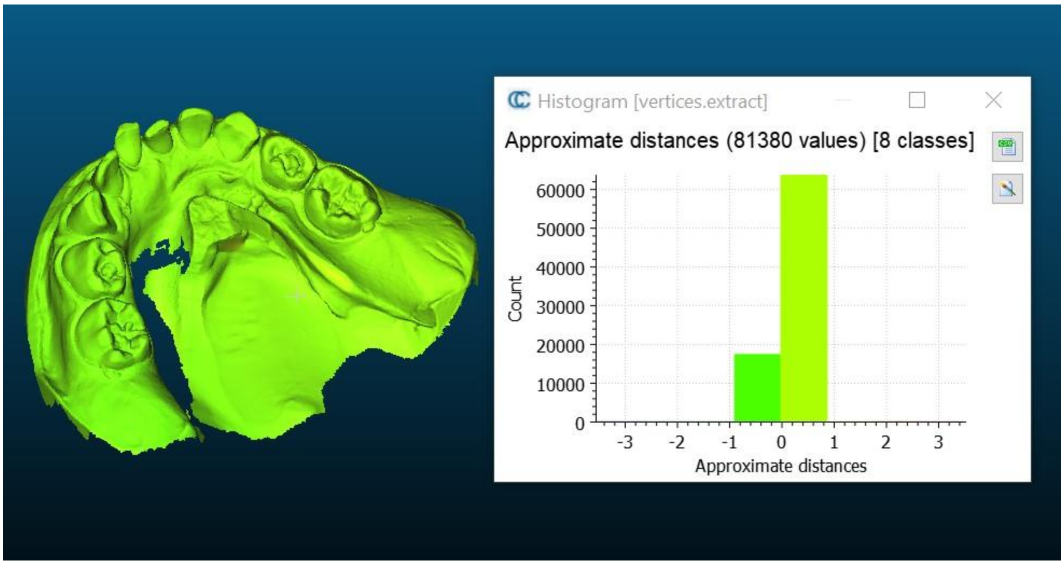

Fig 5: J02 lower jaw .

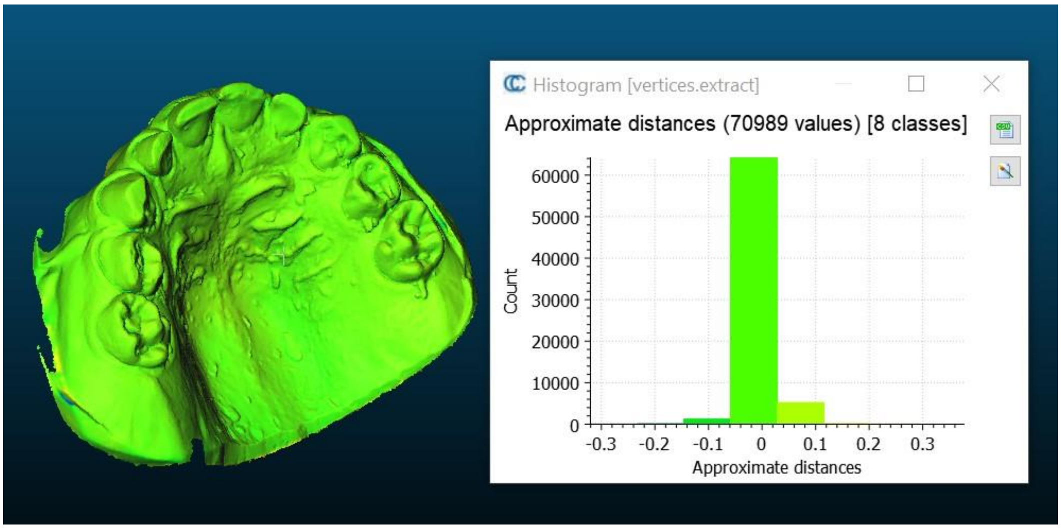

Fig 6: J02 upper jaw .

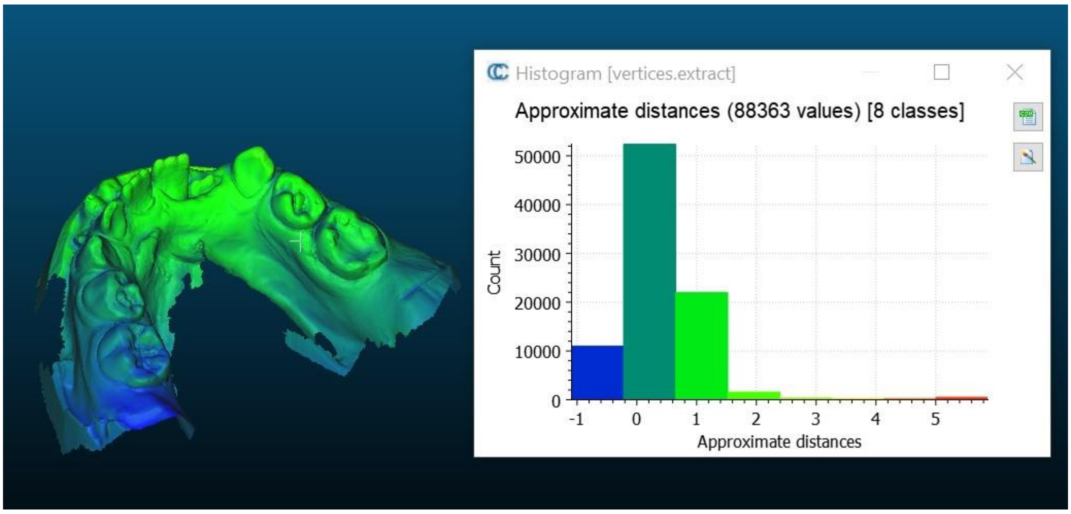

Fig 7: J03 lower jaw .

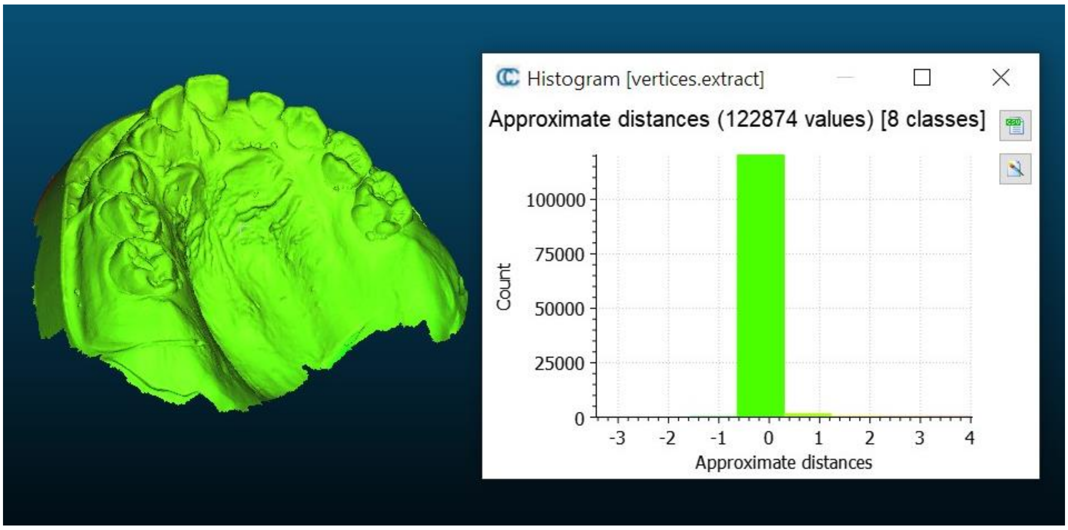

Fig 8: J03 upper jaw .

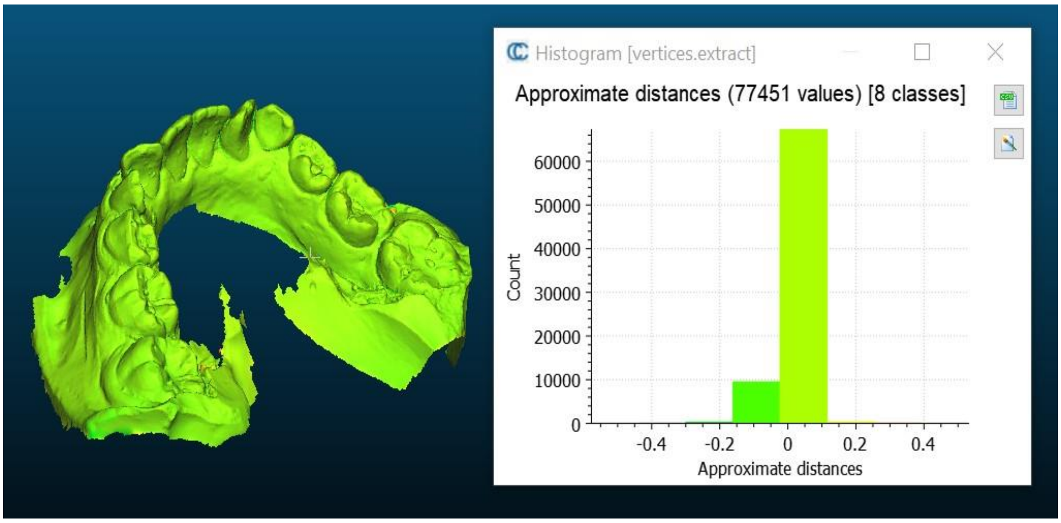

Fig 9: J04 lower jaw .

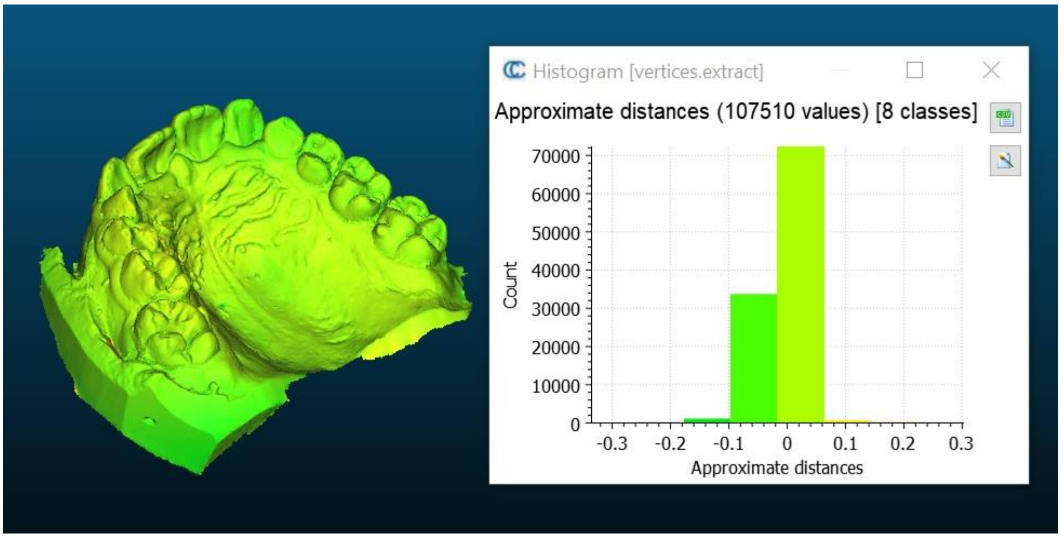

Fig 10: J04 upper jaw .

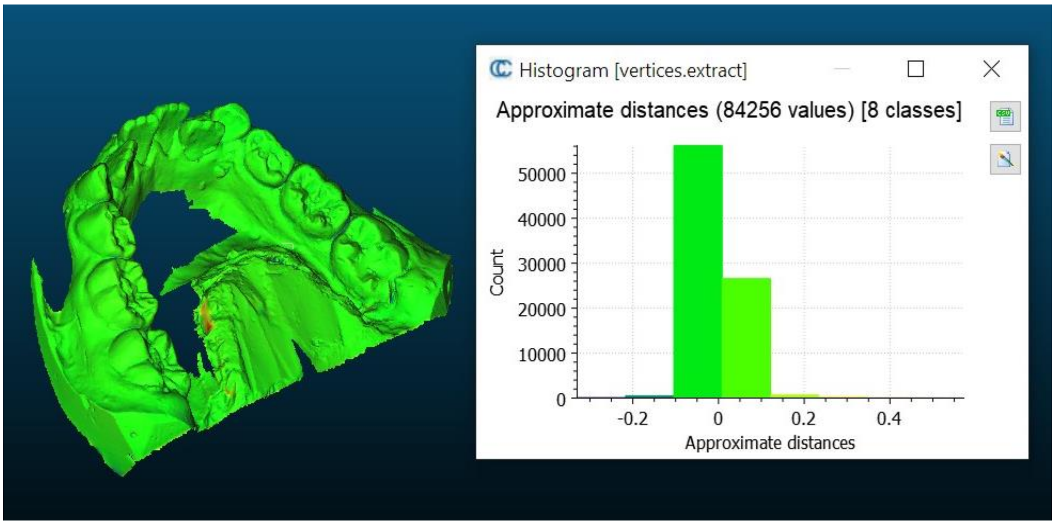

Fig 11: J05 lower jaw .

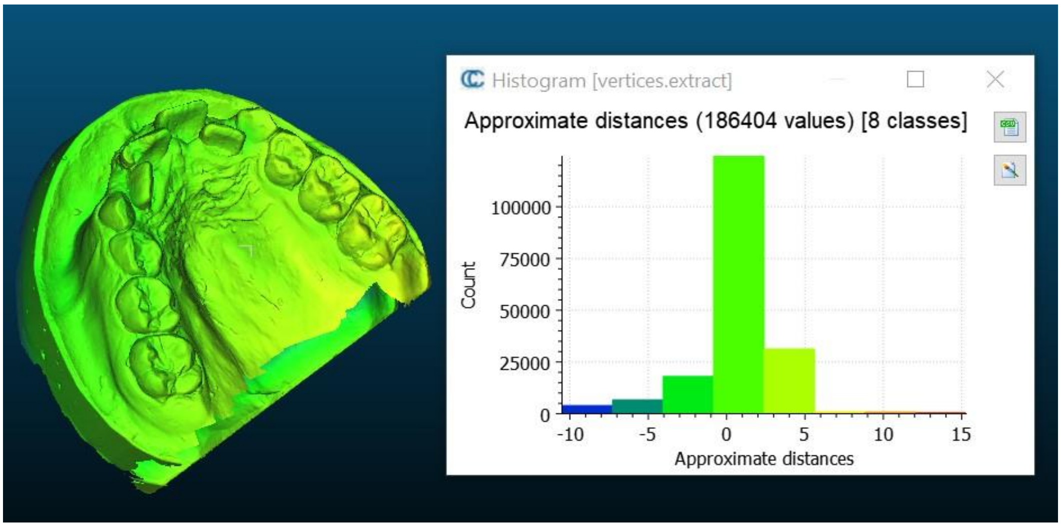

Fig 12: J05 upper jaw .

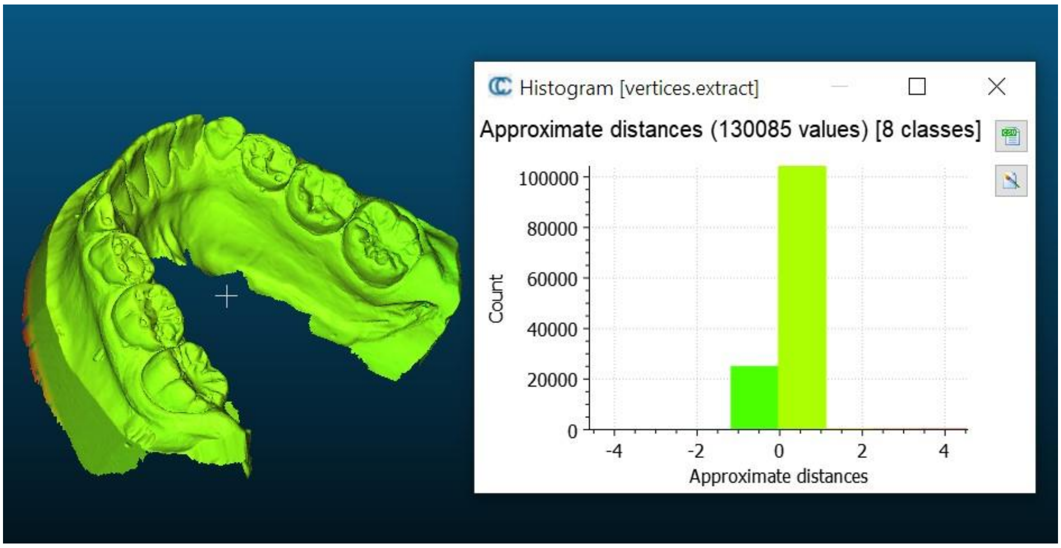

Fig 13: J06 lower jaw .

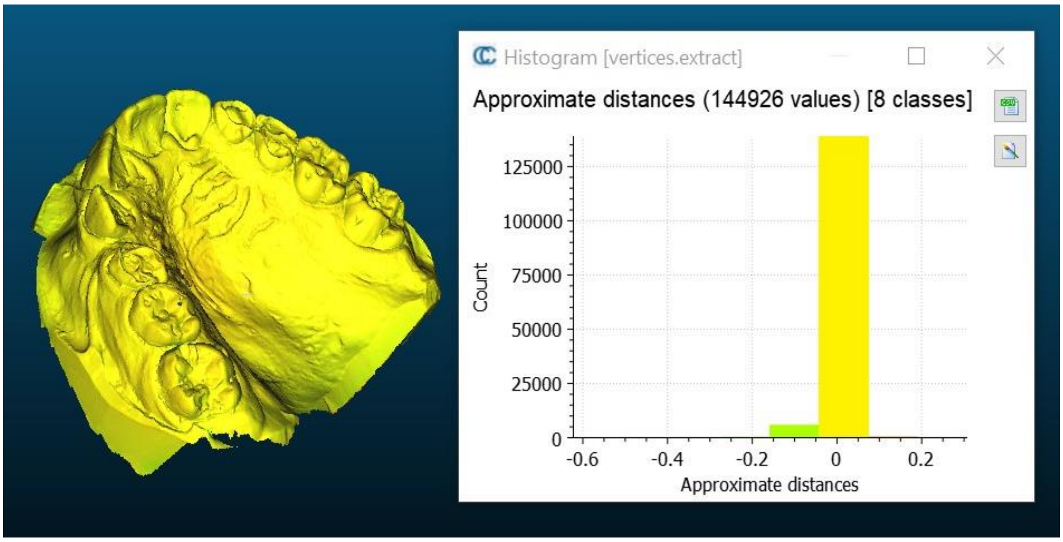

Fig 14: J06 upper jaw .

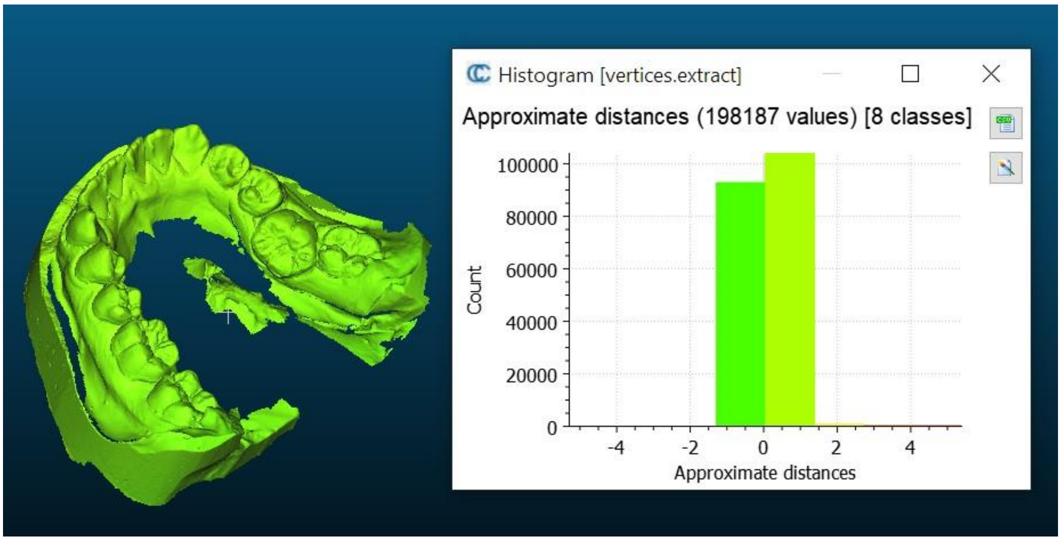

Fig 15: J07 lower jaw .

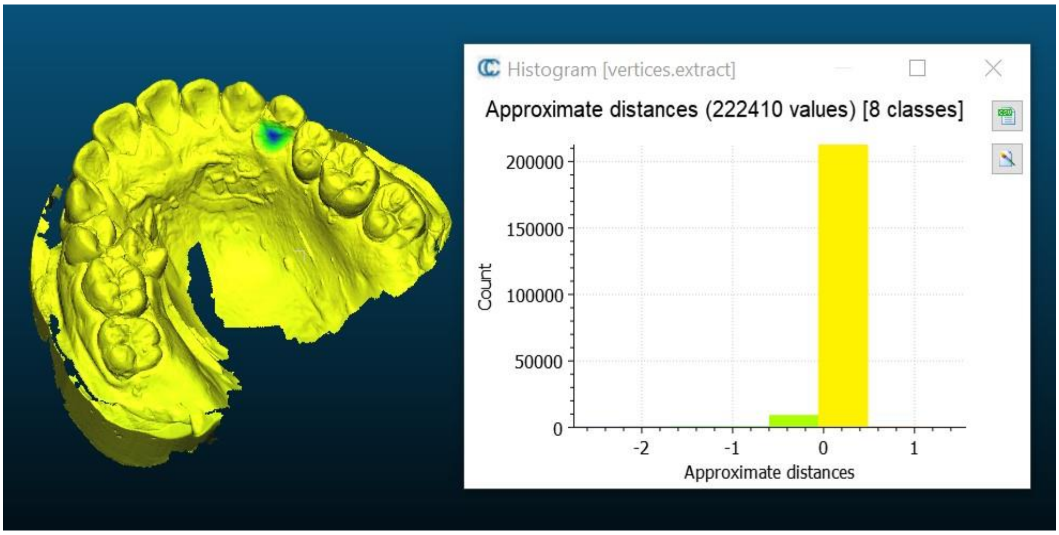

Fig 16: J07 upper jaw .

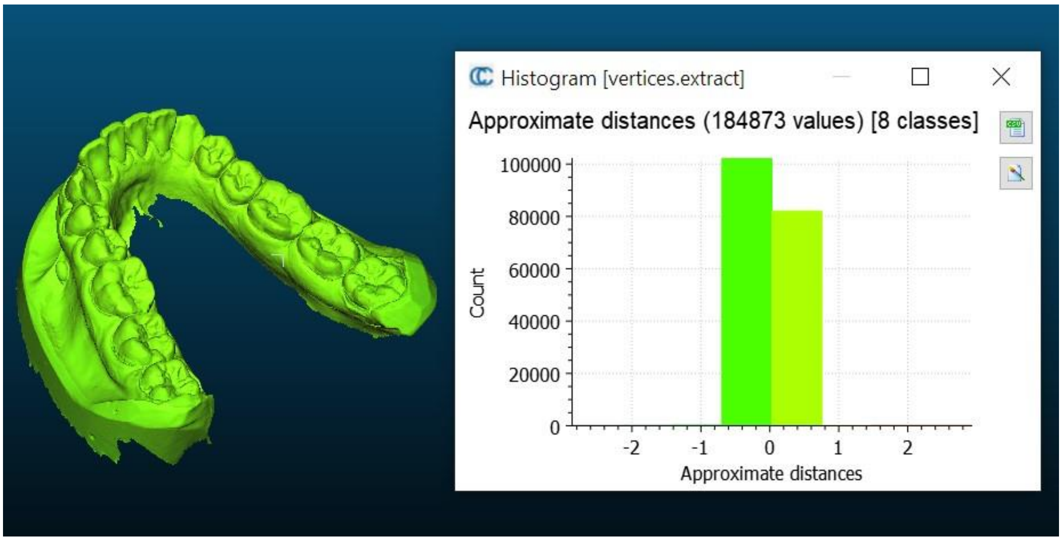

Fig 17: J08 lower jaw .

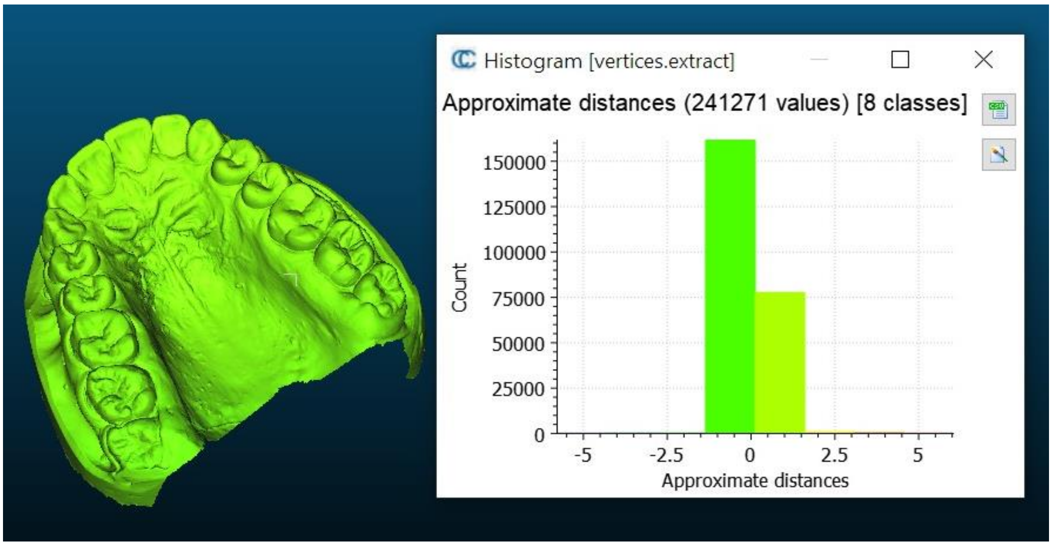

Fig 18: J08 upper jaw .

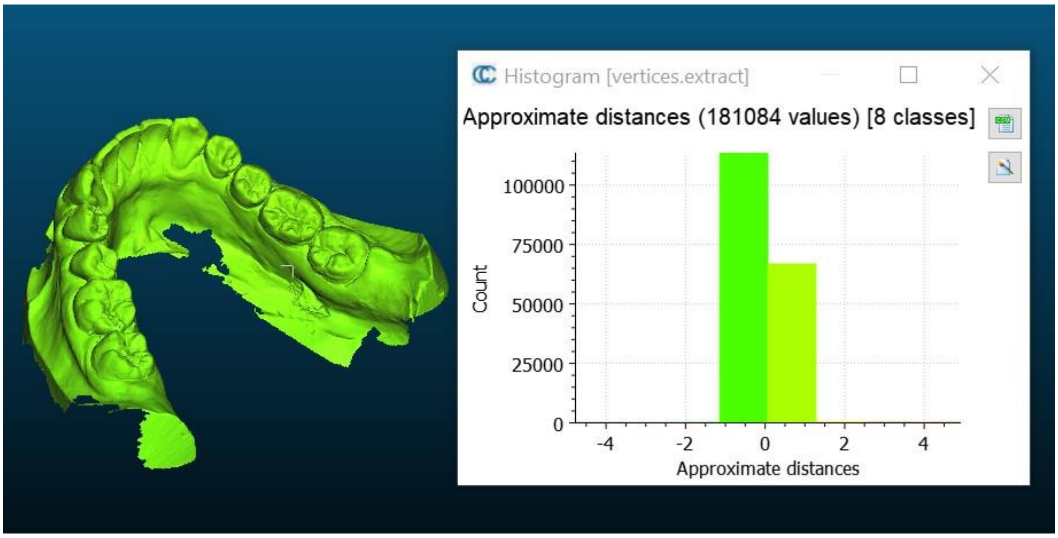

Fig 19: J09 lower jaw .

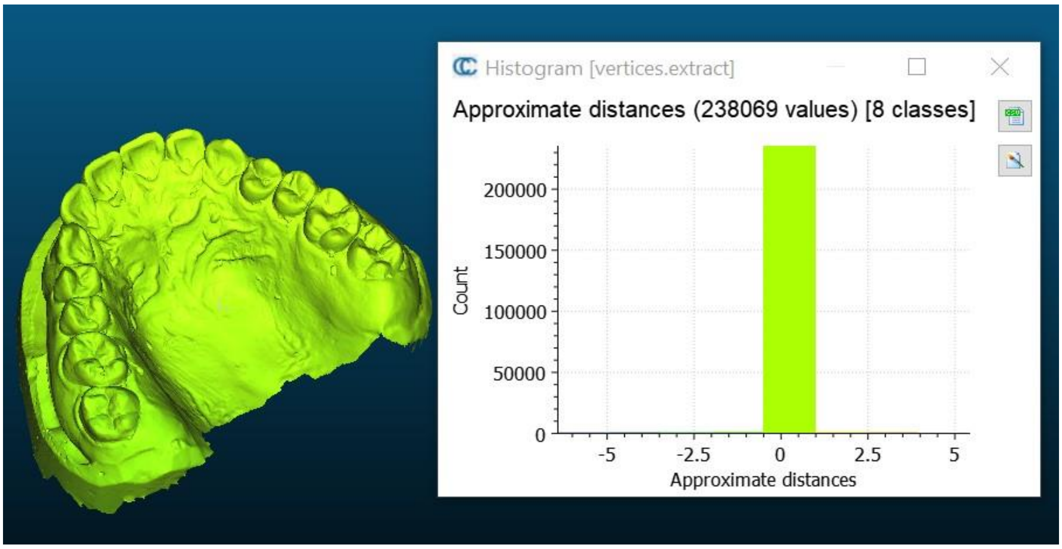

Fig 20: J09 upper jaw .

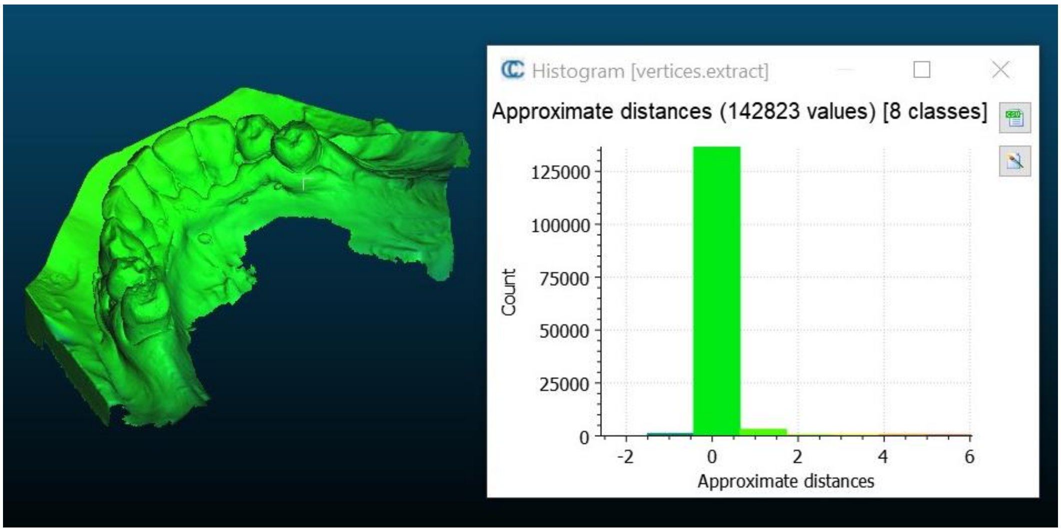

Fig 21: J10 lower jaw .

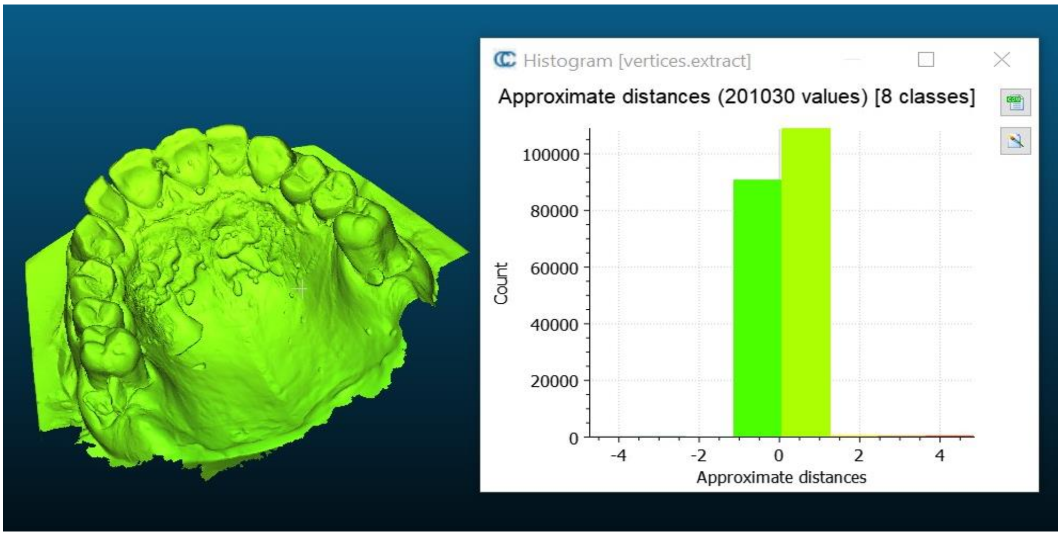

Fig 22: J10 upper jaw .

IOS1 + OP2

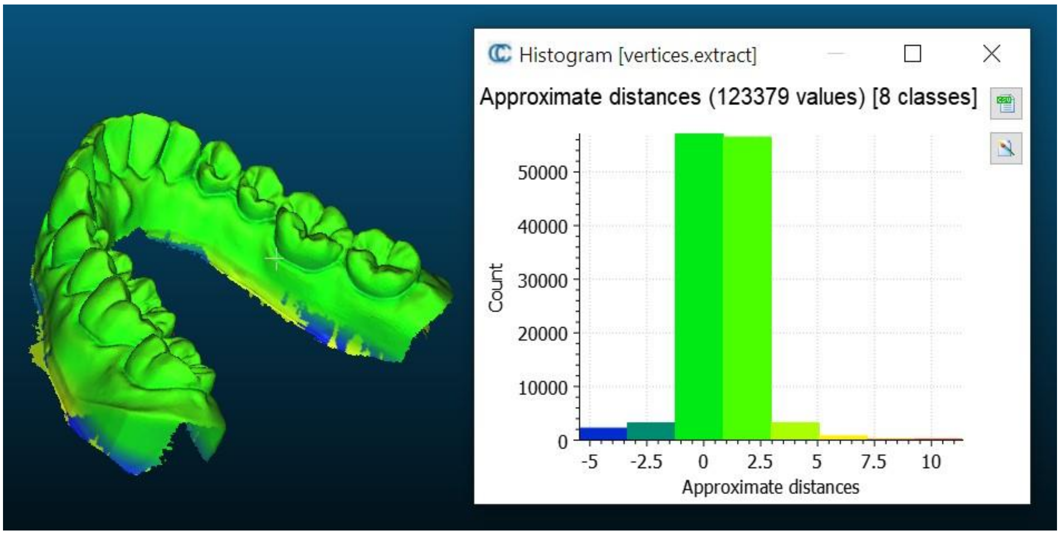

Fig 1: T standard model lower jaw .

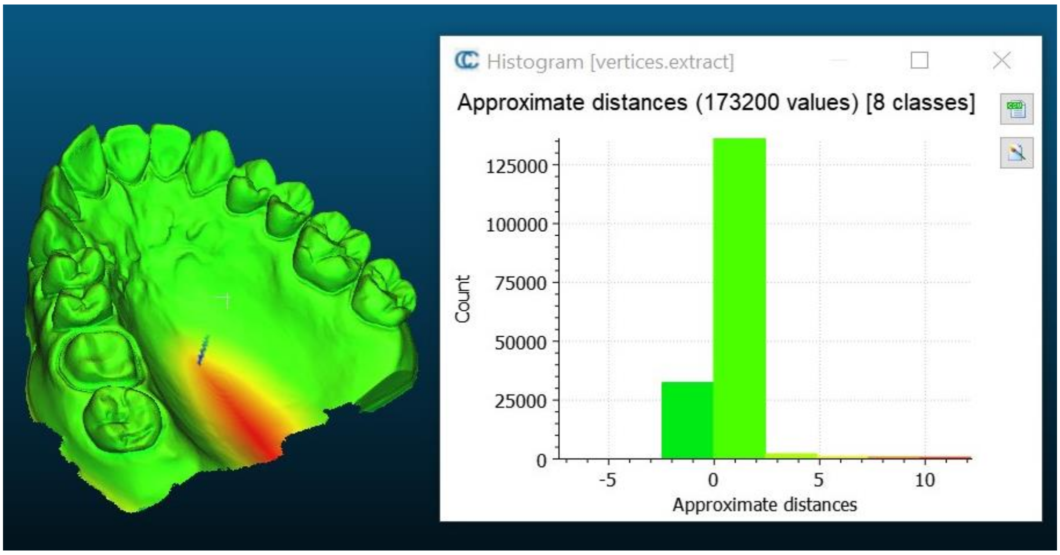

Fig 2: T standard model upper jaw .

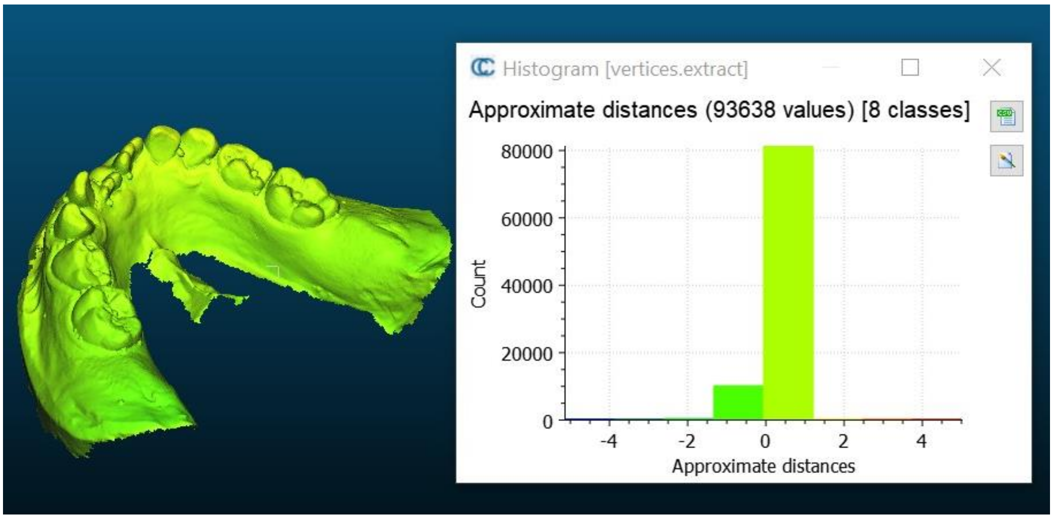

Fig 3: T01 lower jaw .

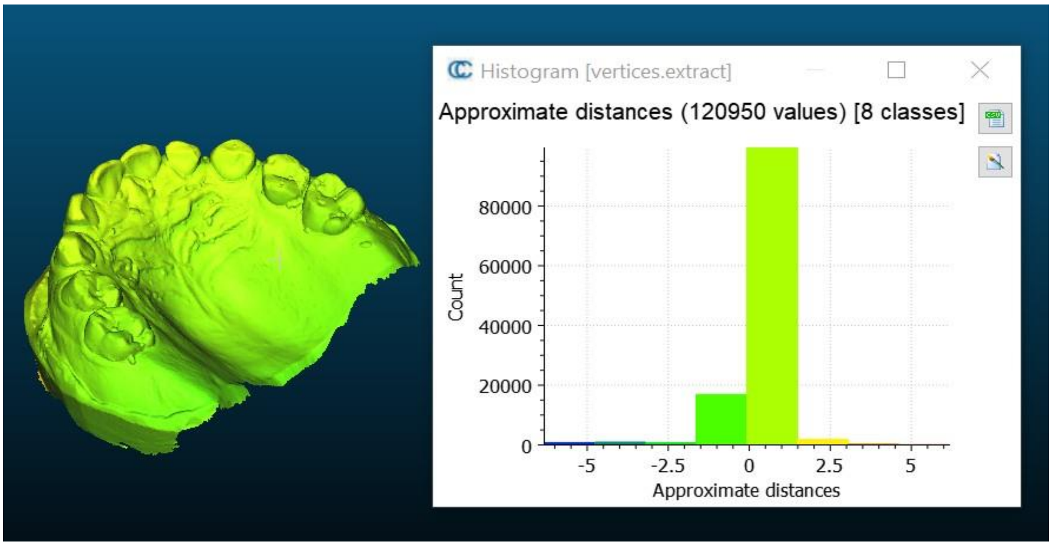

Fig 4: T01 upper jaw .

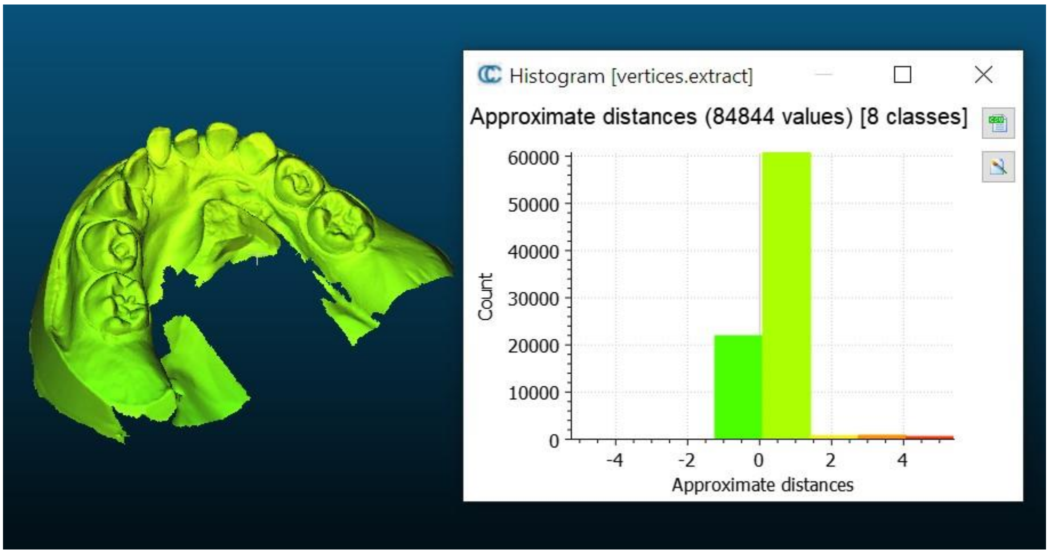

Fig 5: T02 lower jaw .

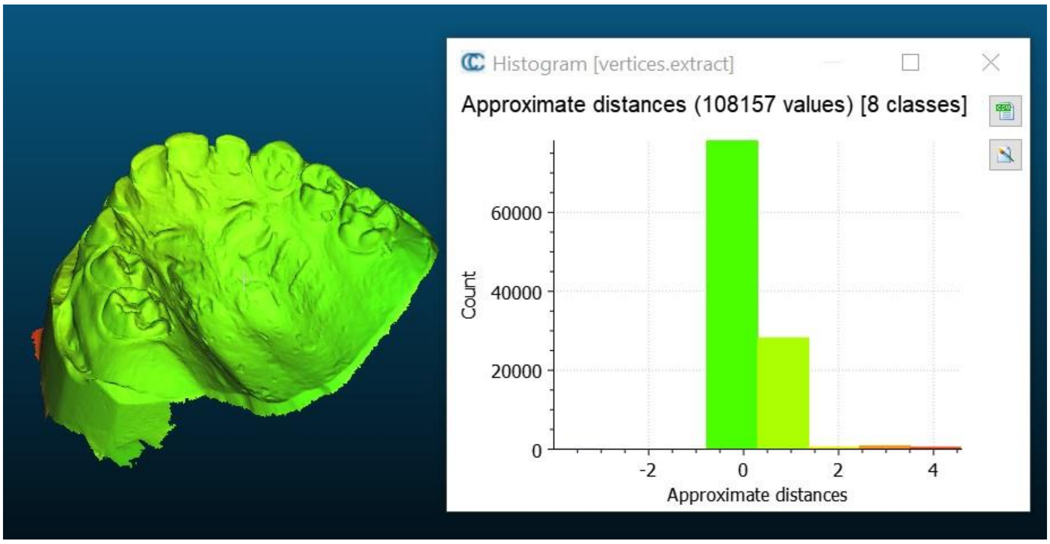

Fig 6: T02 upper jaw .

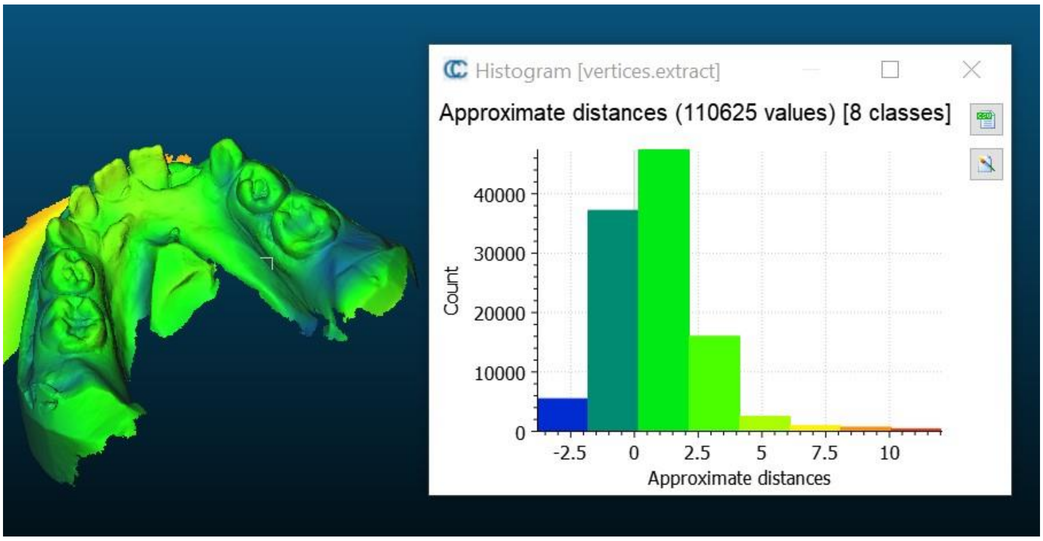

Fig 7: T03 lower jaw .

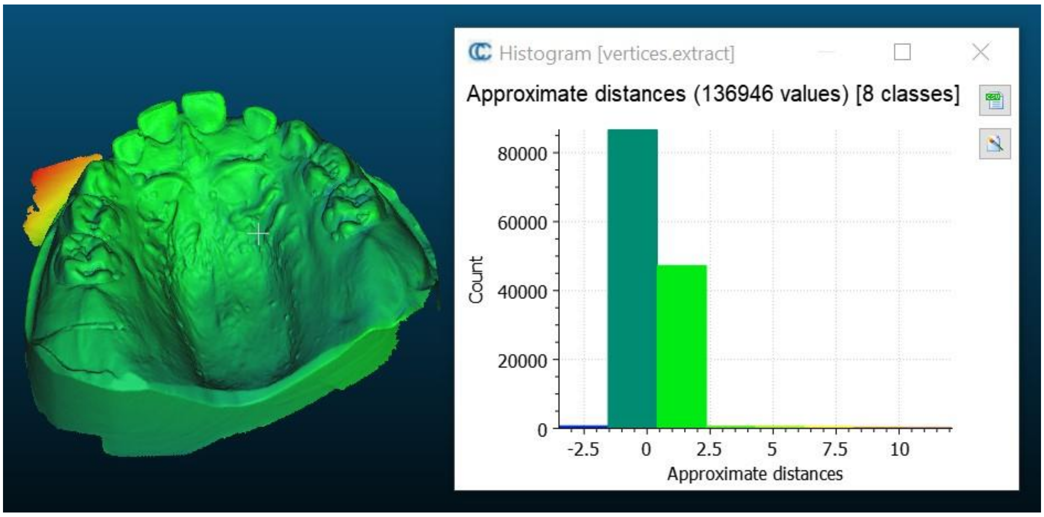

Fig 8: T03 upper jaw .

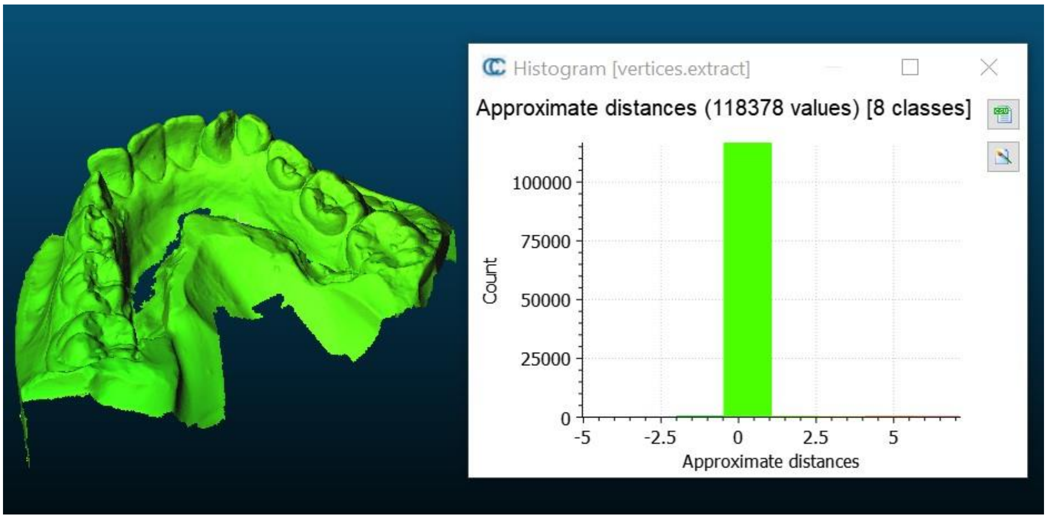

Fig 9: T04 lower jaw .

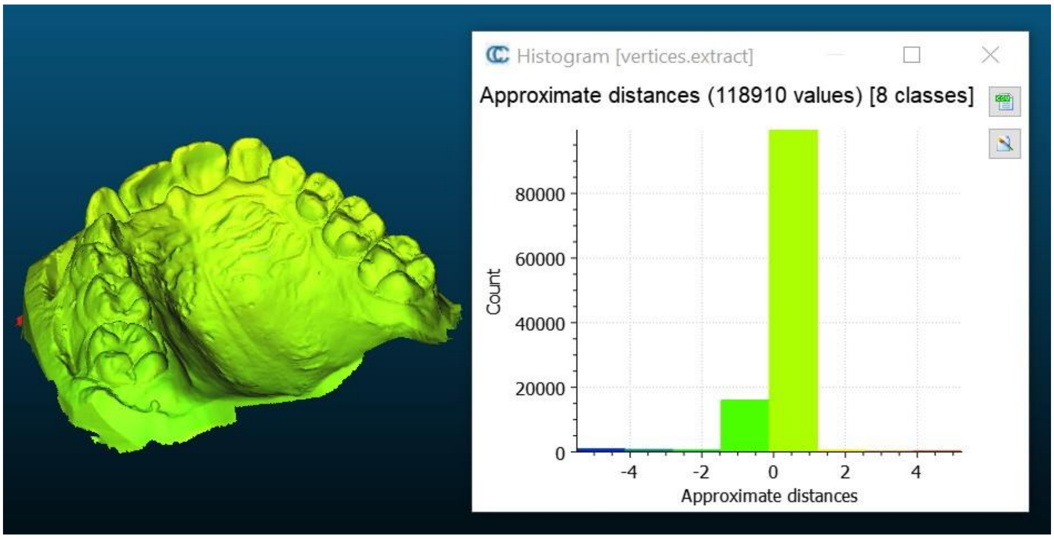

Fig 10: T04 upper jaw .

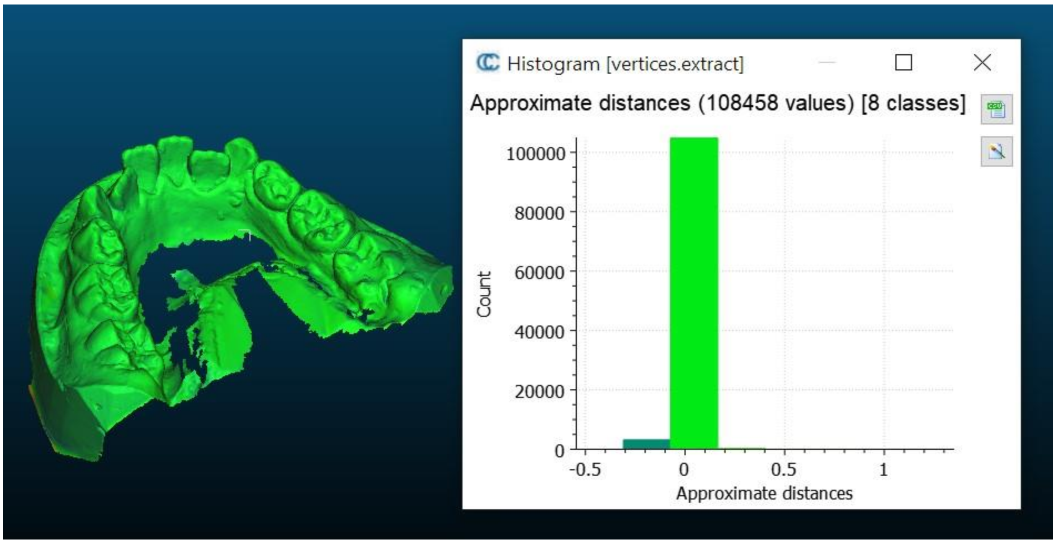

Fig 11: T05 lower jaw .

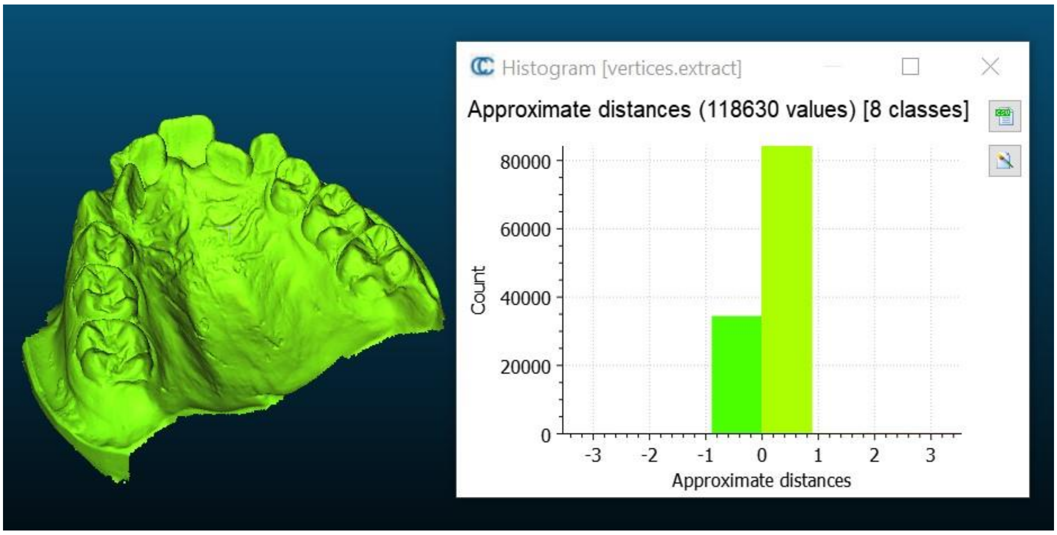

Fig 12: T05 upper jaw .

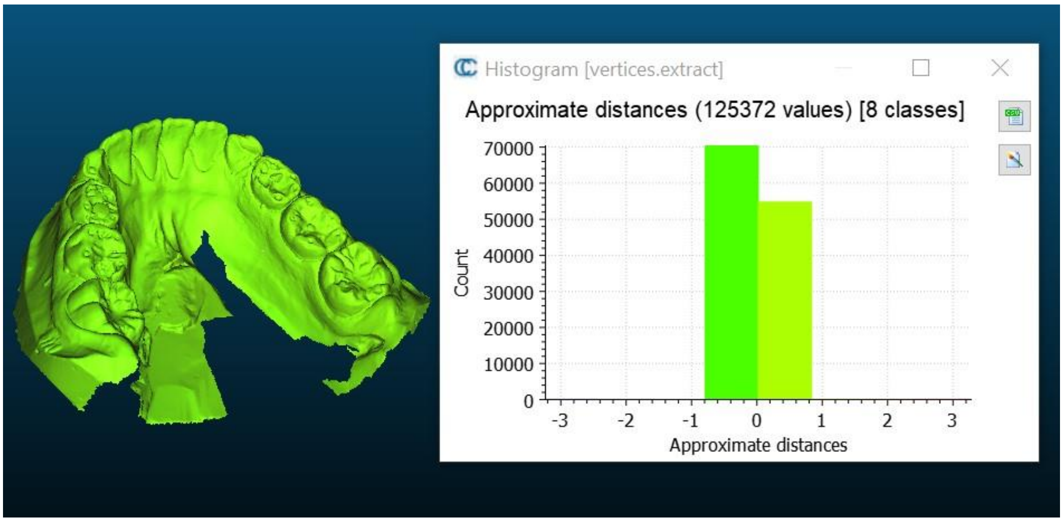

Fig 13: T06 lower jaw .

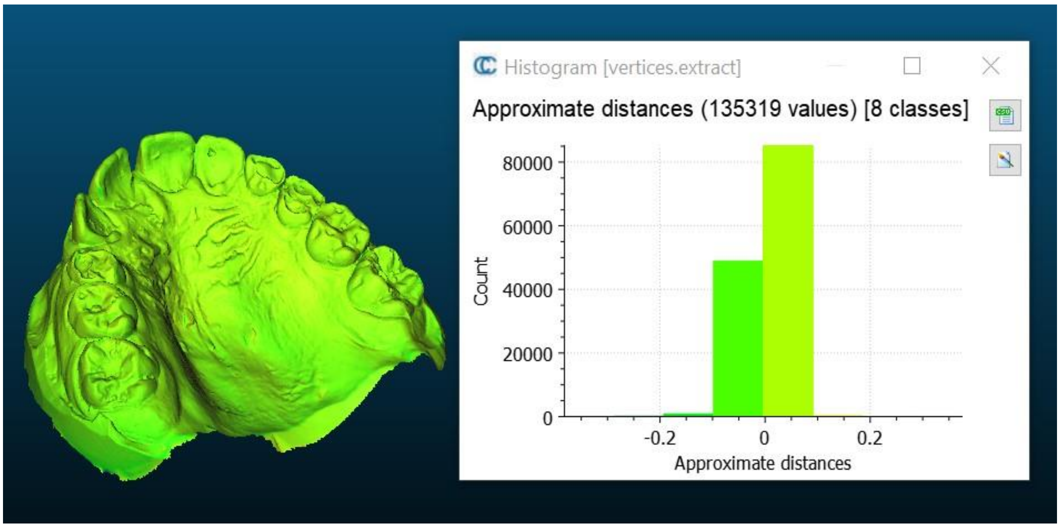

Fig 14: T06 upper jaw .

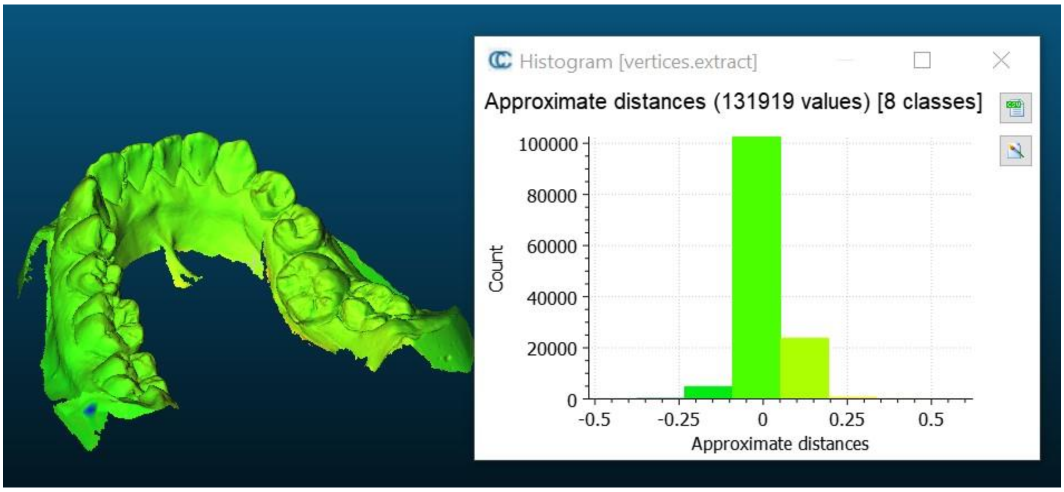

Fig 15: T07 lower jaw .

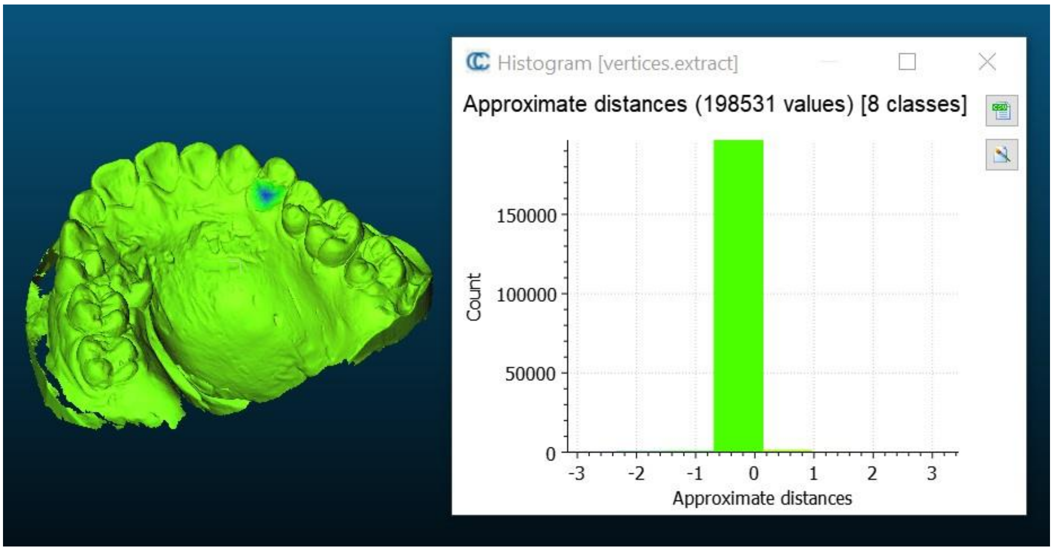

Fig 16: T07 upper jaw .

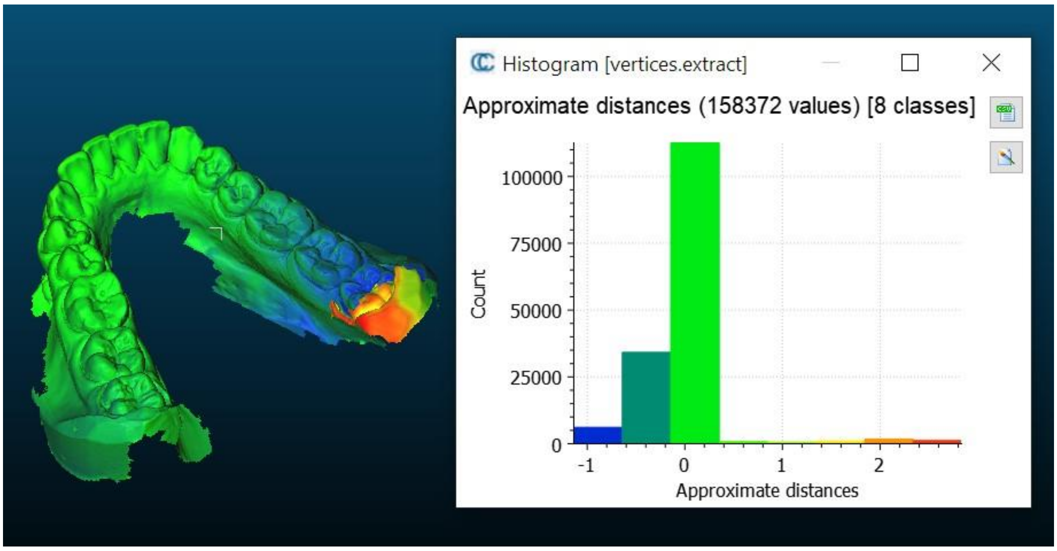

Fig 17: T08 lower jaw .

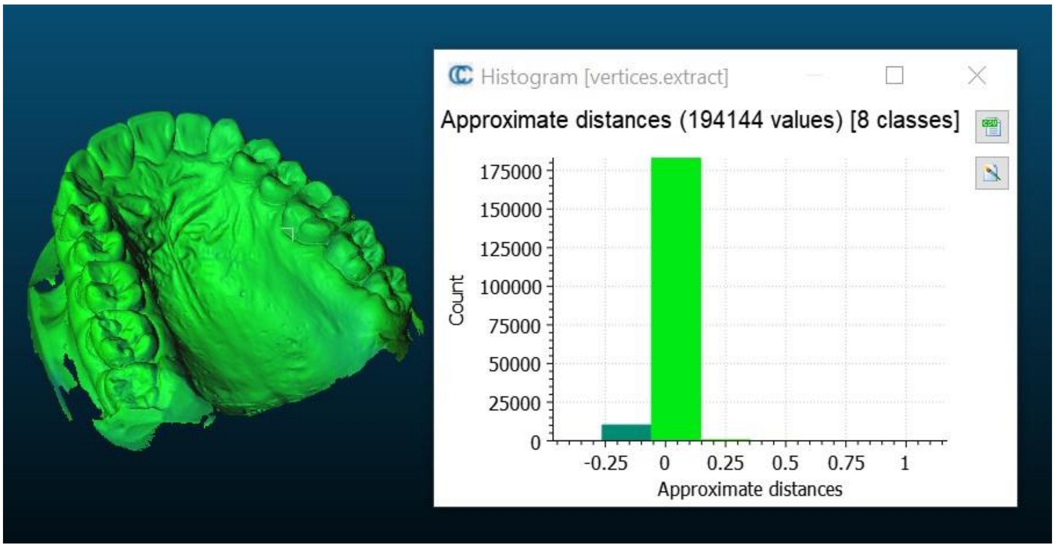

Fig 18: T08 upper jaw .

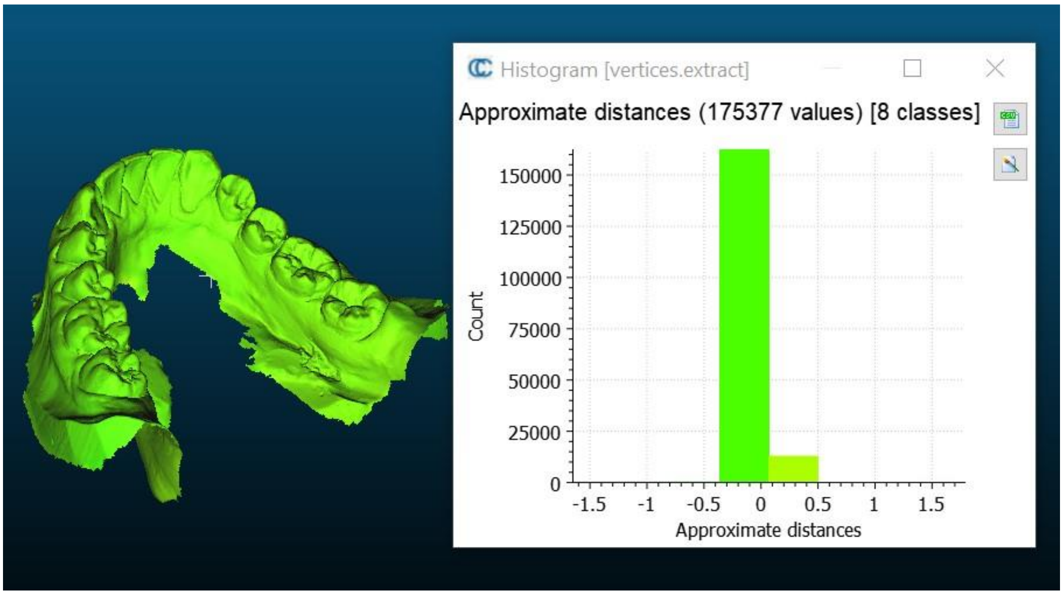

Fig 19: T09 lower jaw .

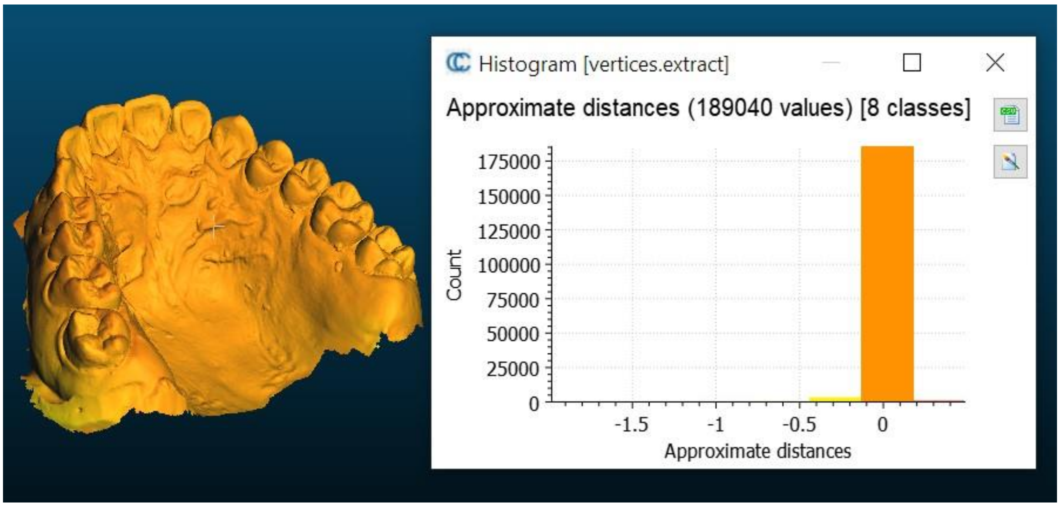

Fig 20: T09 upper jaw .

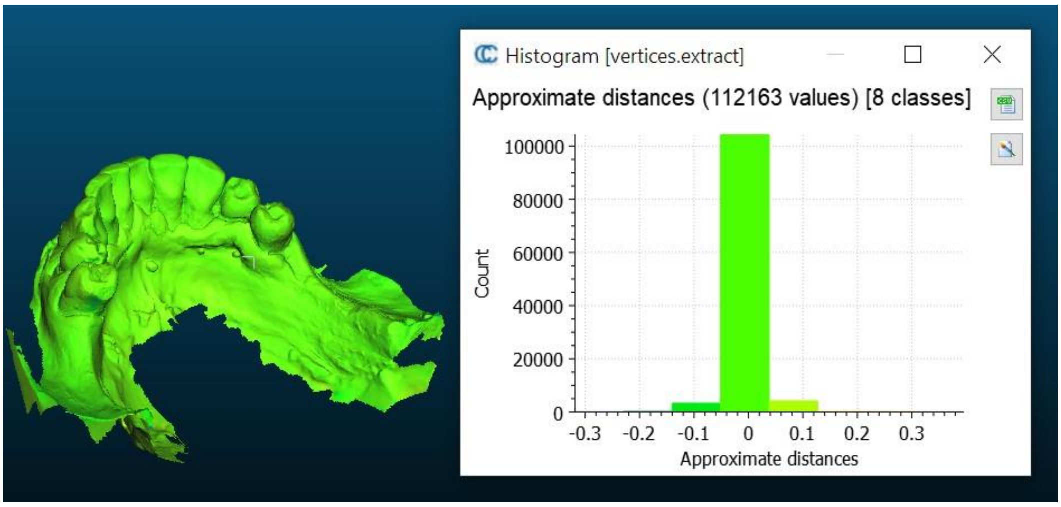

F21: T10 lower jaw .

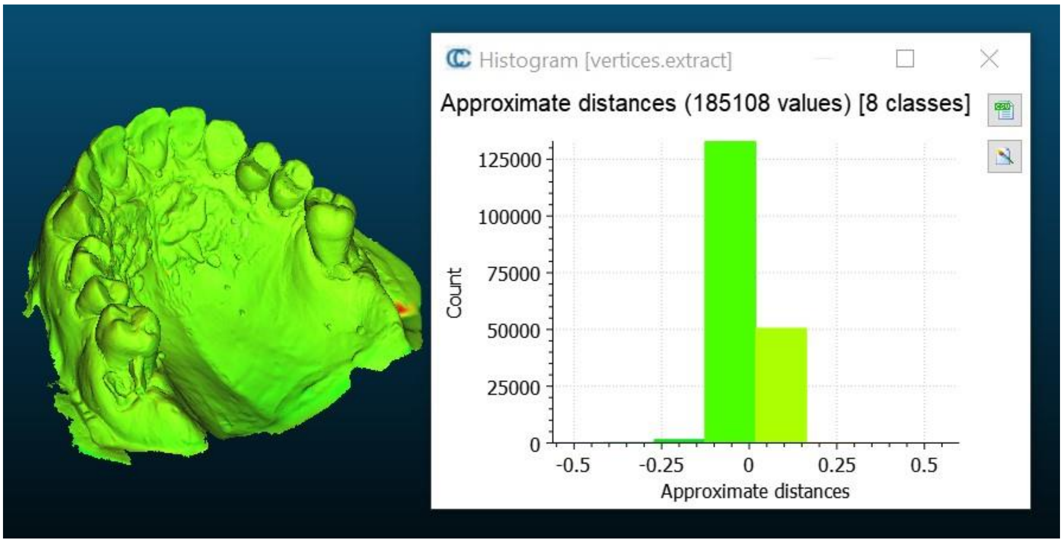

F22: T10 upper jaw .

IOS2 + OP1

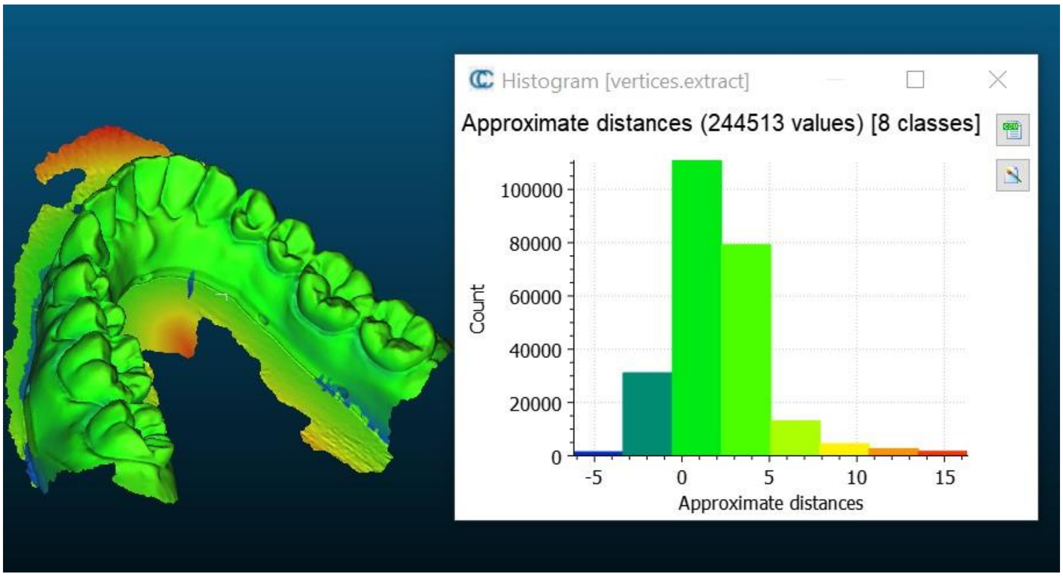

Fig 1: J standard model lower jaw .

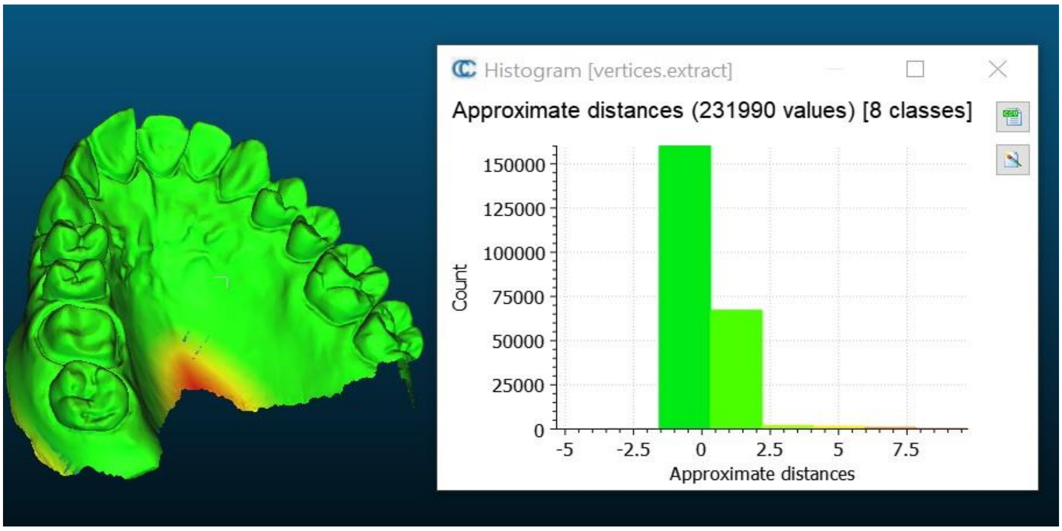

Fig 2: J standard model upper jaw .

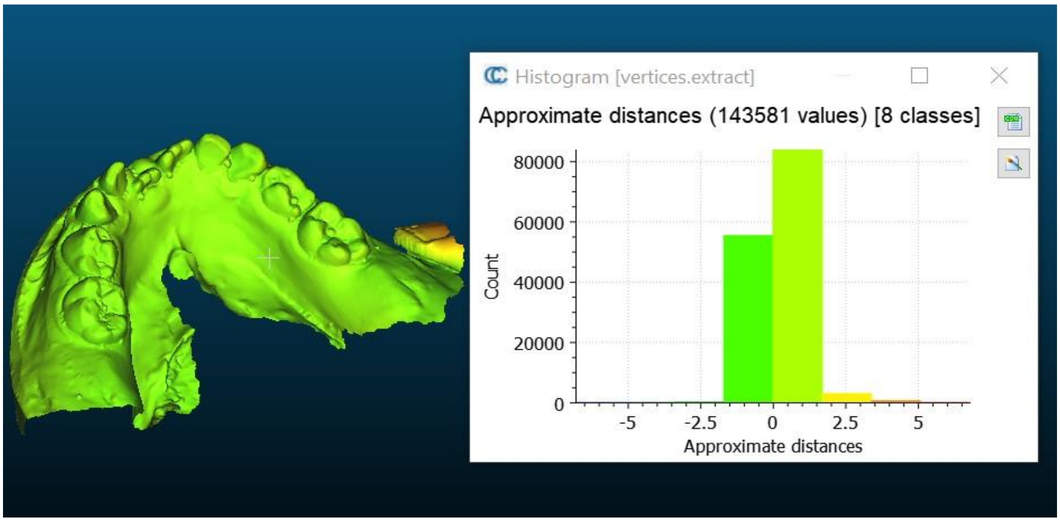

Fig 3: J01 lower jaw .

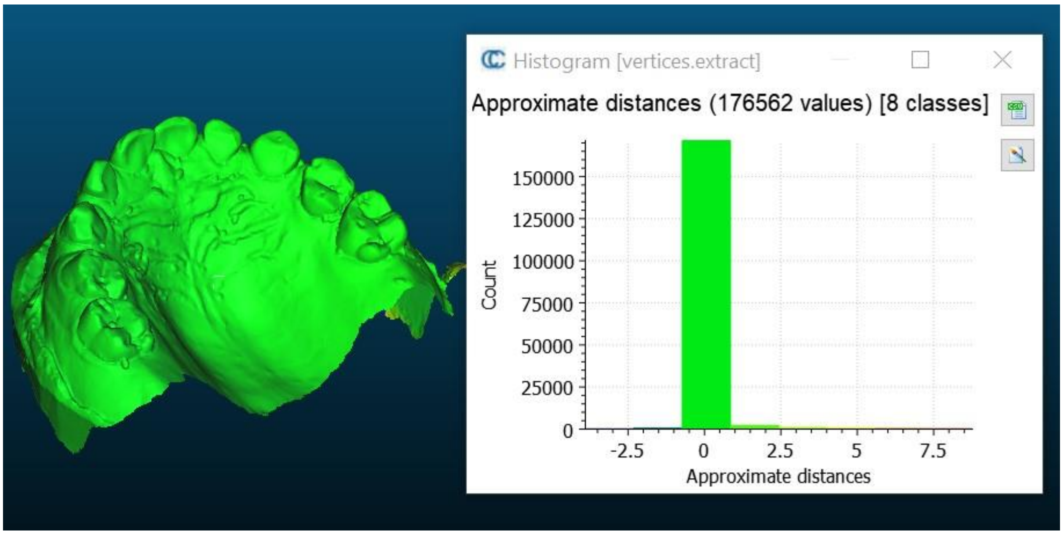

Fig 4: J01 upper jaw .

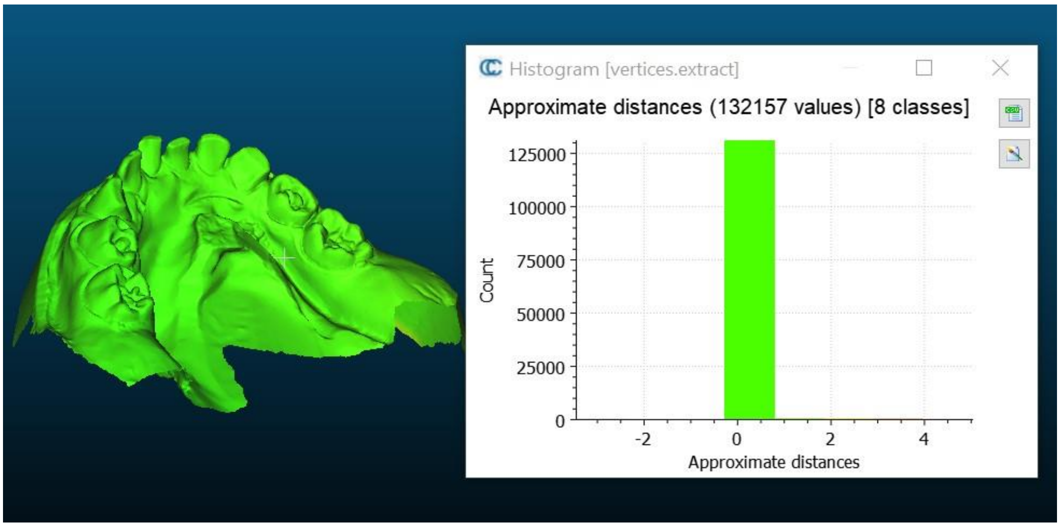

Fig 5: J02 lower jaw .

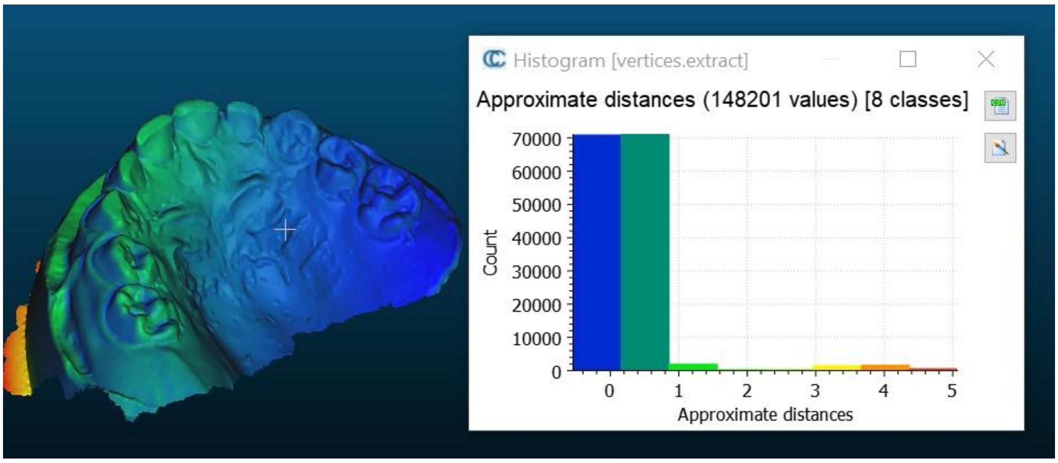

Fig 6: J02 upper jaw .

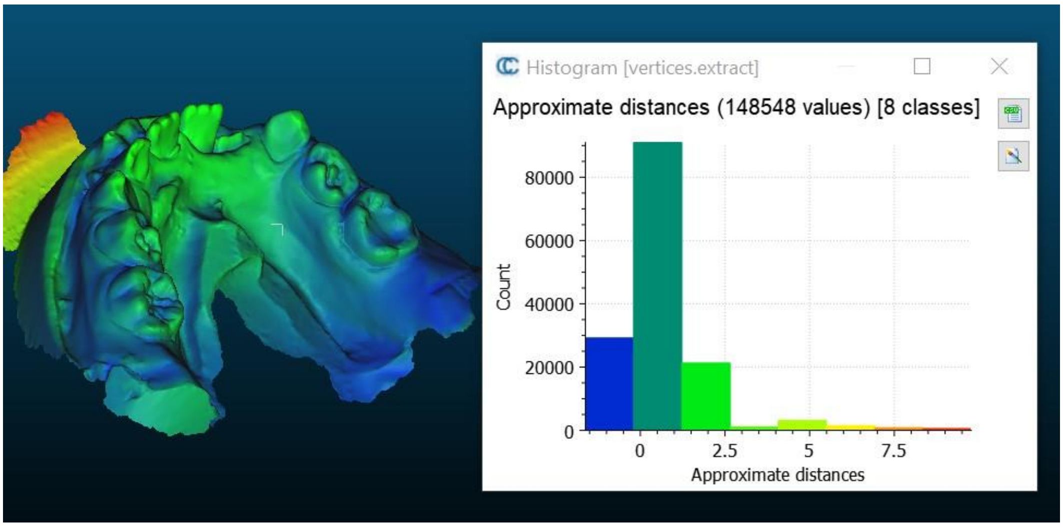

Fig 7: J03 lower jaw .

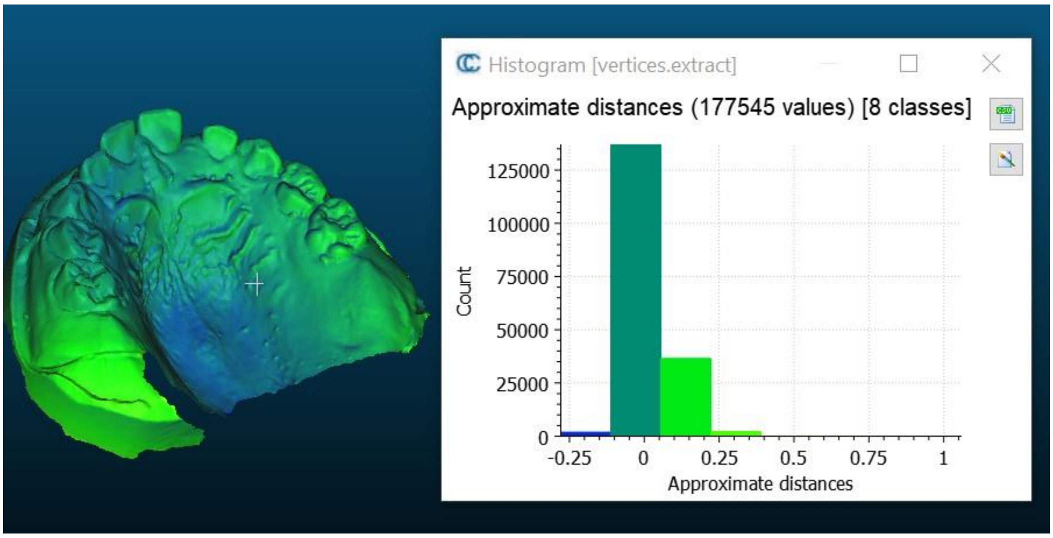

Fig 8: J03 upper jaw .

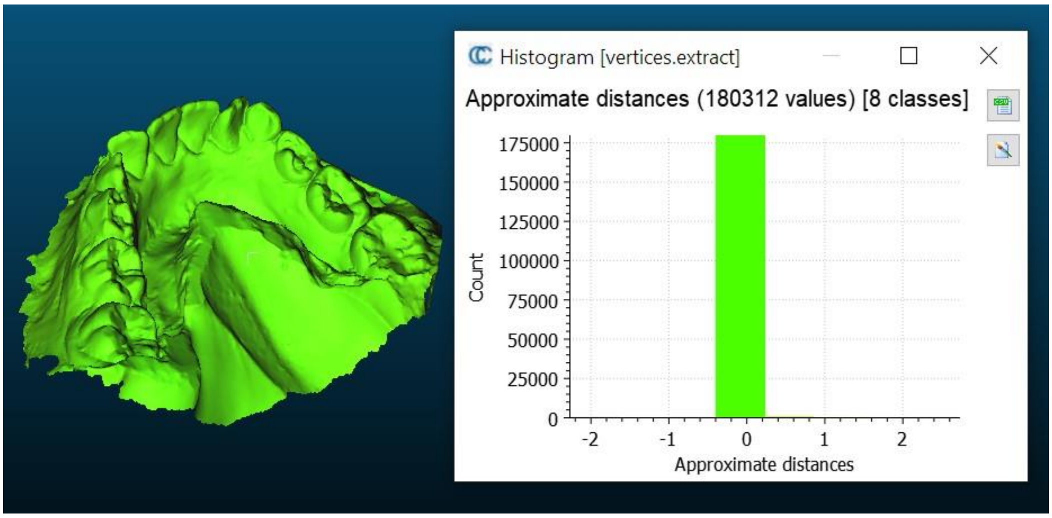

Fig 9: J04 lower jaw .

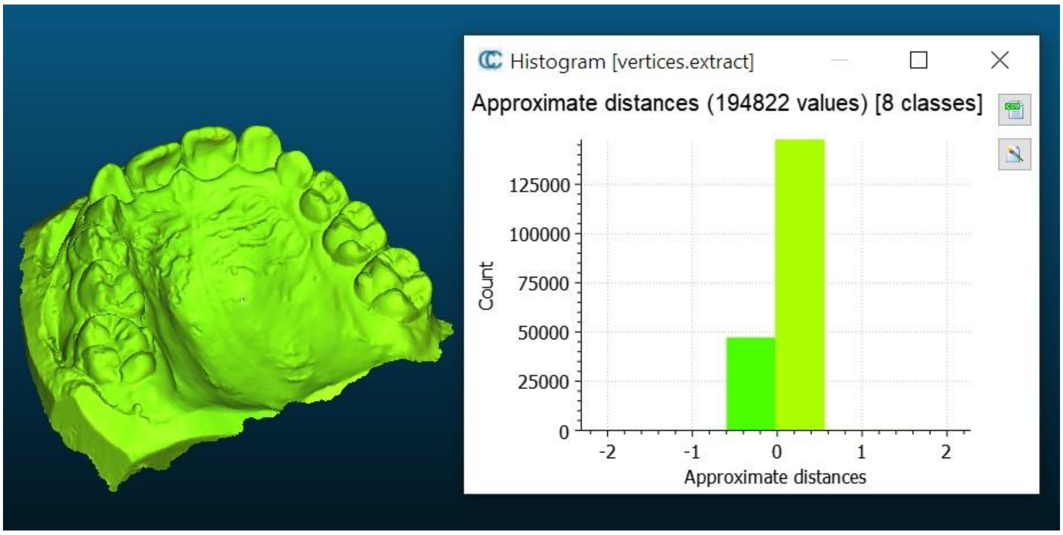

Fig 10: J04 upper jaw .

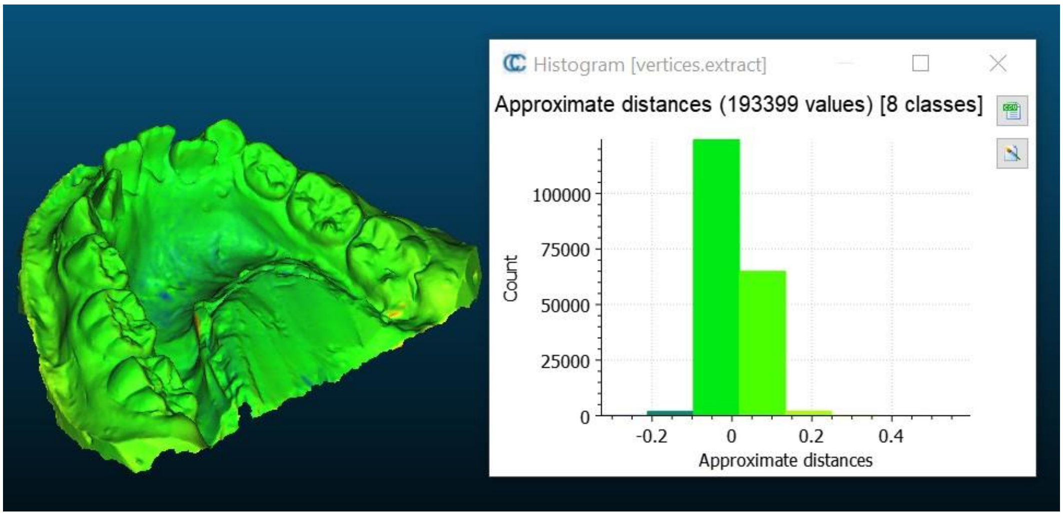

Fig 11: J05 lower jaw .

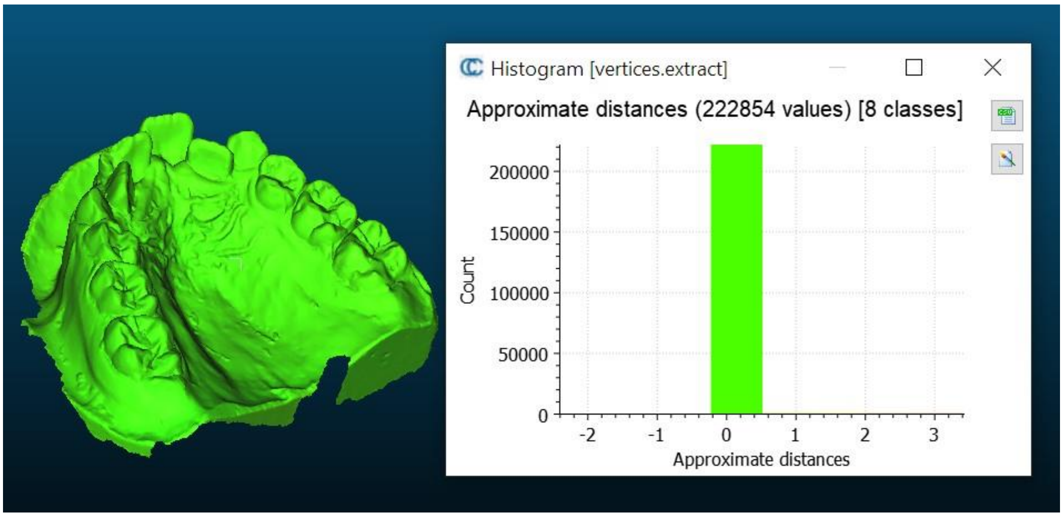

Fig 12: J05 upper jaw .

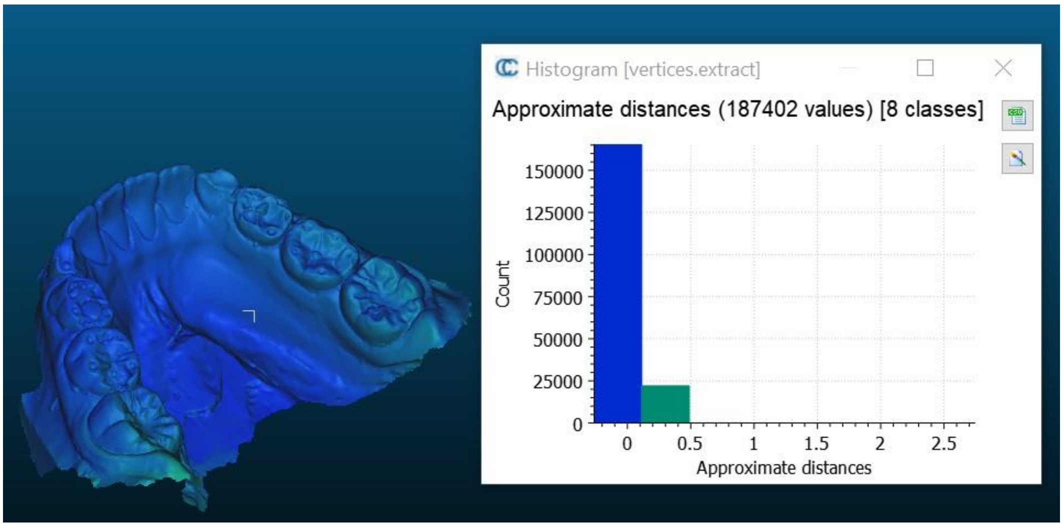

Fig 13: J06 lower jaw .

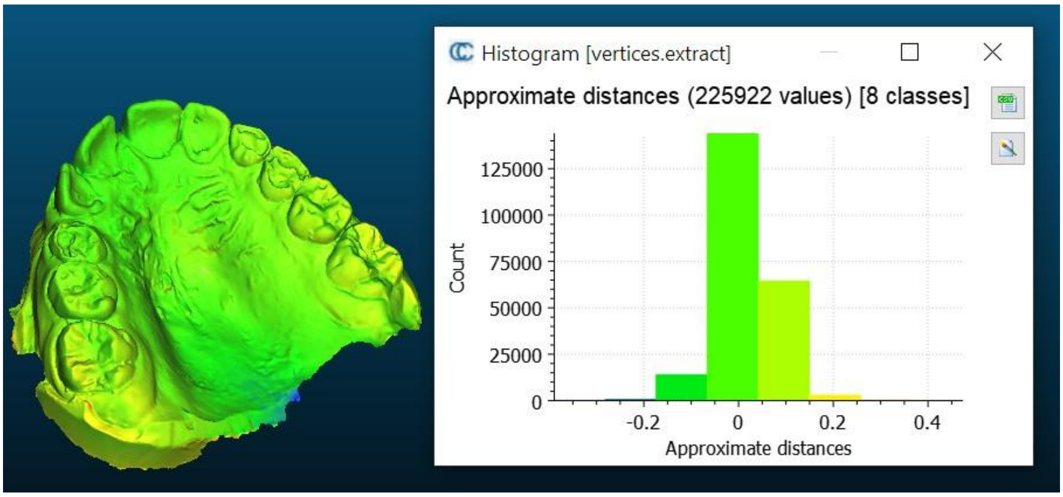

Fig 14: J06 upper jaw .

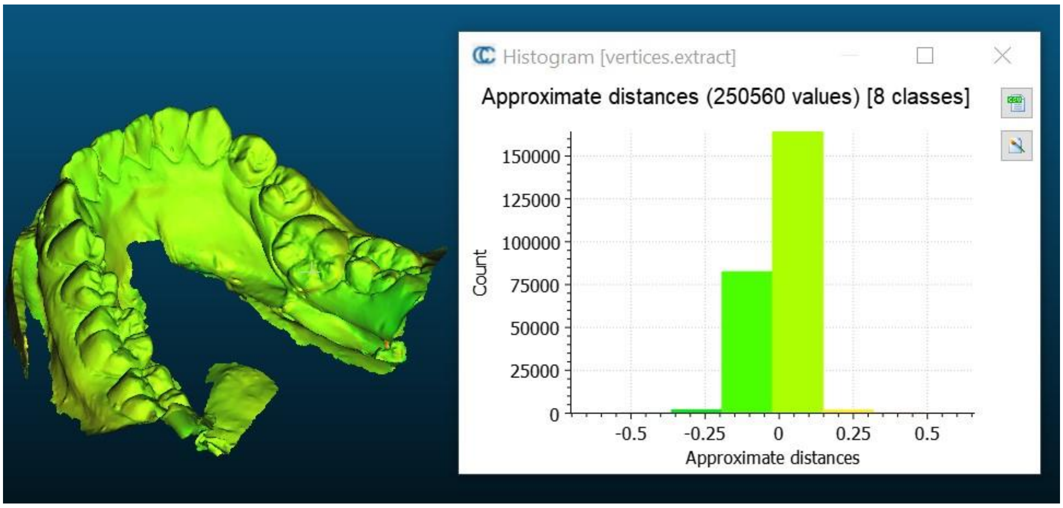

Fig 15: J07 lower jaw .

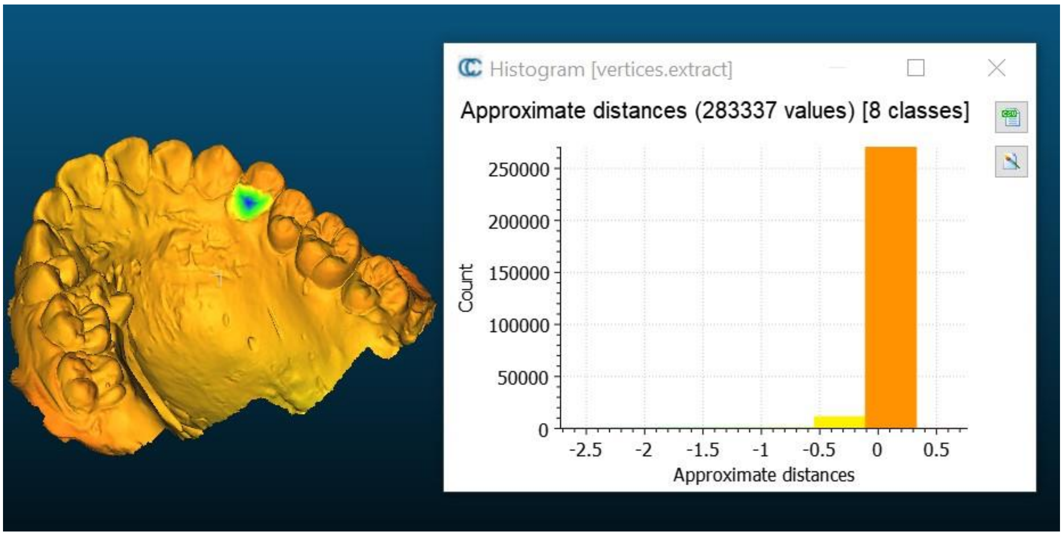

Fig 16: J07 upper jaw .

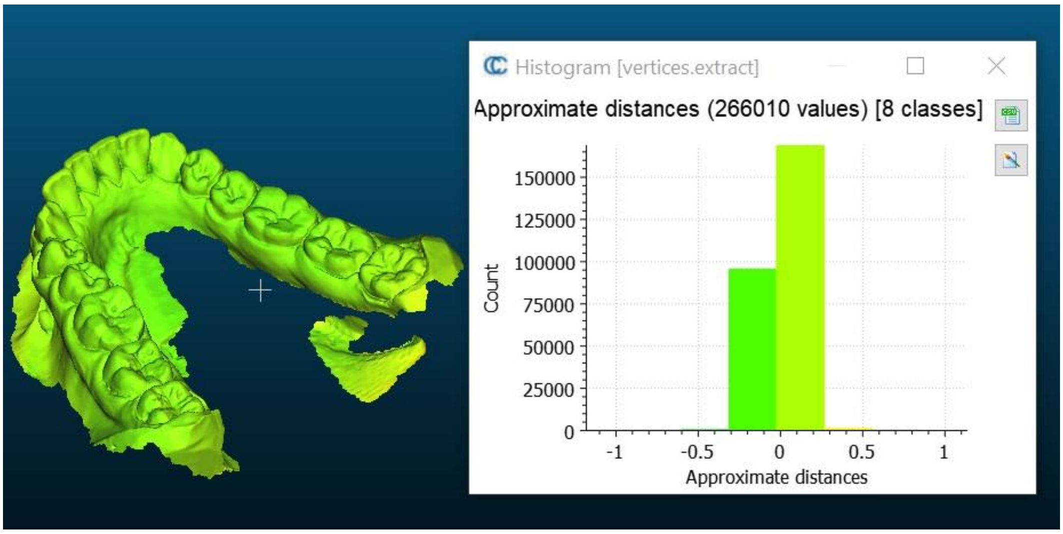

Fig 17: J08 lower jaw .

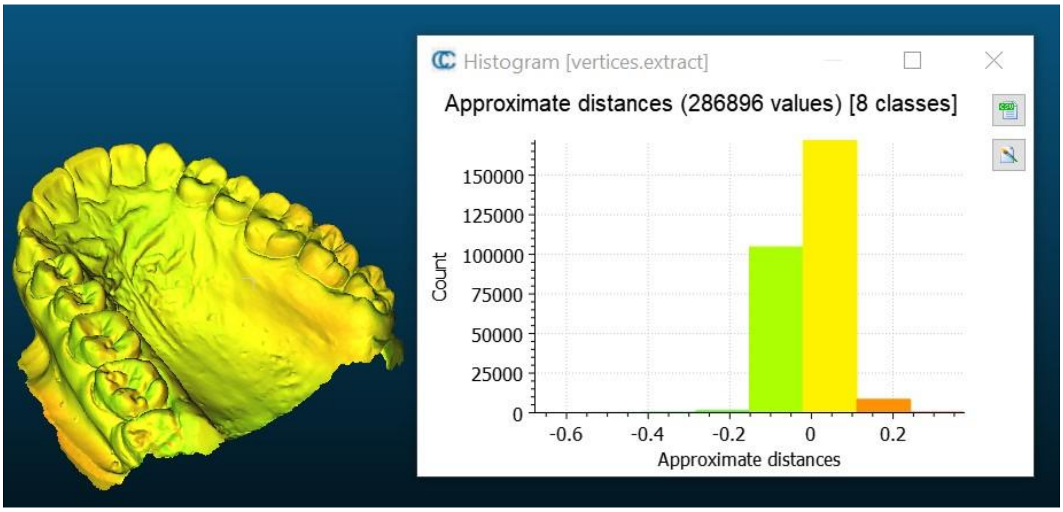

Fig 18: J08 upper jaw .

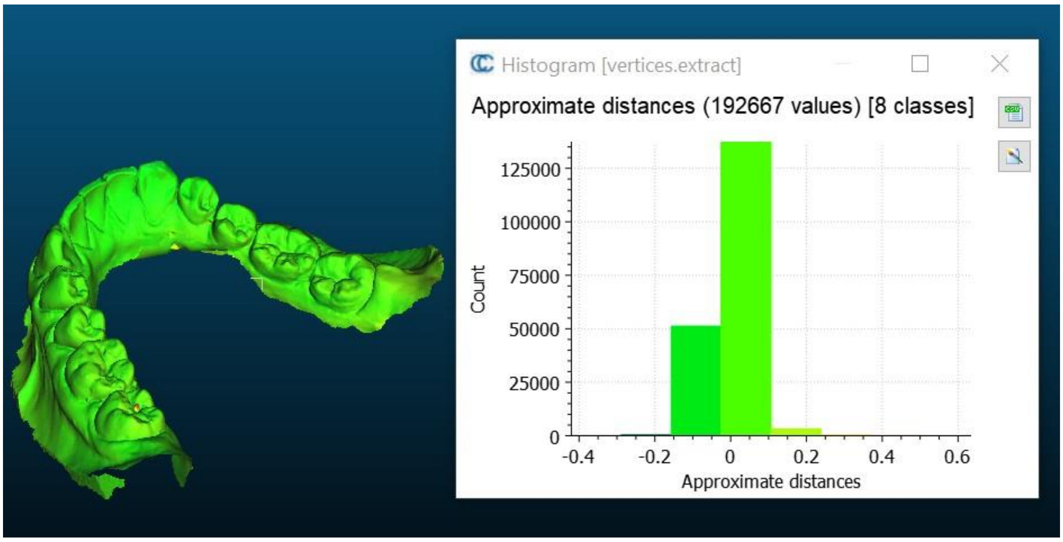

Fig 19: J09 lower jaw .

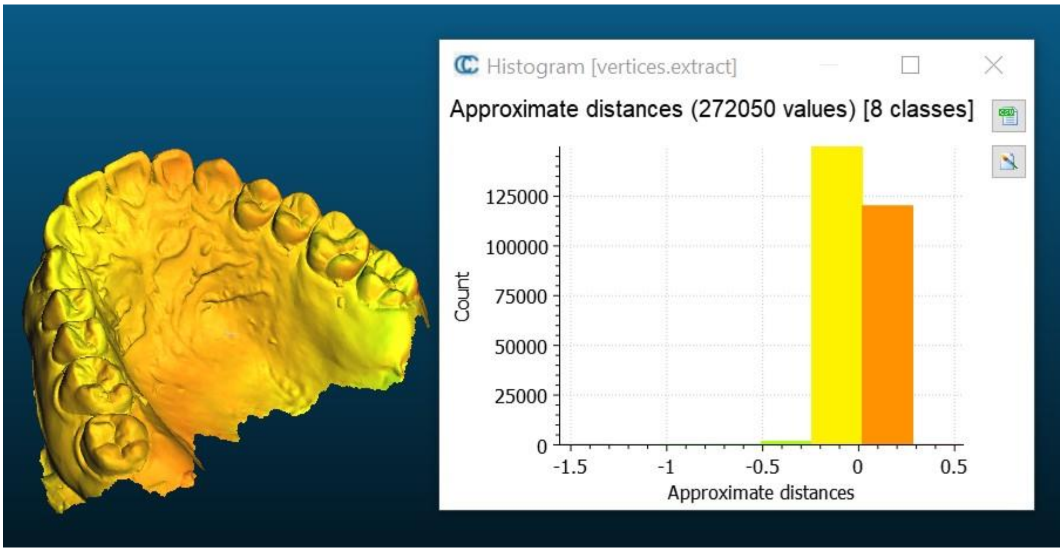

Fig 20: J09 upper jaw .

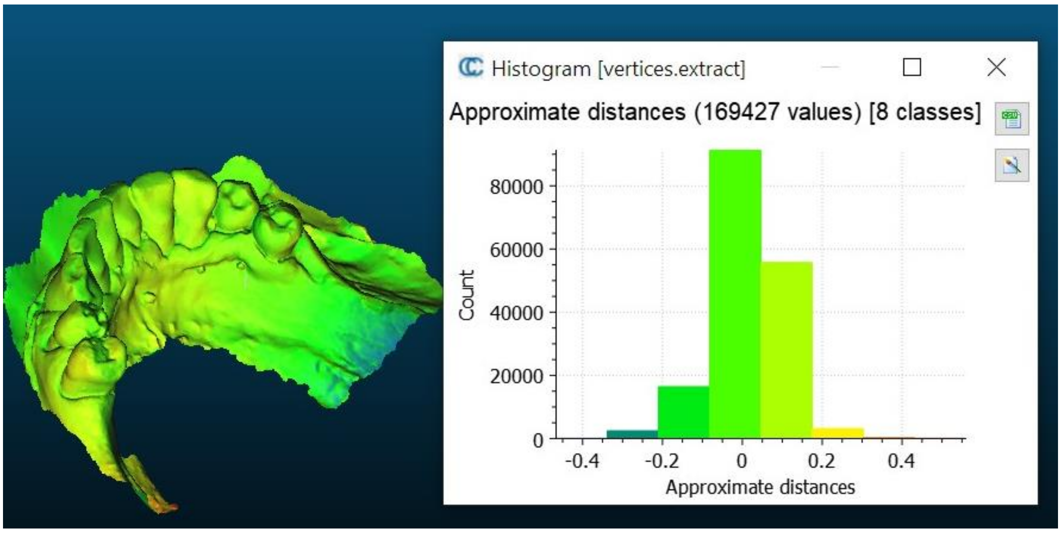

Fig 21: J10 lower jaw .

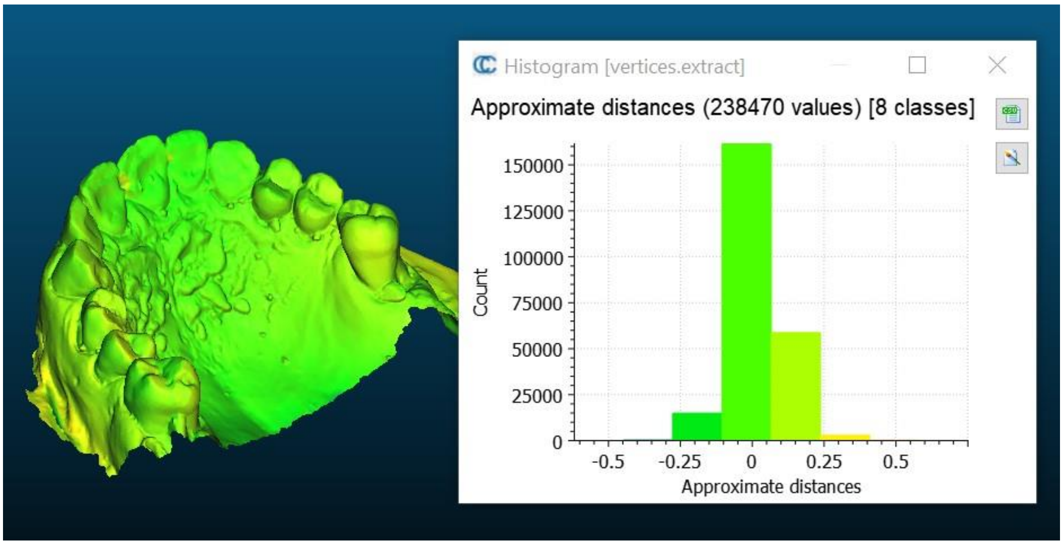

Fig 22: J10 upper jaw .

IOS2 + OP2

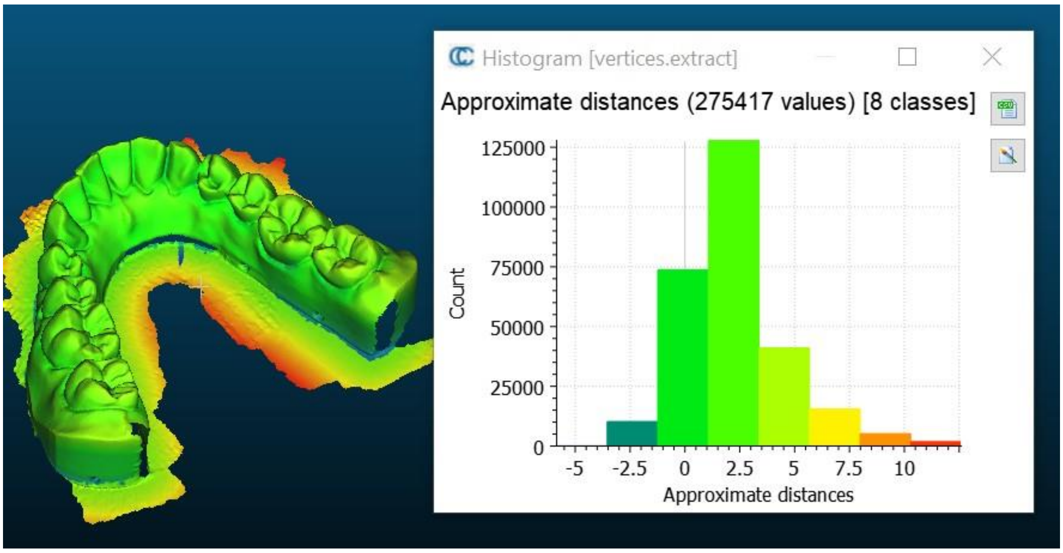

Fig 1: T standard model lower jaw .

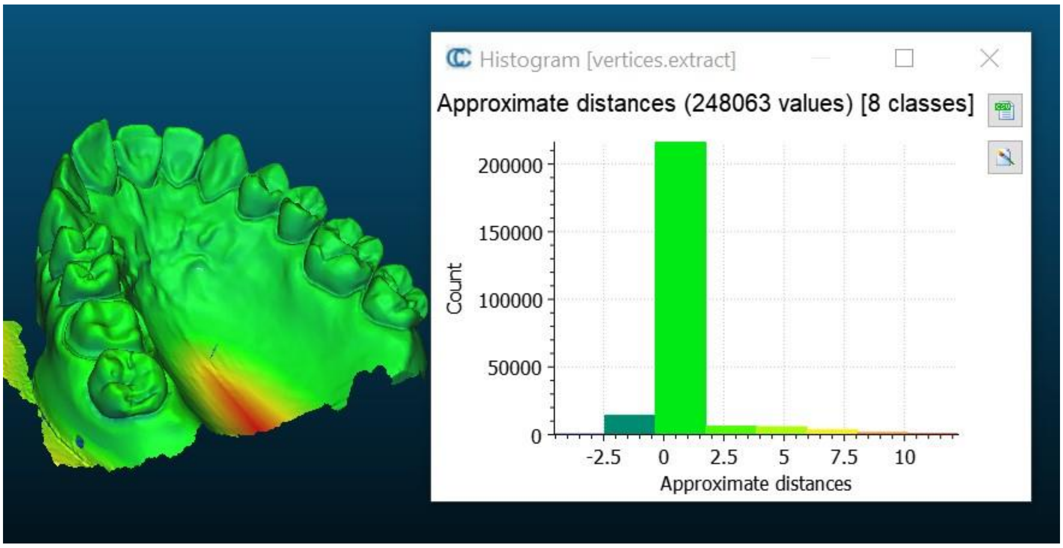

Fig 2: T standard model upper jaw .

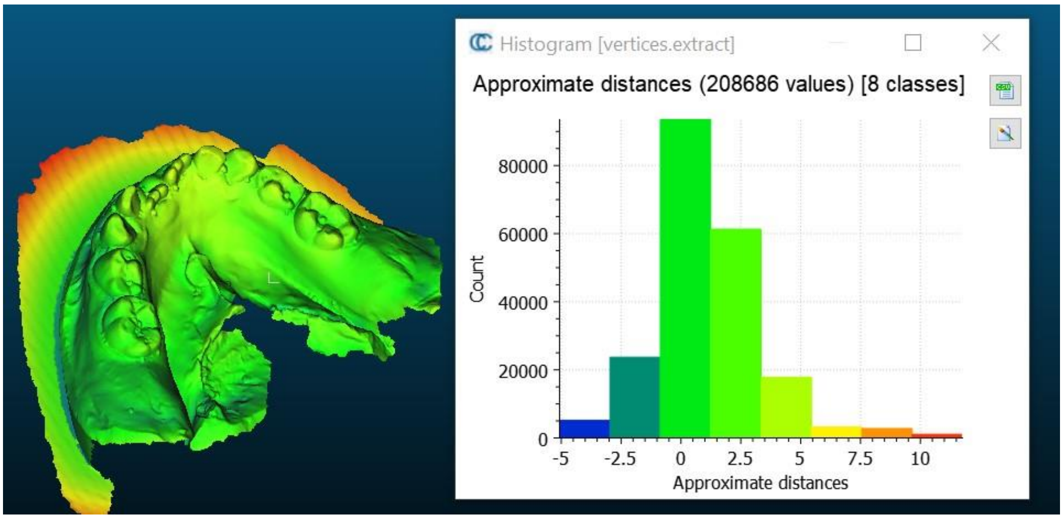

Fig 3: T01 lower jaw .

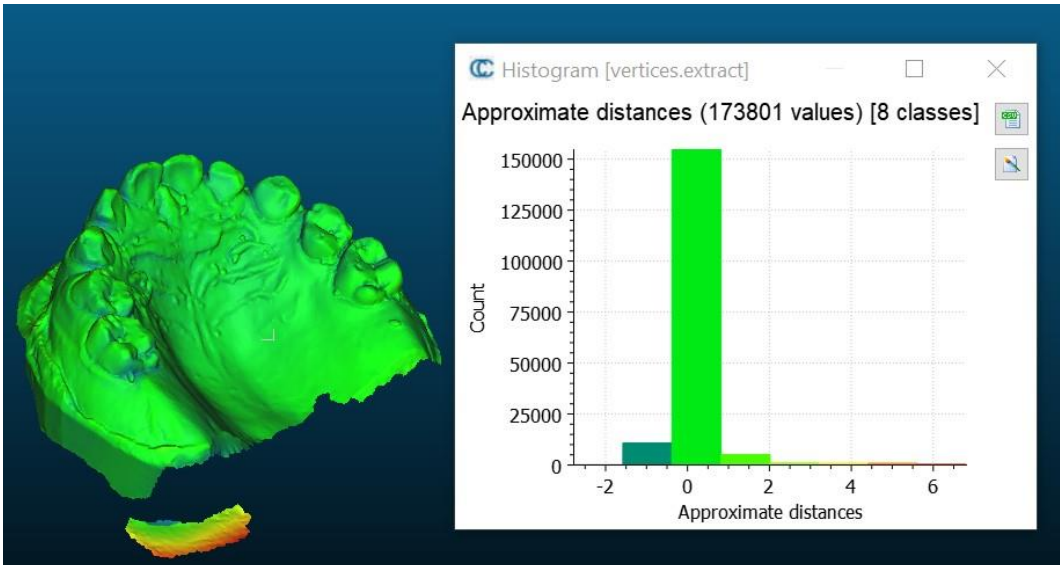

Fig 4: T01 upper jaw .

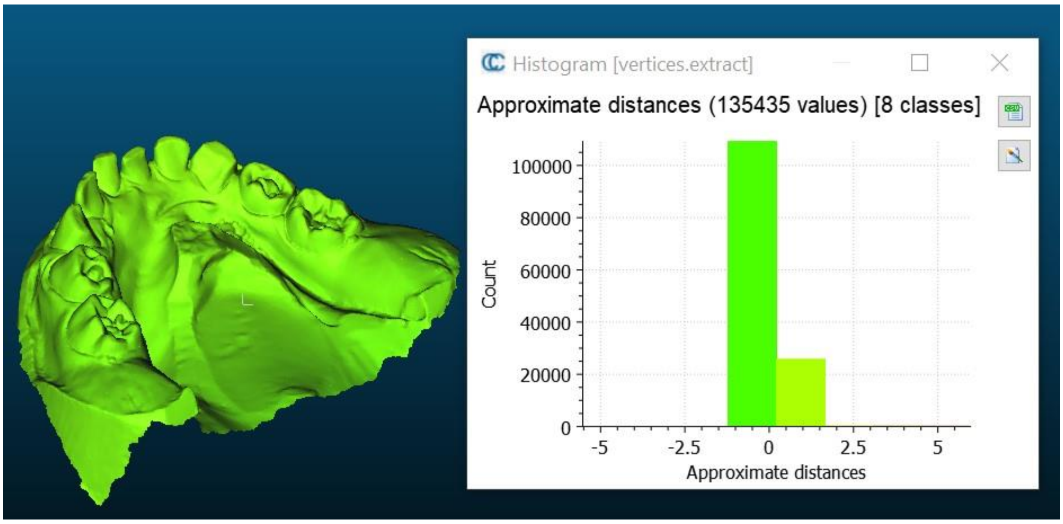

Fig 5: T02 lower jaw .

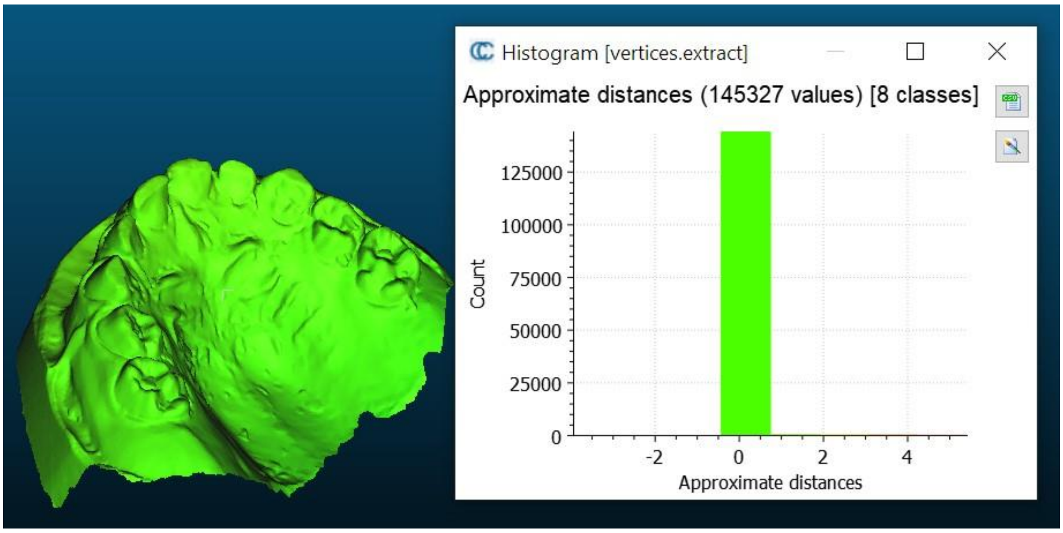

Fig 6: T02 upper jaw .

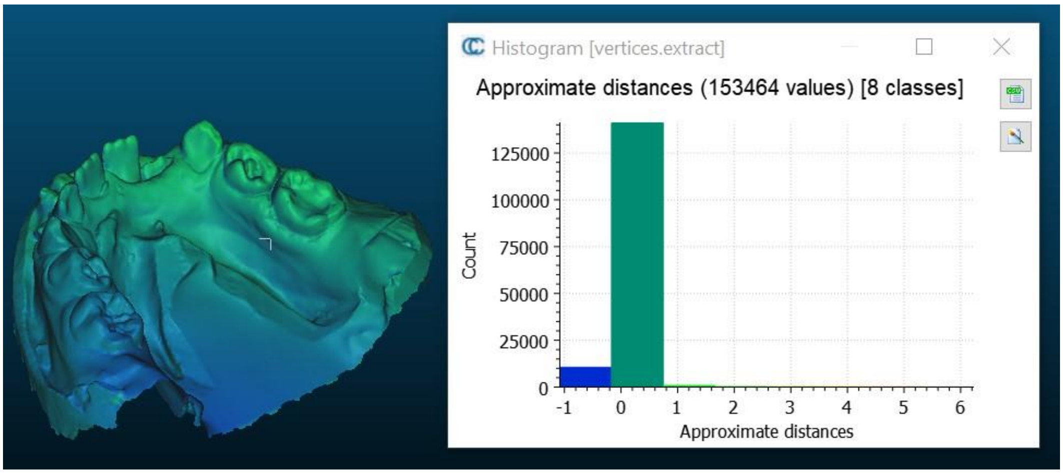

Fig 7: T03 lower jaw .

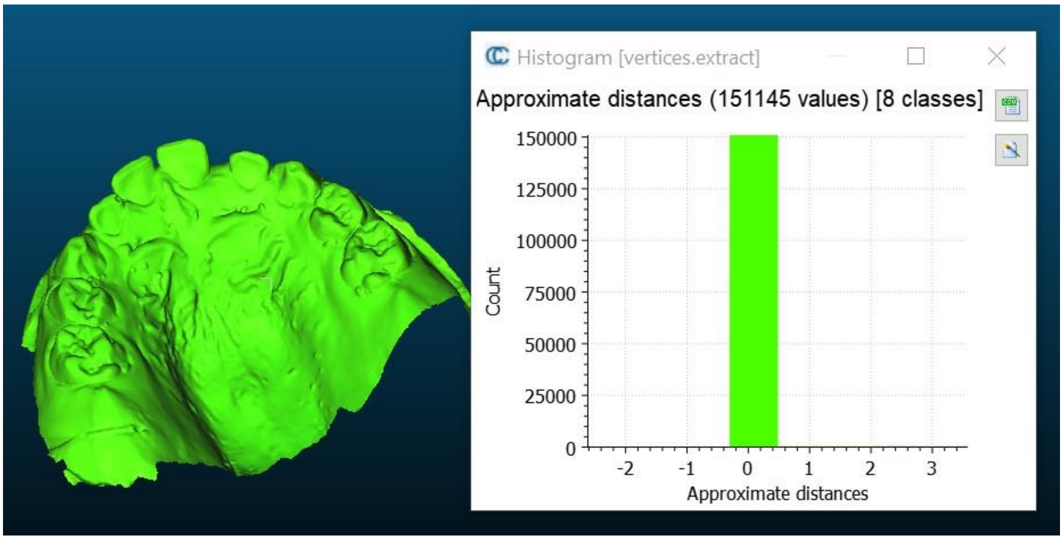

Fig 8: T03 upper jaw .

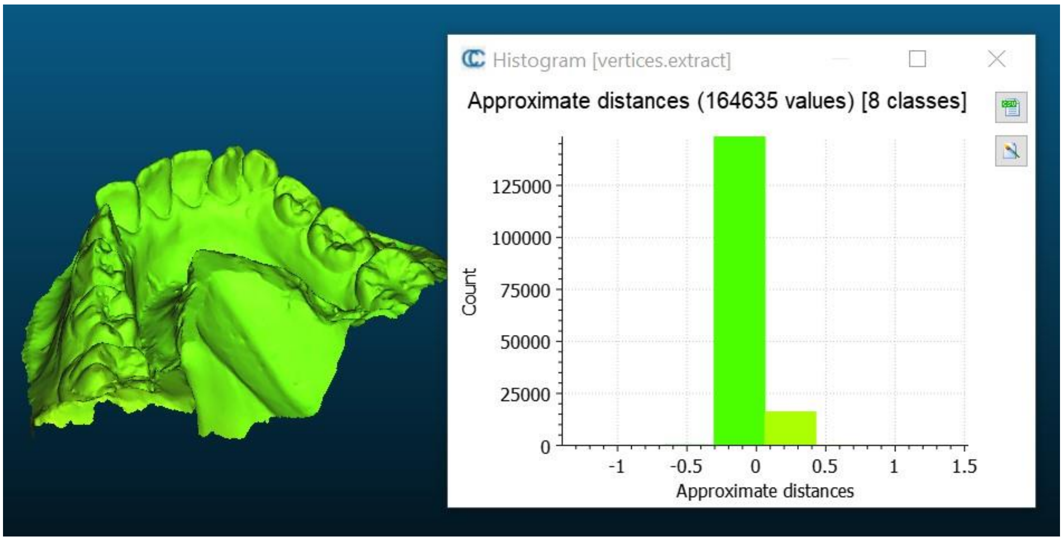

Fig 9: T04 lower jaw .

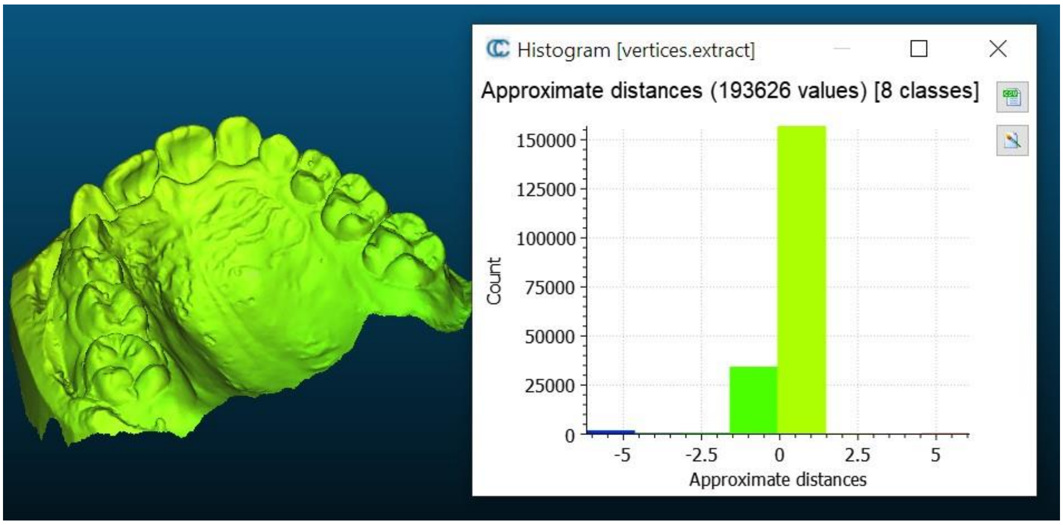

Fig 10: T04 upper jaw .

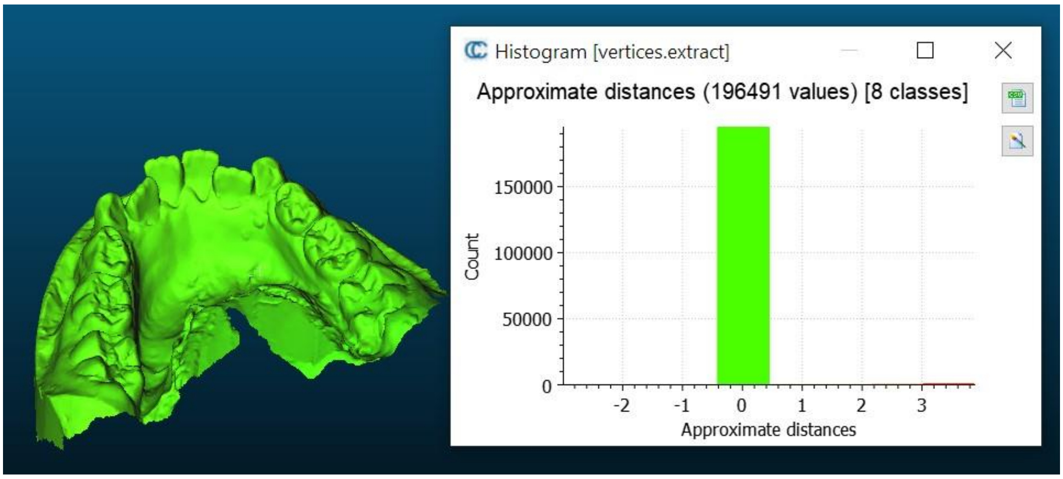

Fig 11: T05 lower jaw .

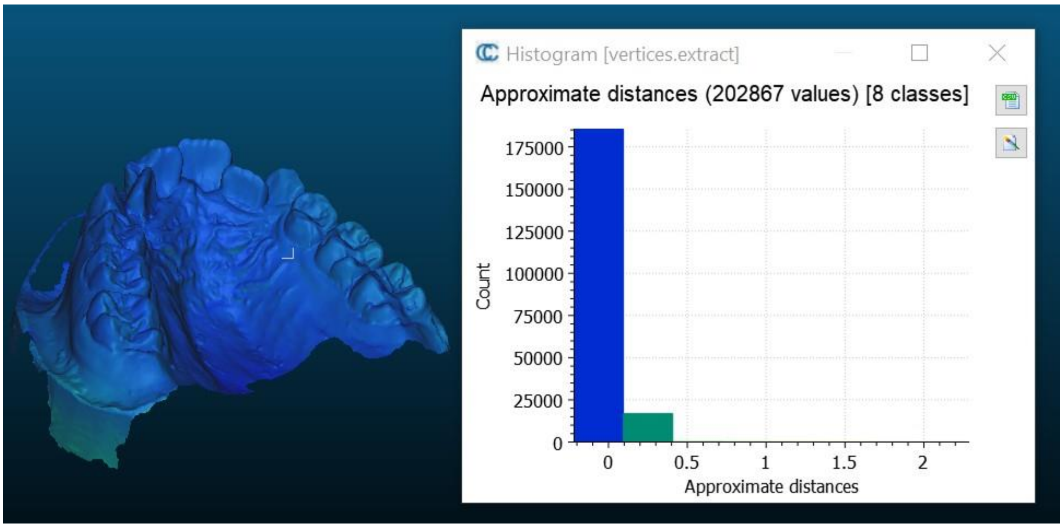

Fig 12: T05 upper jaw .

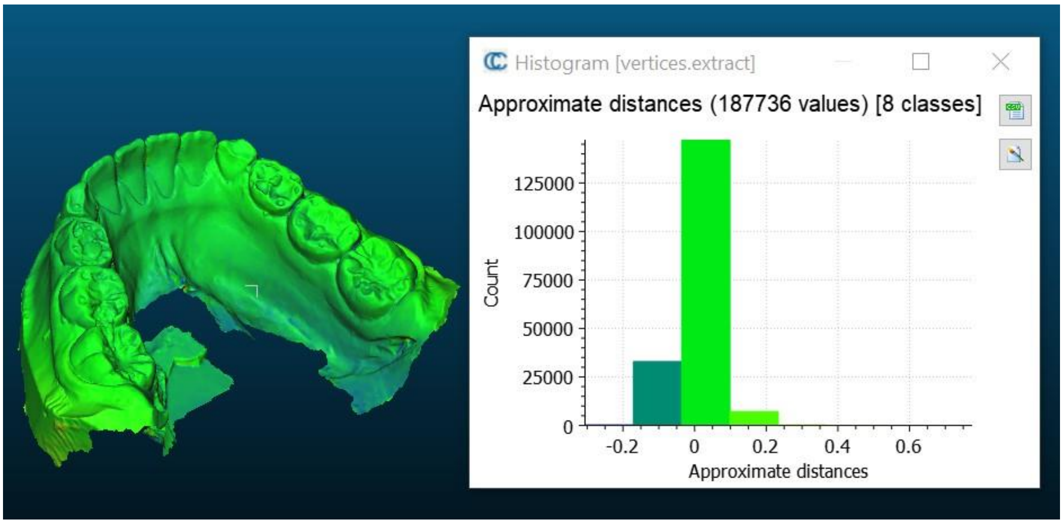

Fig 13: T06 lower jaw .

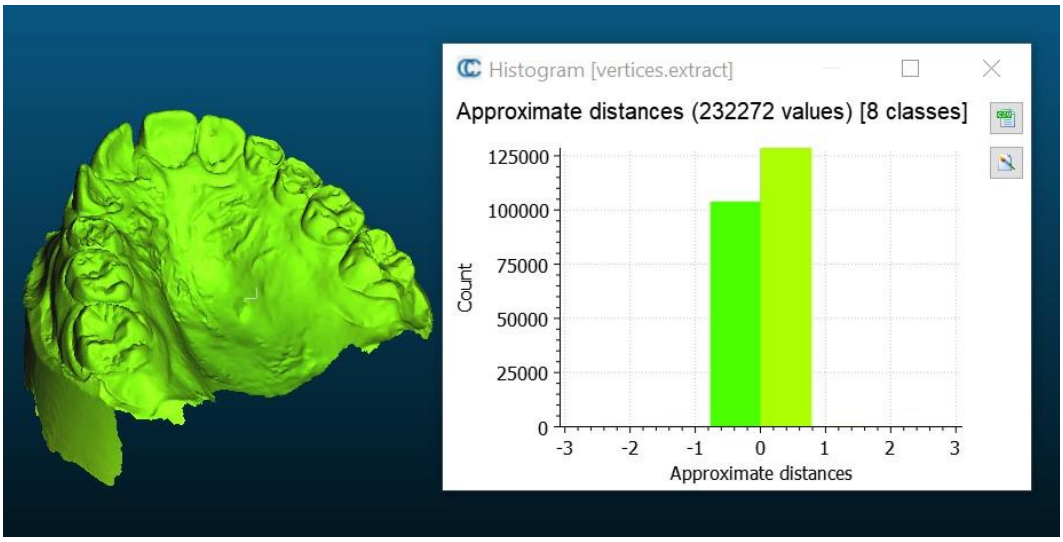

Fig 14: T06 upper jaw .

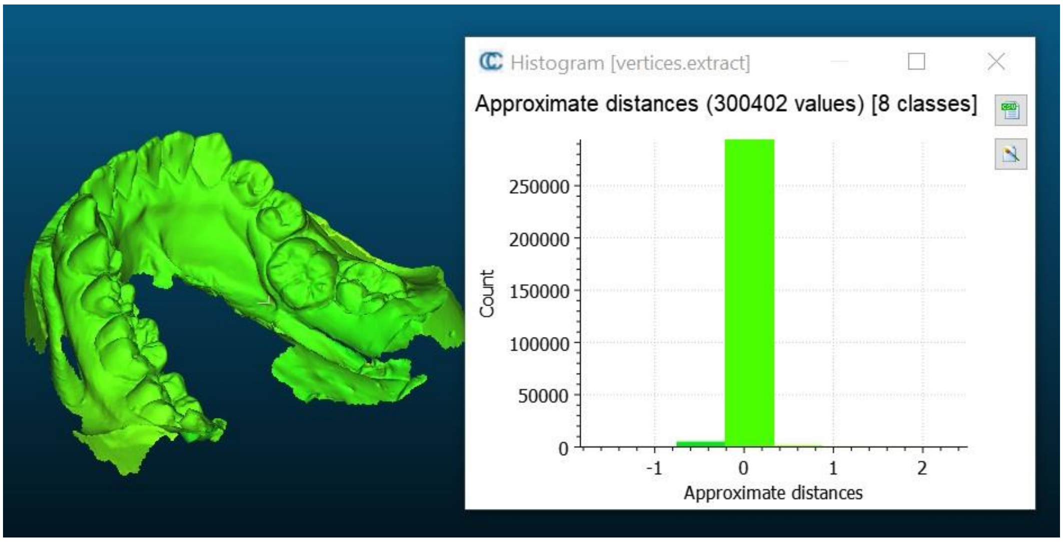

Fig 15: T07 lower Jaw .

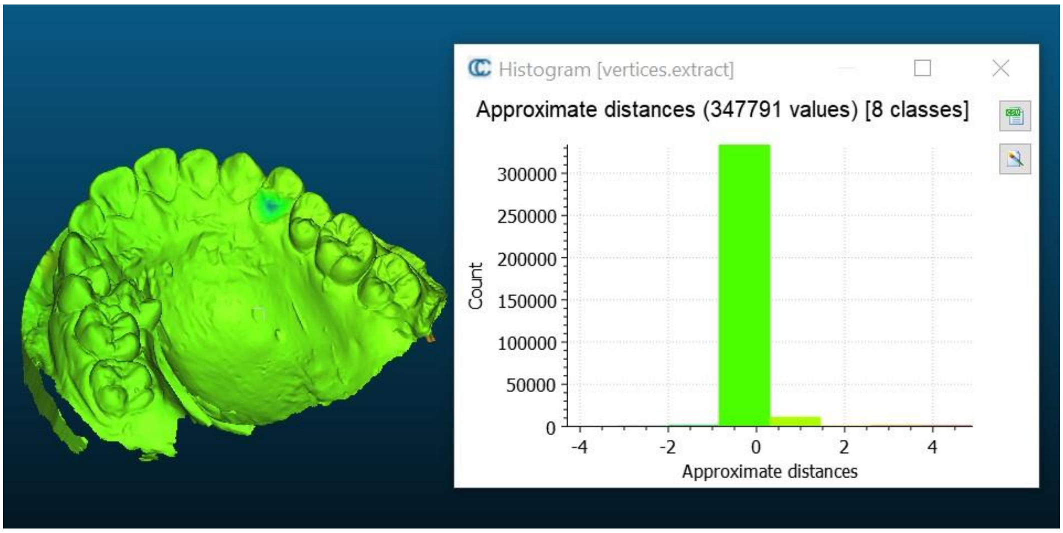

Fig 16: T07 upper Jaw .

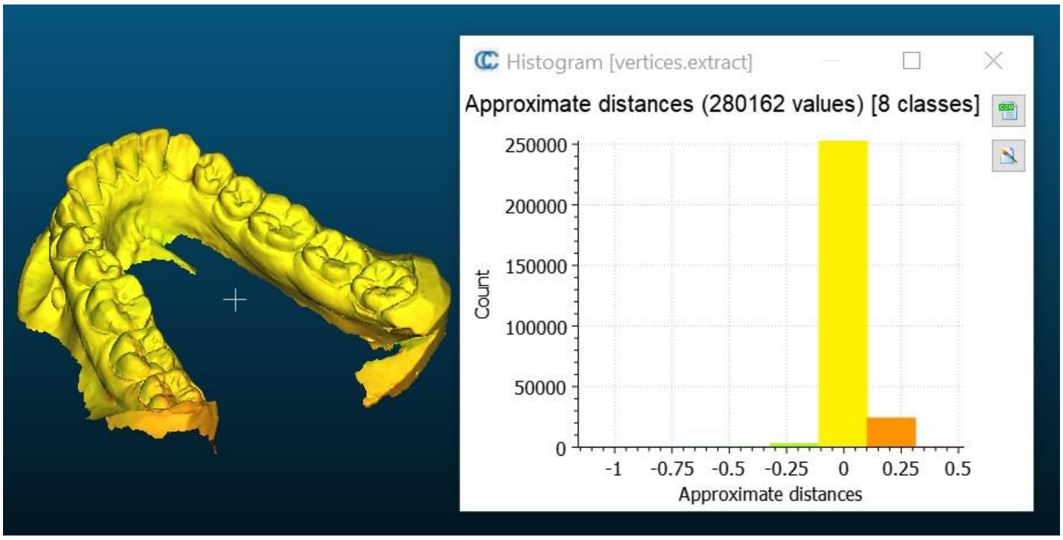

Fig 17: T08 lower jaw .

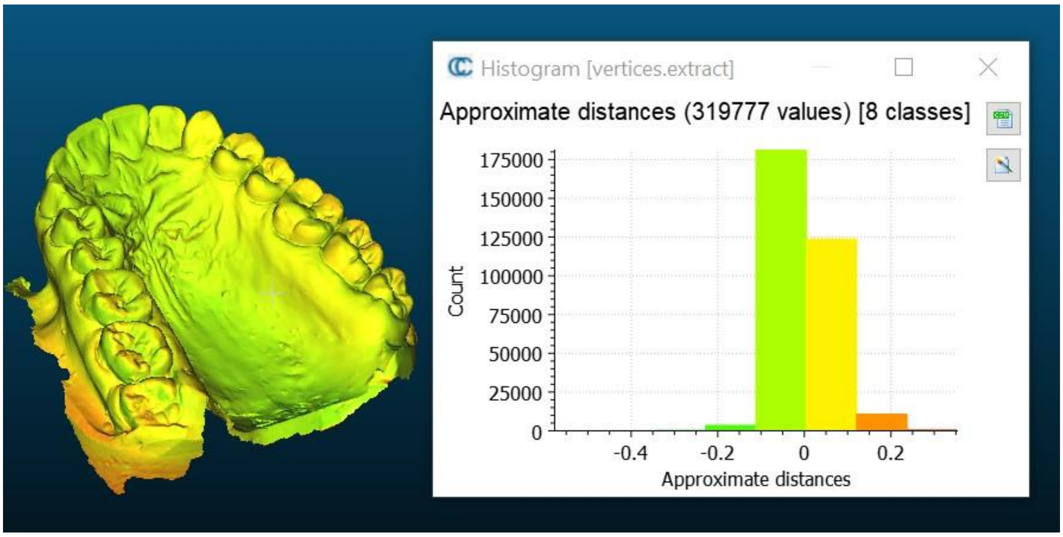

Fig 18: T08 upper Jaw .

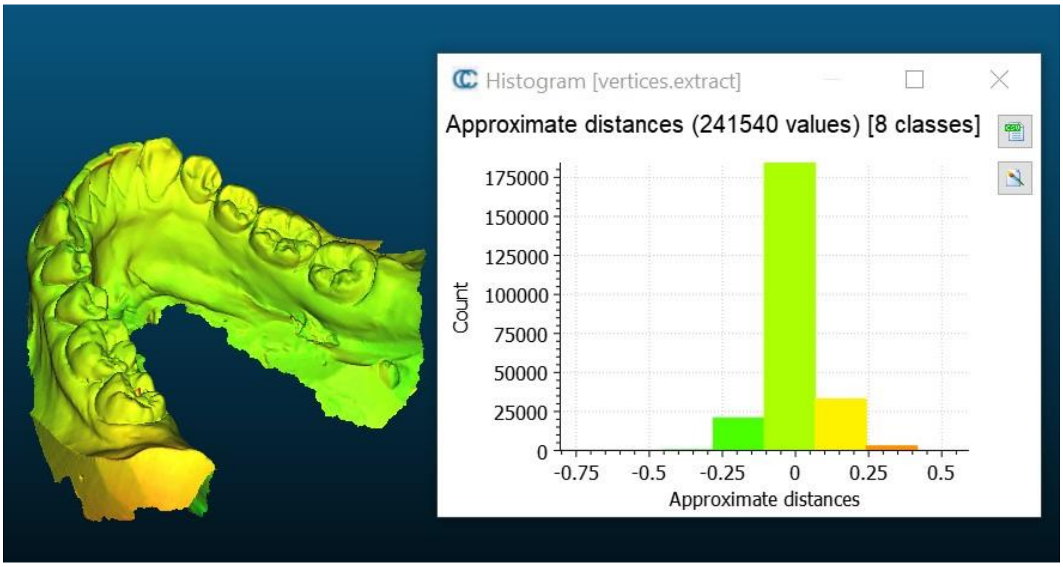

Fig 19: T09 lower Jaw .

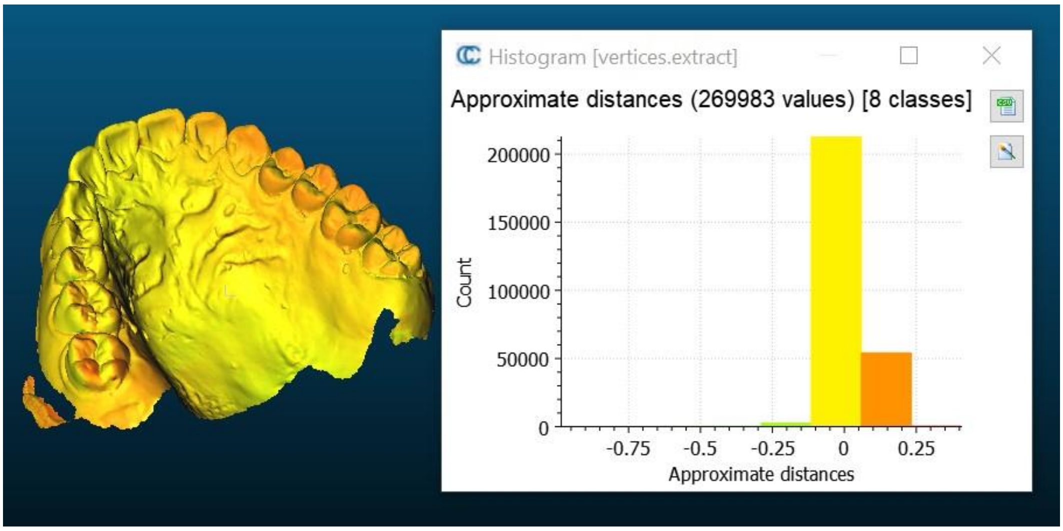

Fig 20: T09 upper jaw .

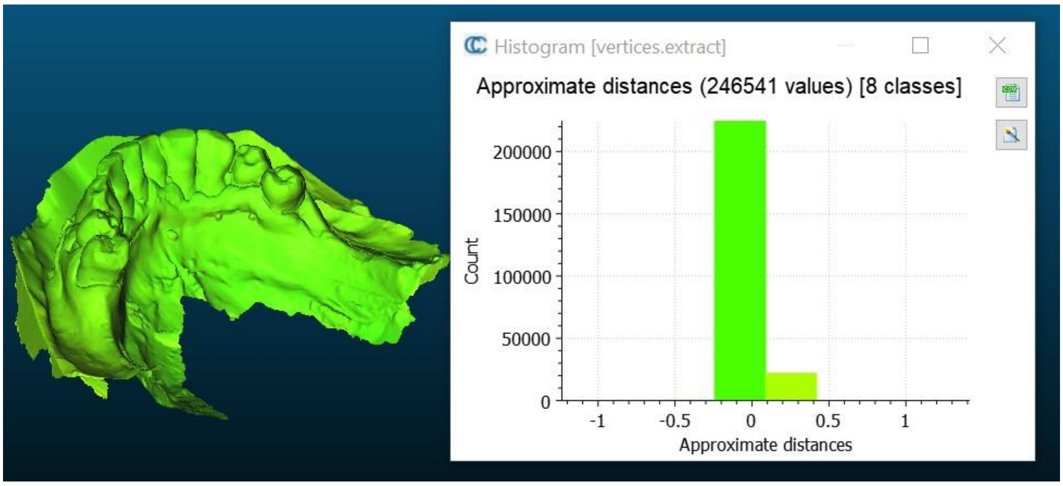

Fig 21: T10 lower jaw .

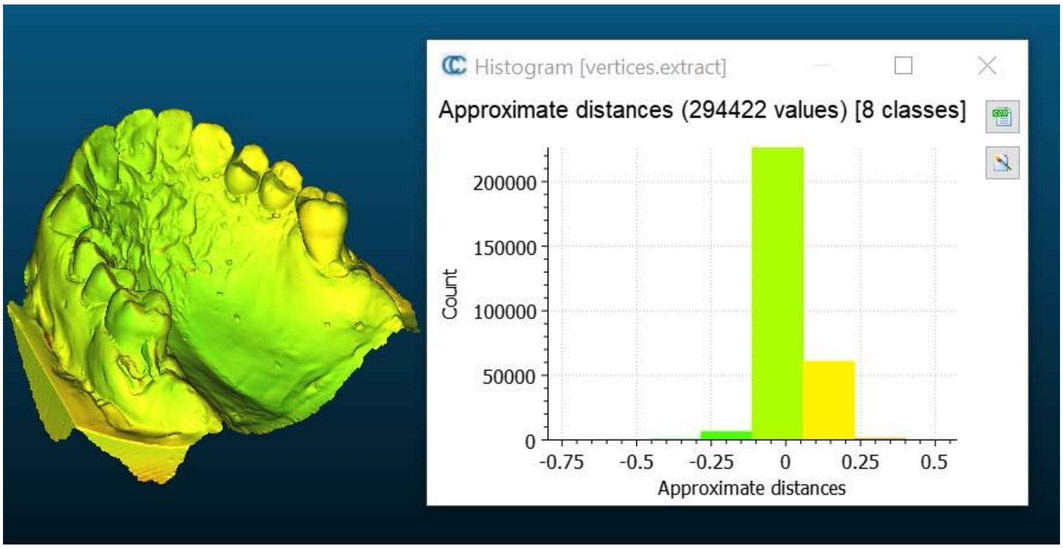

Fig 22: T10 upper jaw .

Heatmaps of Augmented scans generated through Hausdorff's distance evaluation

IOS1 + OP1

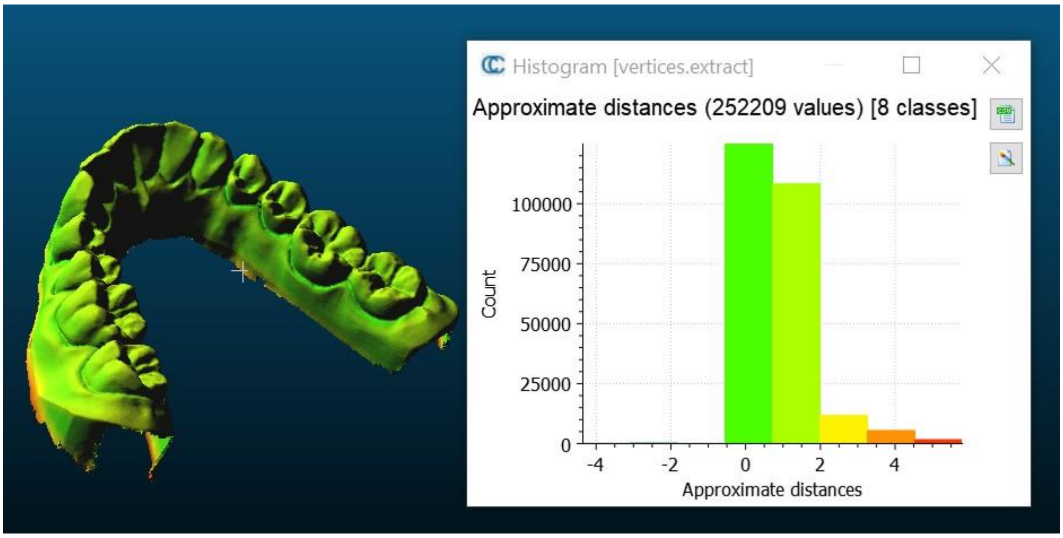

Fig 1: J standard model lower jaw .

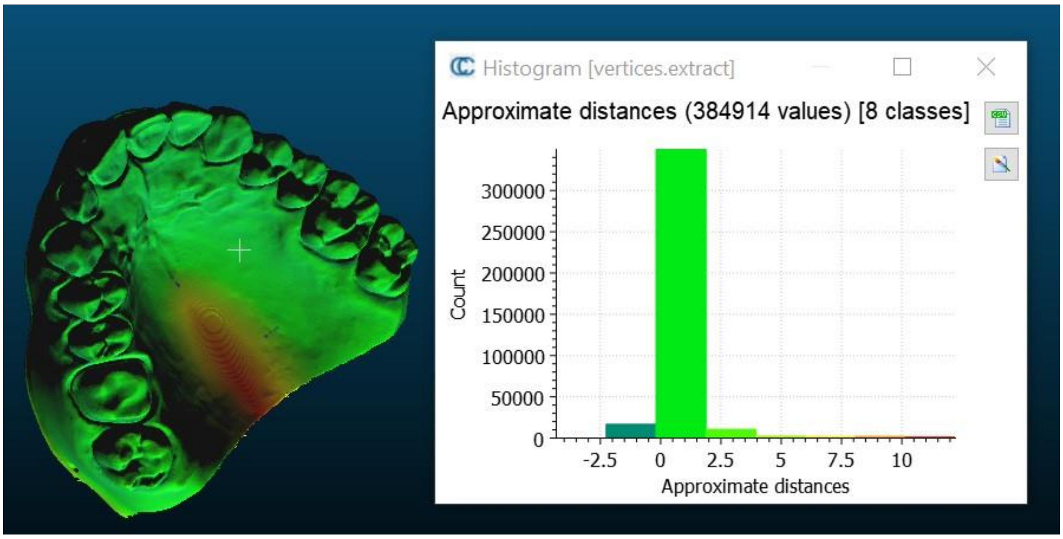

Fig 2: J standard model upper jaw .

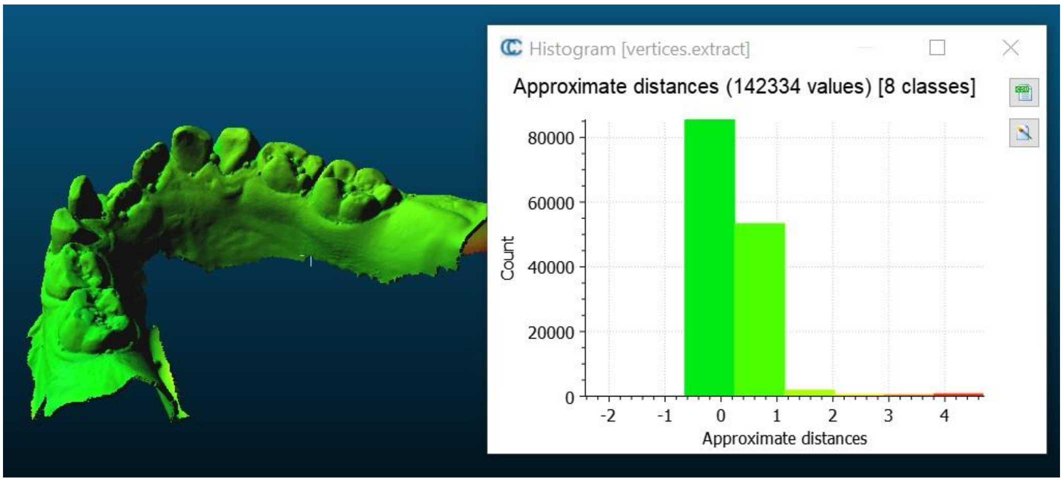

Fig 3: J01 lower jaw .

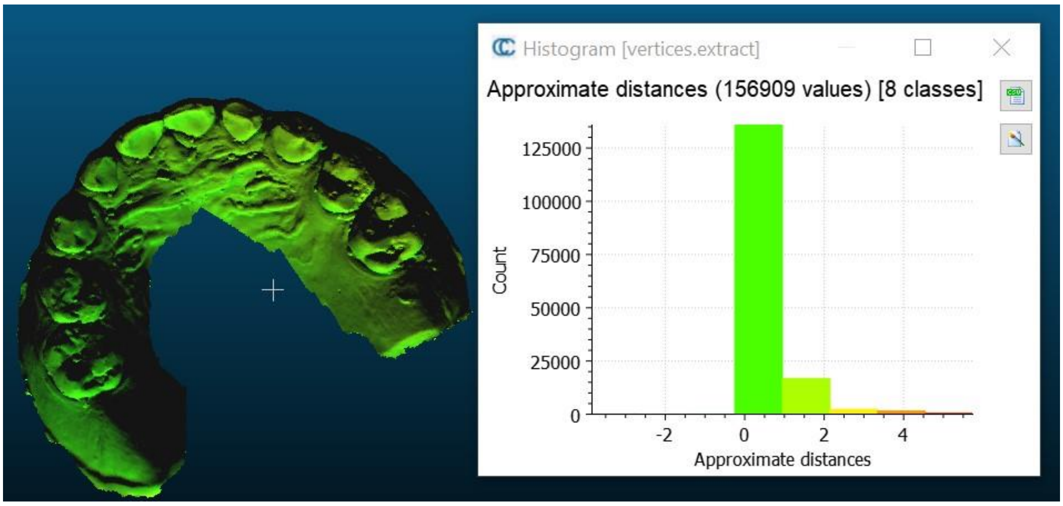

Fig 4: J01 upper jaw .

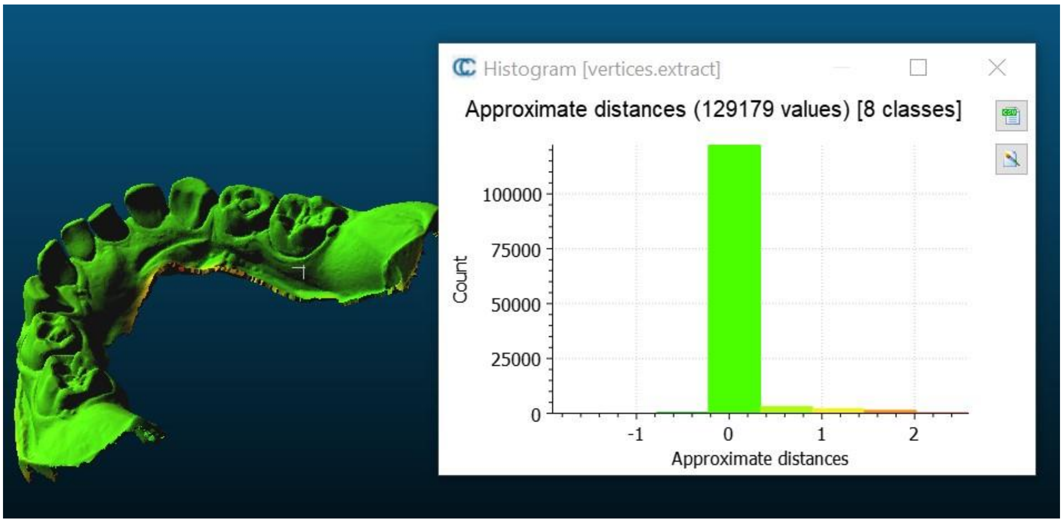

Fig 5: J02 lower jaw .

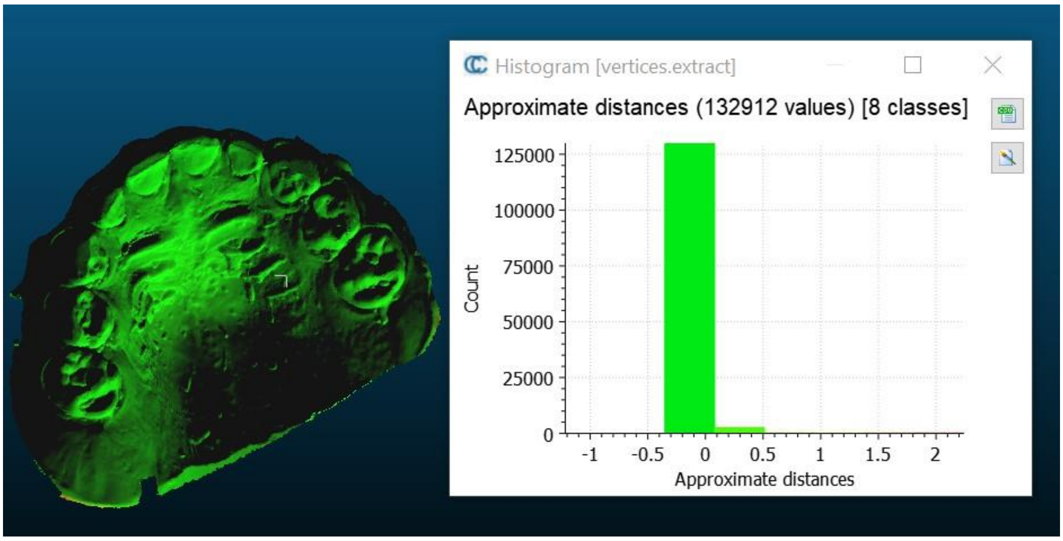

Fig 6: J02 upper jaw .

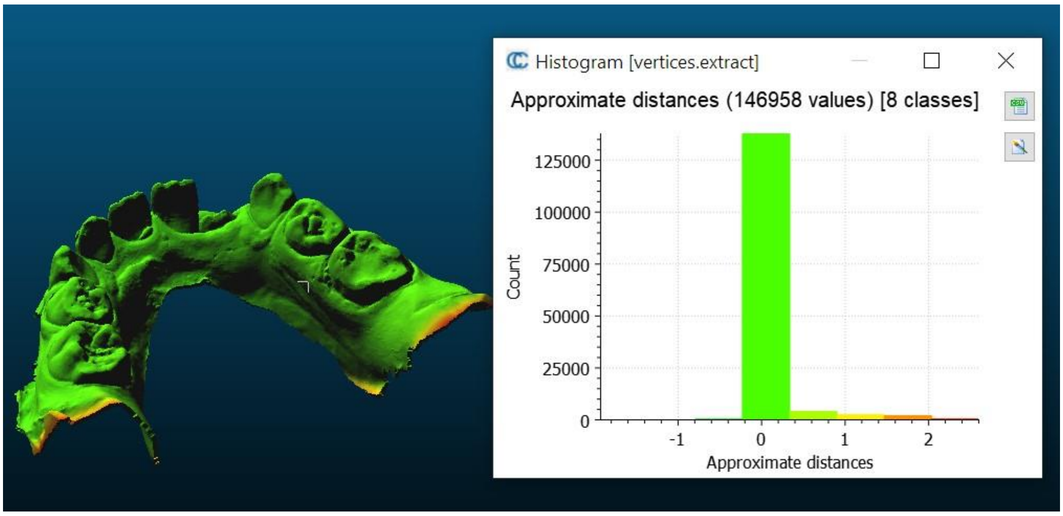

Fig 7: J03 lower jaw .

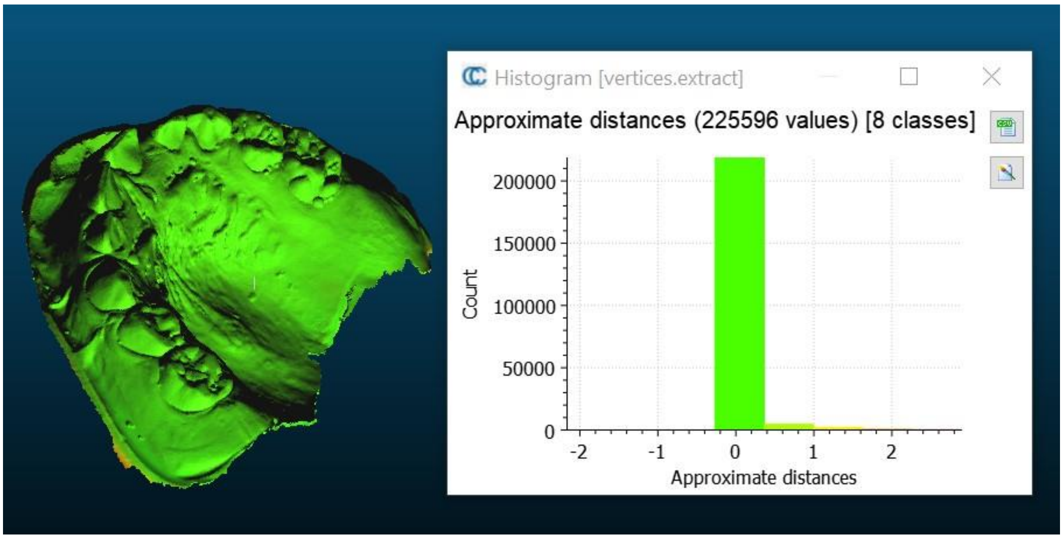

Fig 8: J03 upper jaw .

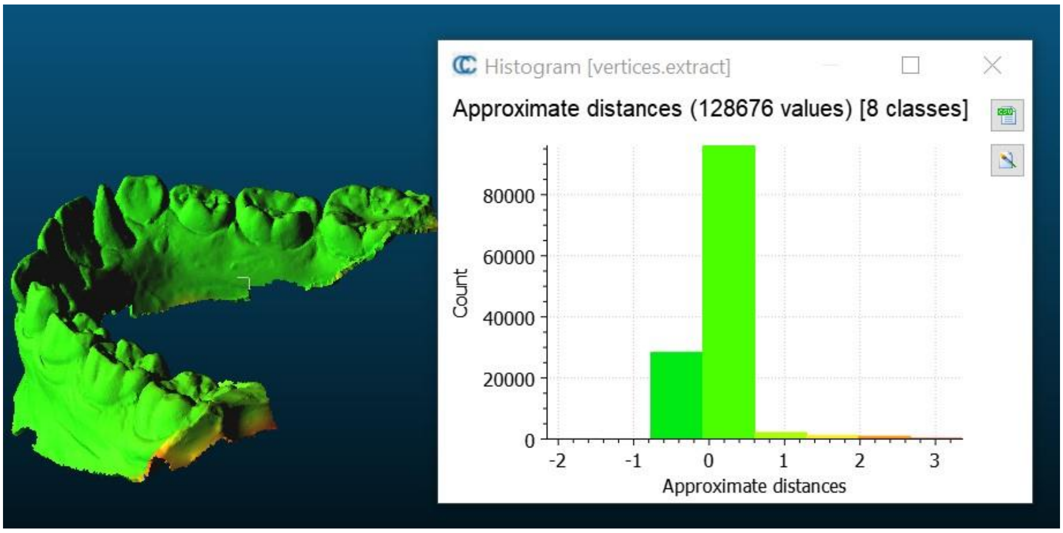

Fig 9: J04 lower jaw .

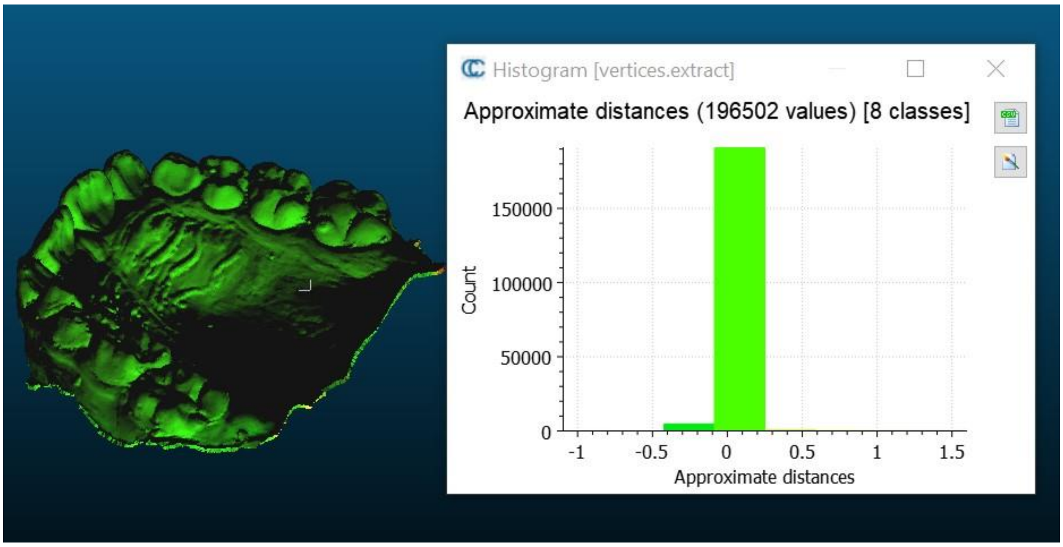

Fig 10: J04 upper jaw .

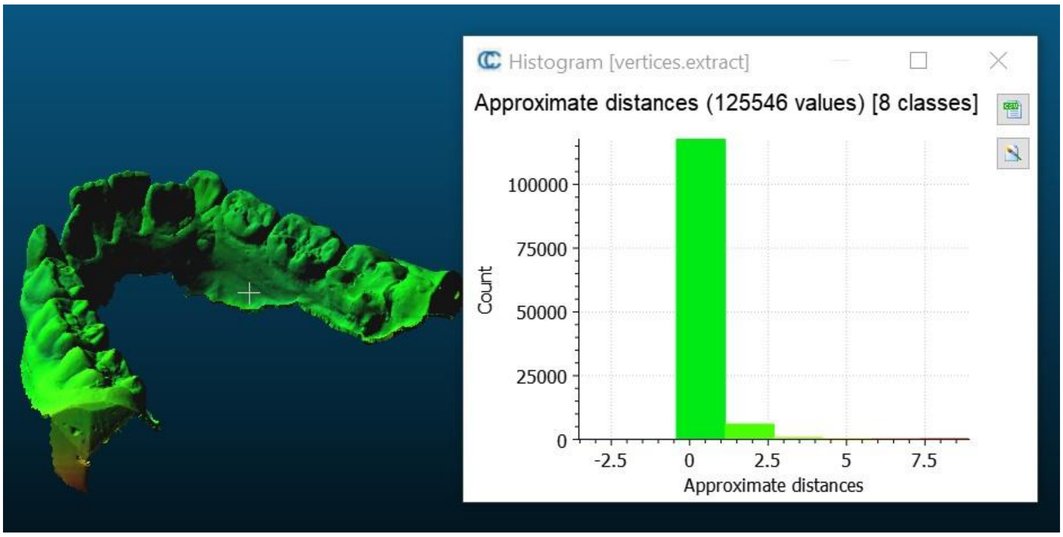

Fig 11: J05 lower jaw .

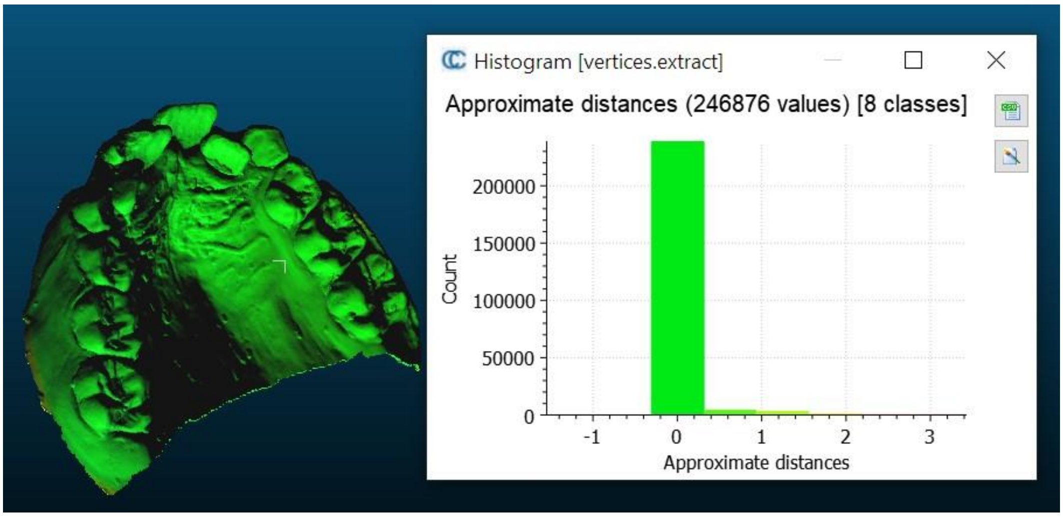

Fig 12: J05 upper jaw .

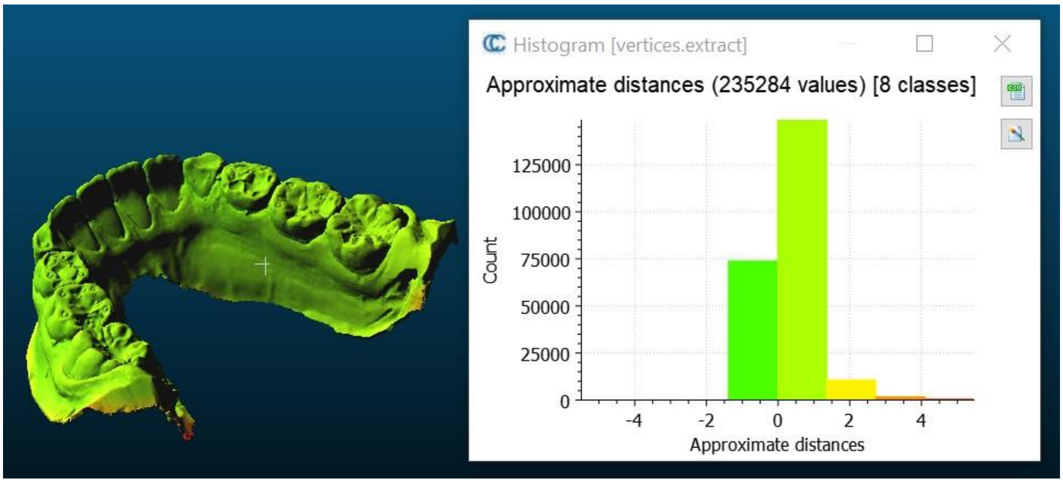

Fig 13: J06 lower jaw .

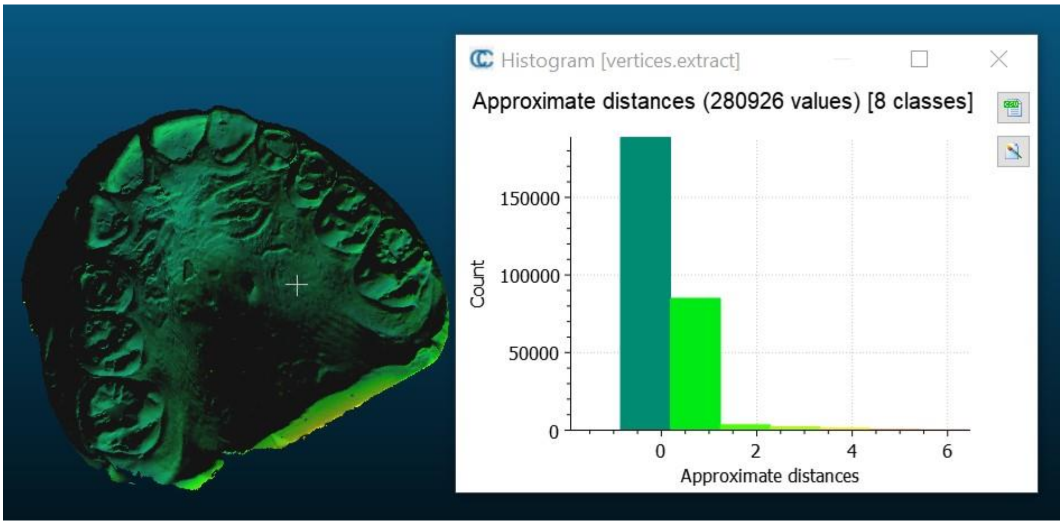

Fig 14: J06 upper jaw .

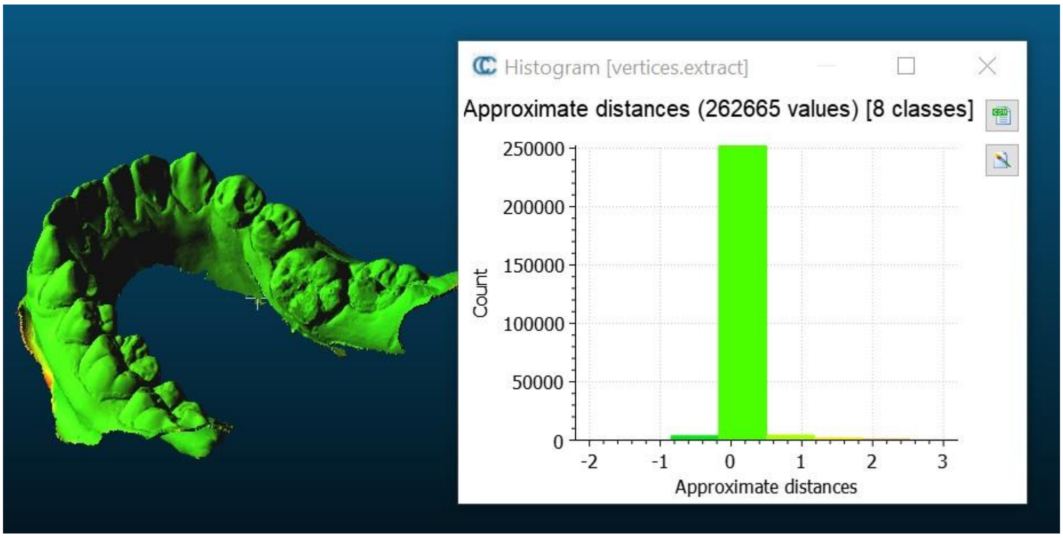

Fig 15: J07 lower jaw .

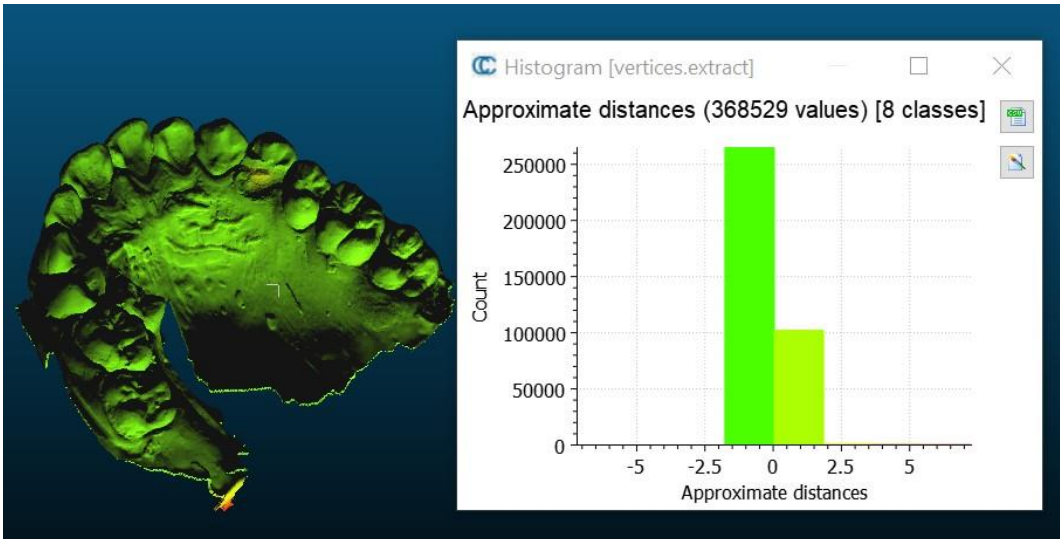

Fig 16: J07 upper jaw .

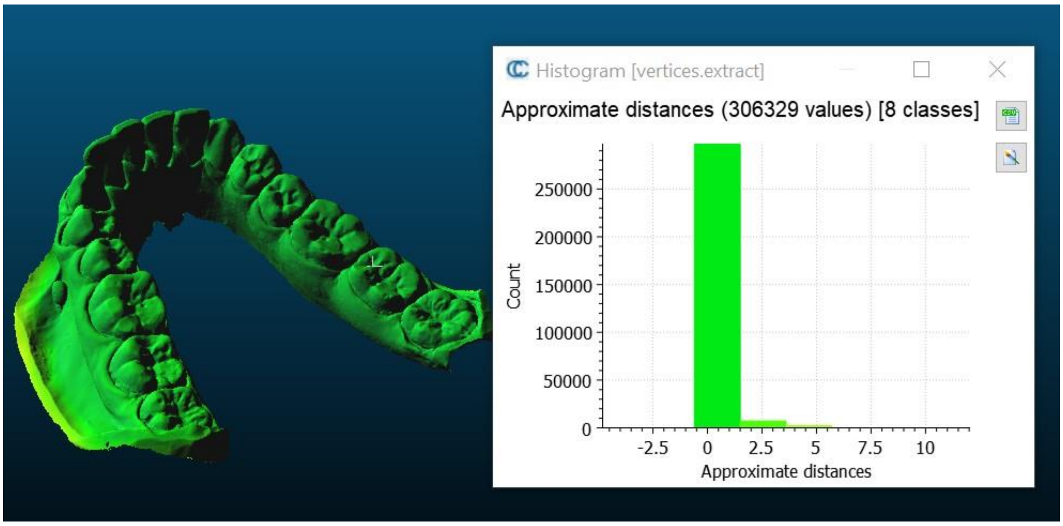

Fig 17: J08 lower jaw .

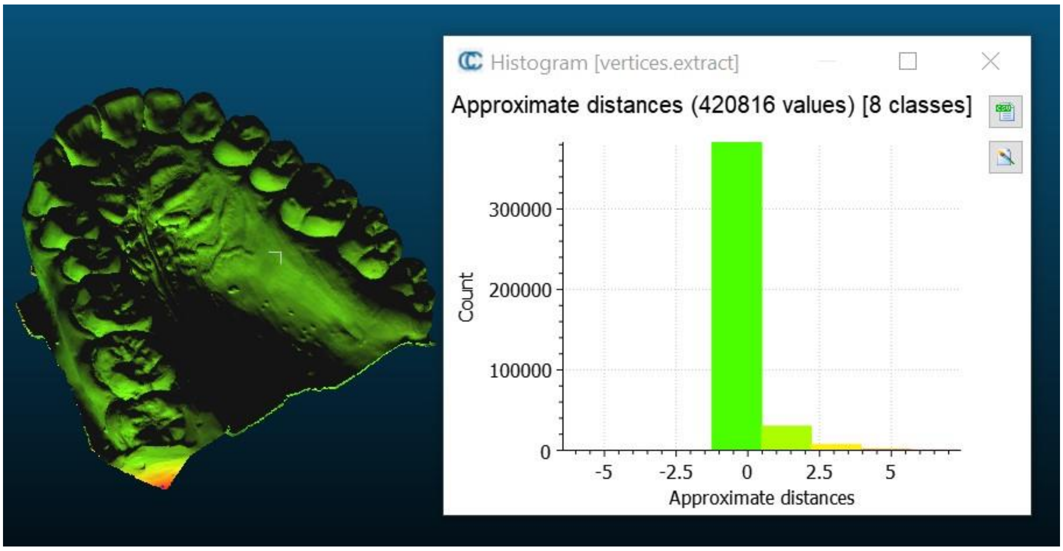

Fig 18: J08 upper jaw .

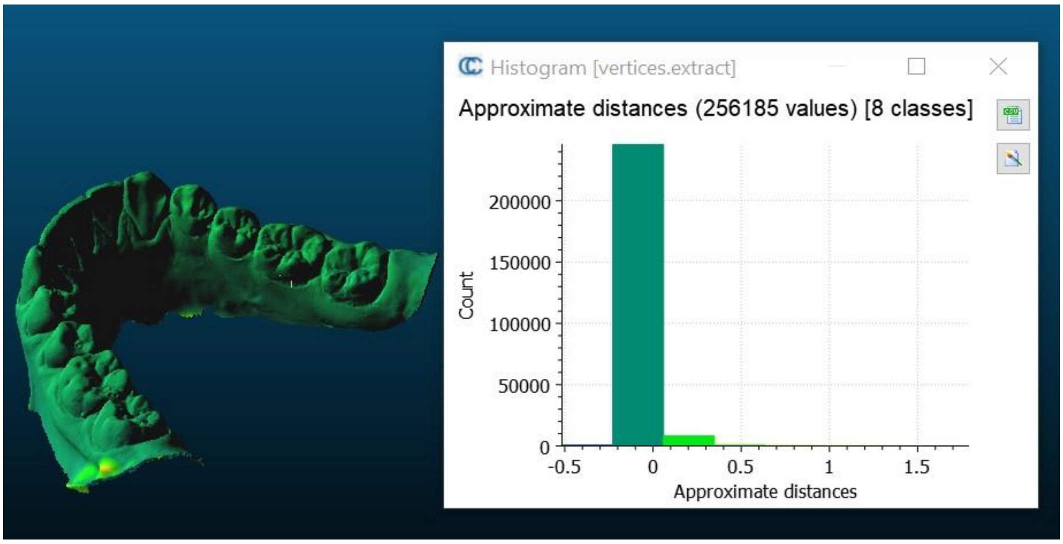

Fig 19: J09 lower jaw .

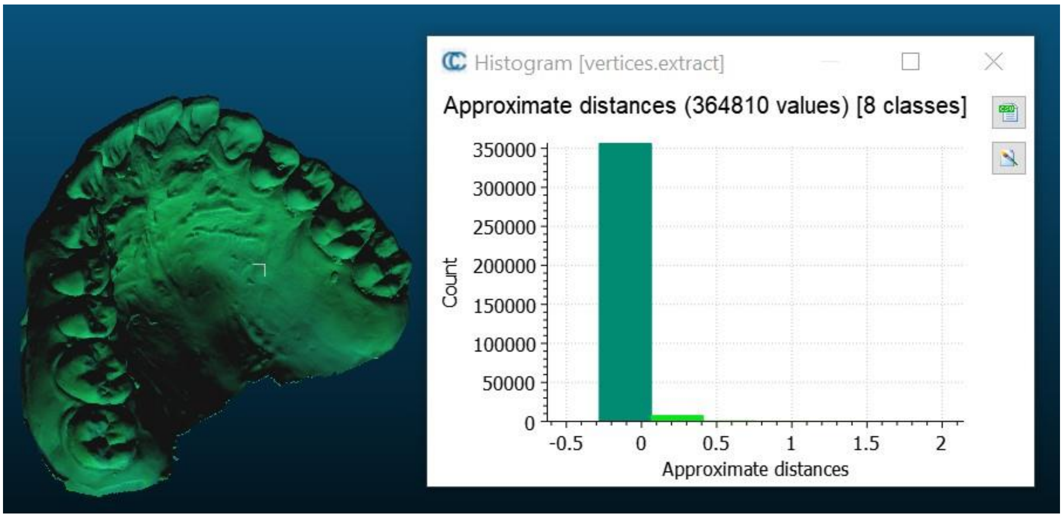

Fig 20: J09 upper jaw .

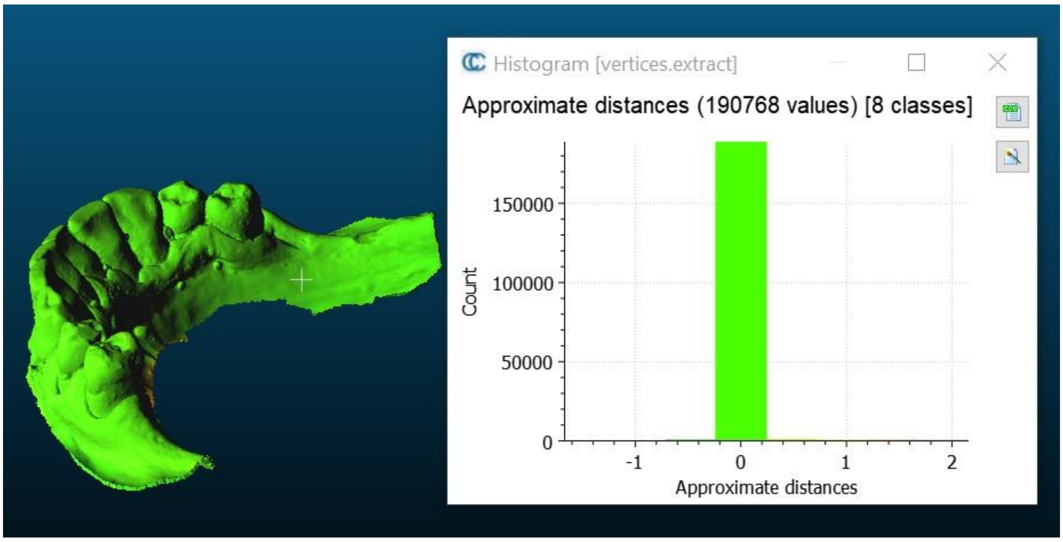

Fig 21: J10 lower jaw .

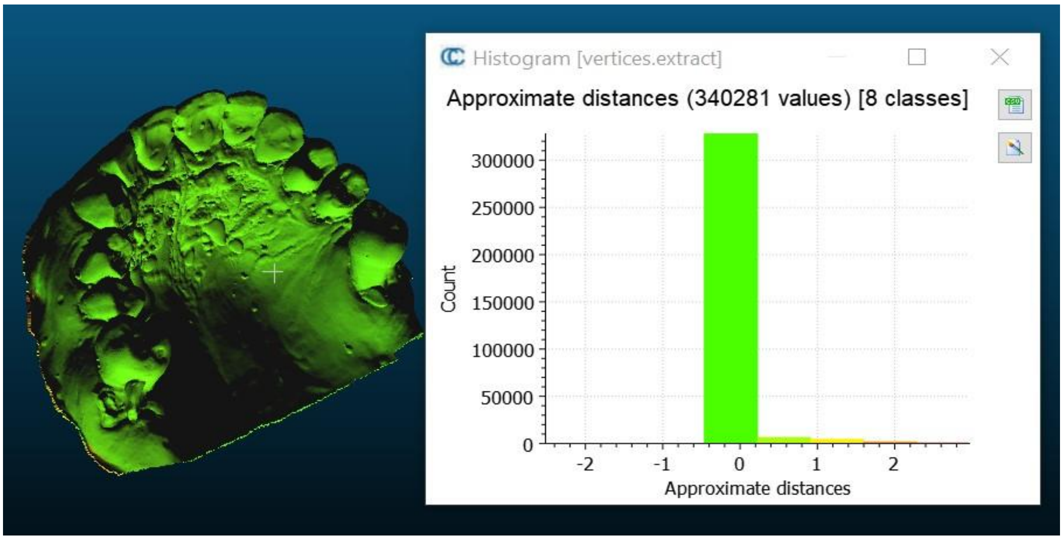

Fig 22: J10 upper jaw .

IOS1 + OP2

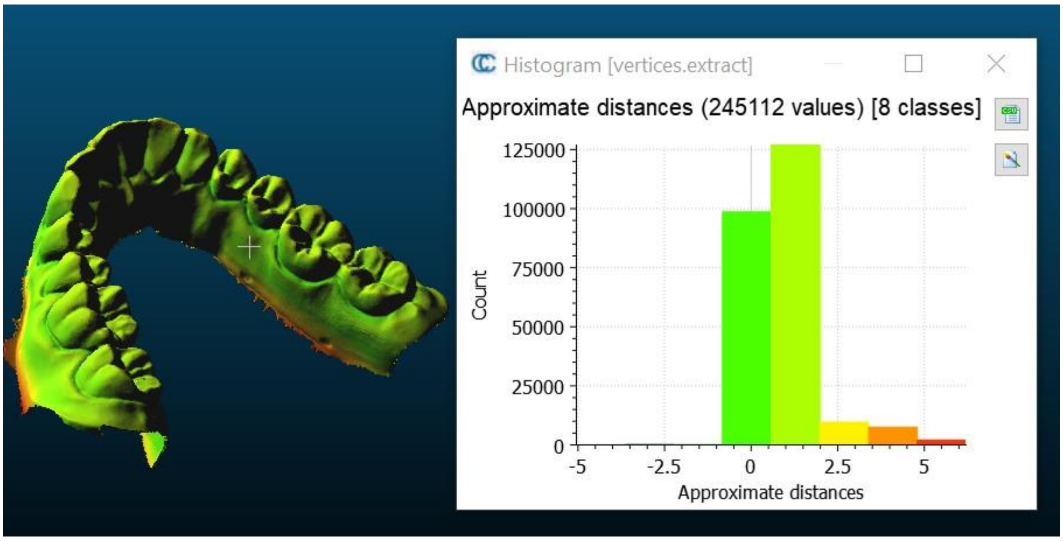

Fig 1: T standard model lower jaw .

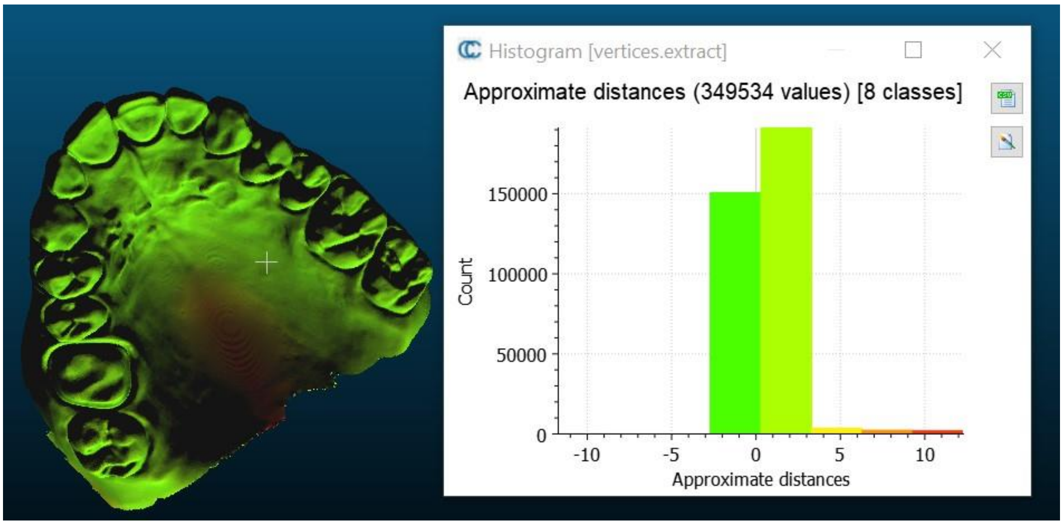

Fig 2: T standard model upper jaw .

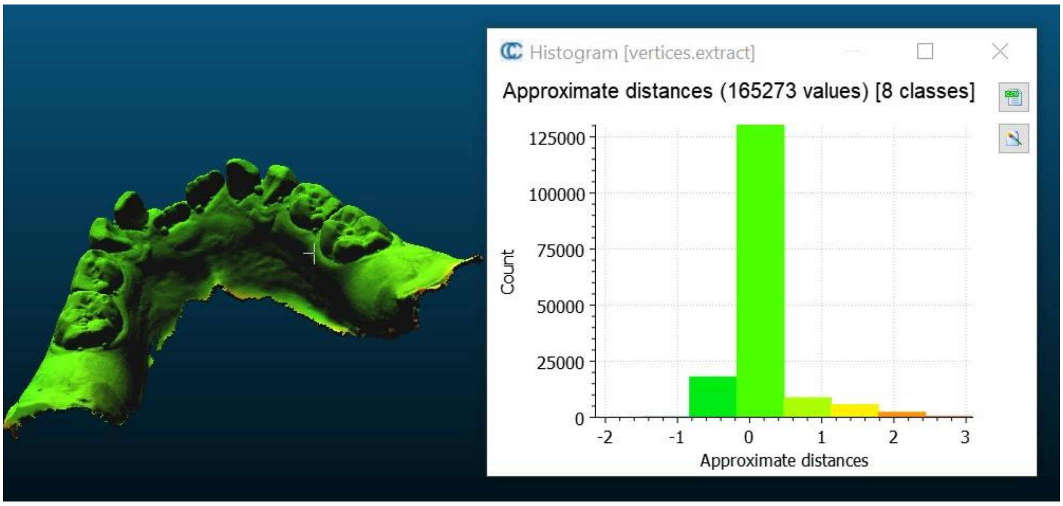

Fig 3: T01 lower jaw .

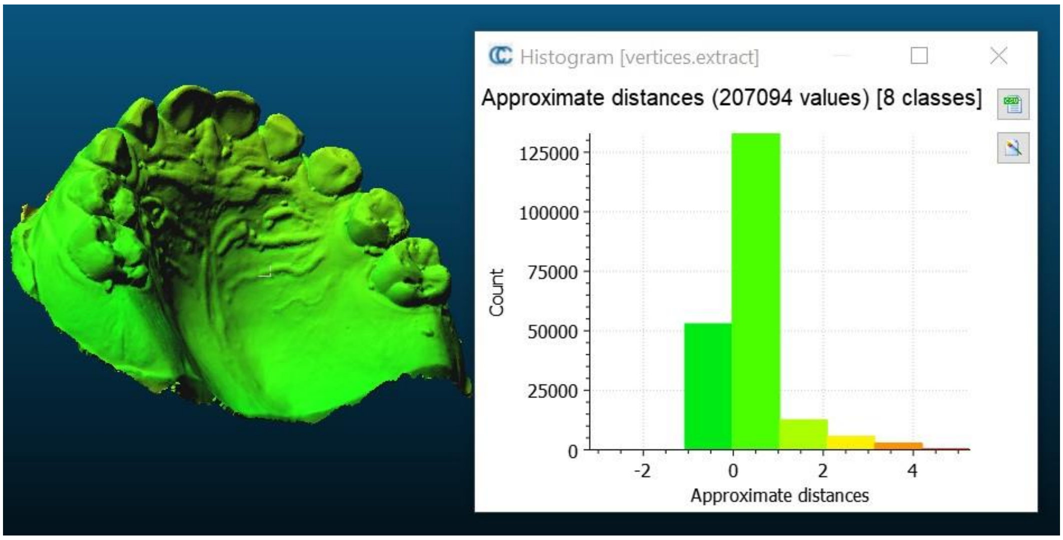

Fig 4: T01 upper jaw .

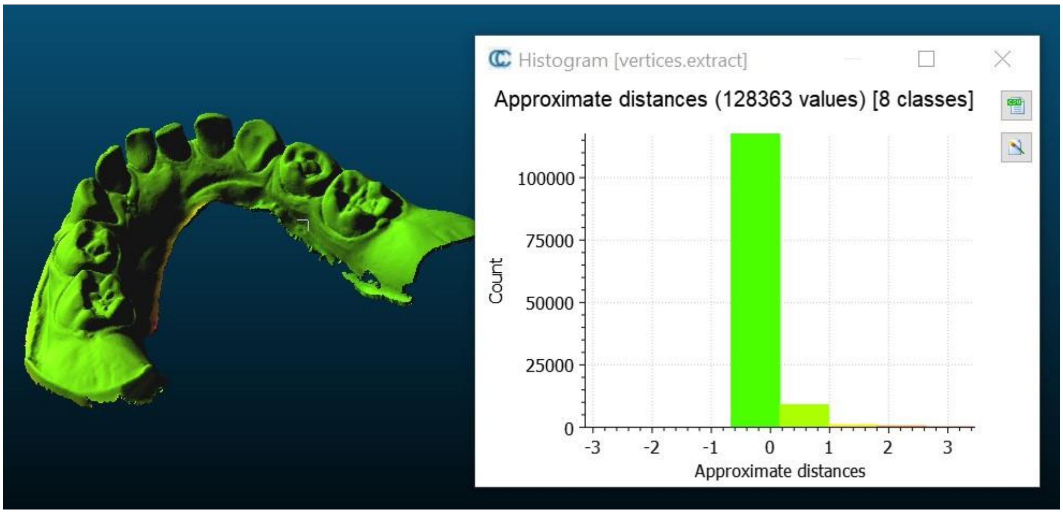

Fig 5: T02 lower jaw .

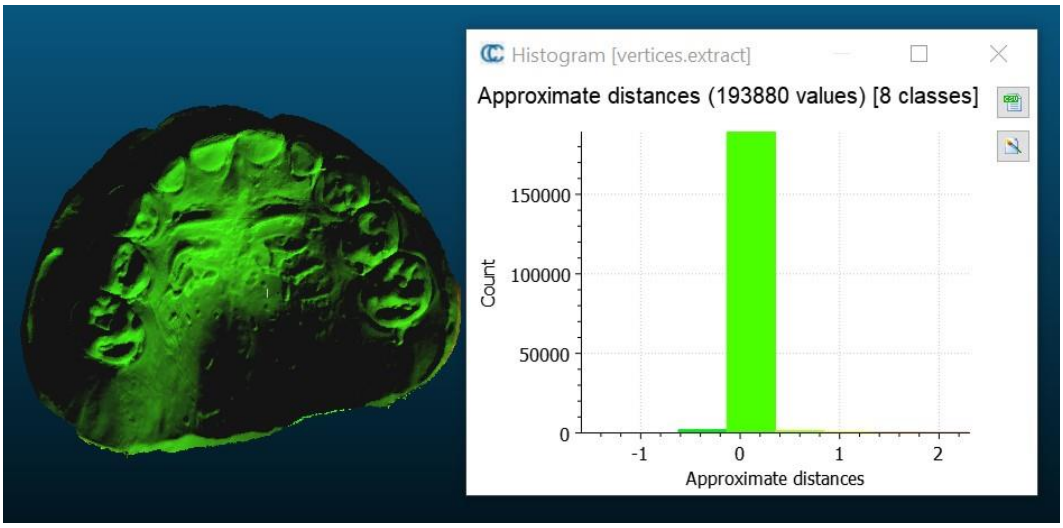

Fig 6: T02 upper jaw .

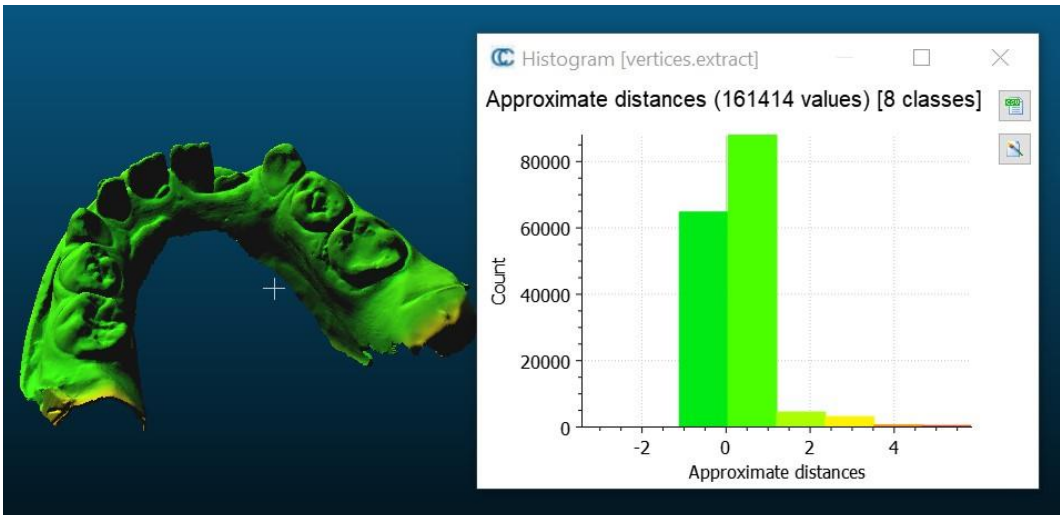

Fig 7: T03 lower jaw .

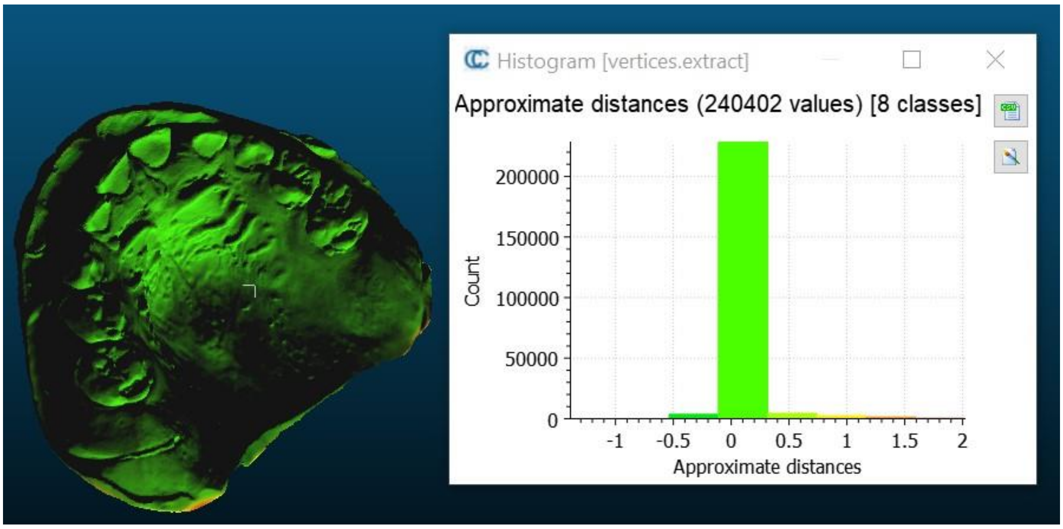

Fig 8: T03 upper jaw .

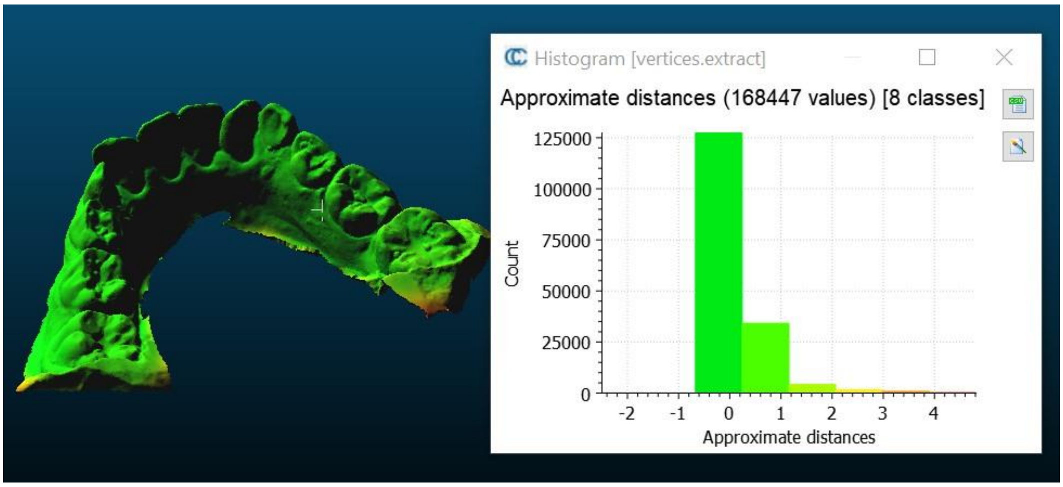

Fig 9: T04 lower jaw .

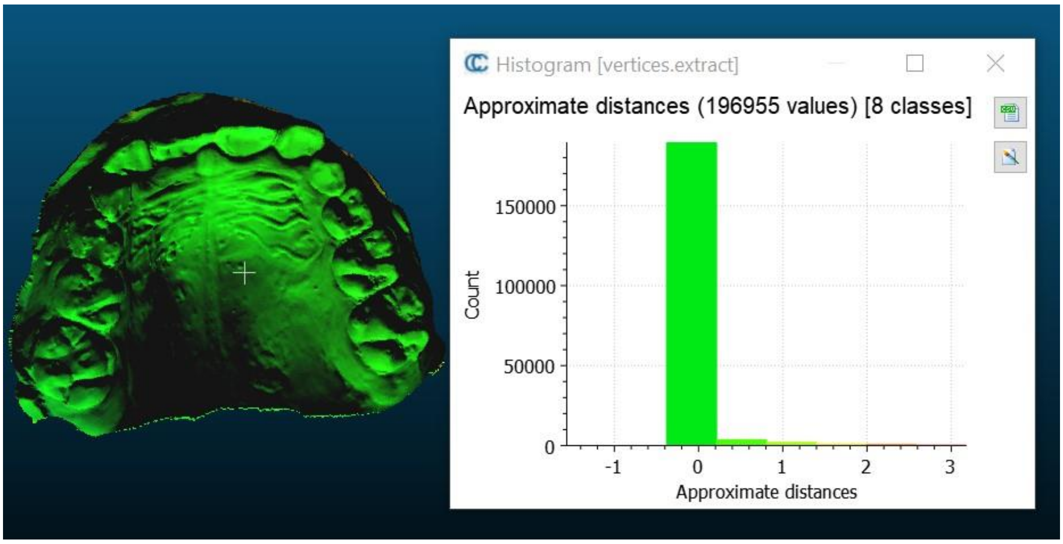

Fig 10: T04 upper jaw .

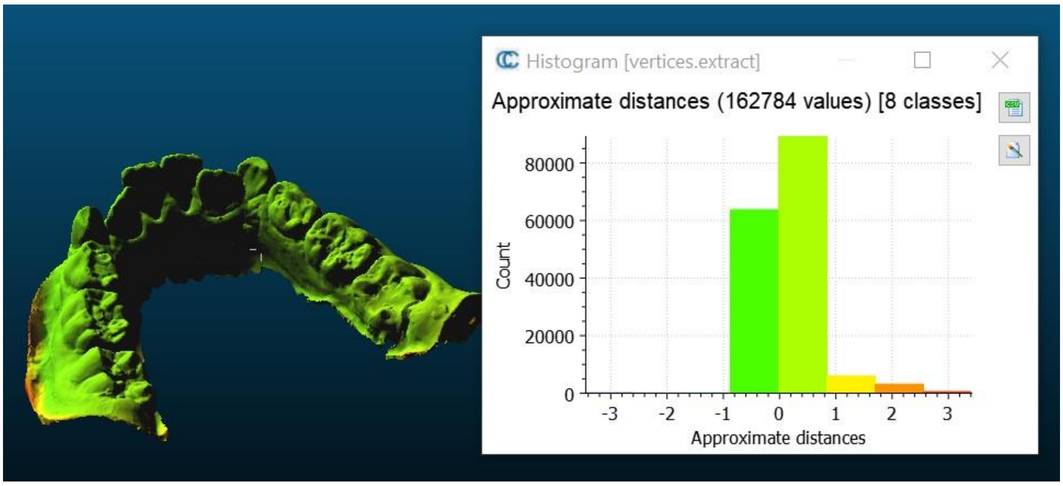

Fig 11: T05 lower jaw .

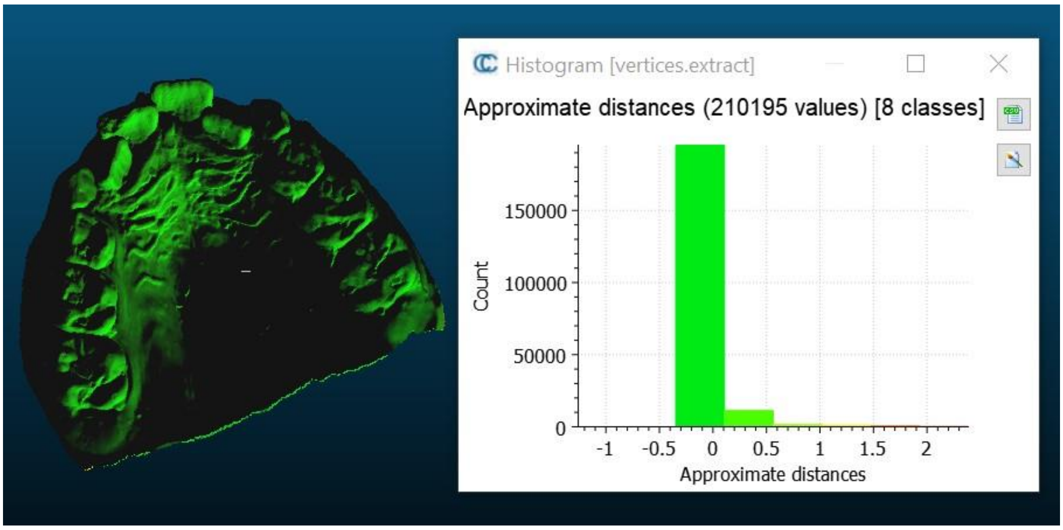

Fig 12: T05 upper jaw .

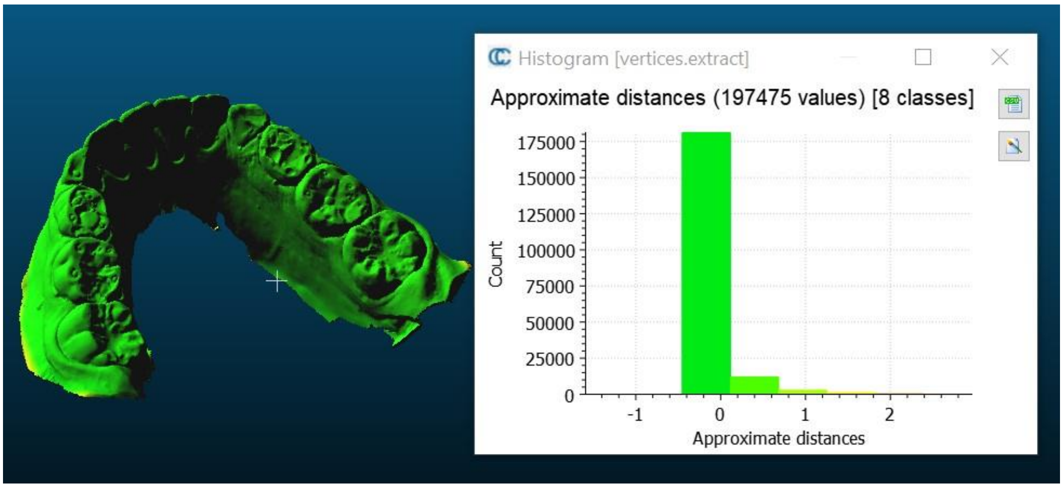

Fig 13: T06 lower jaw .

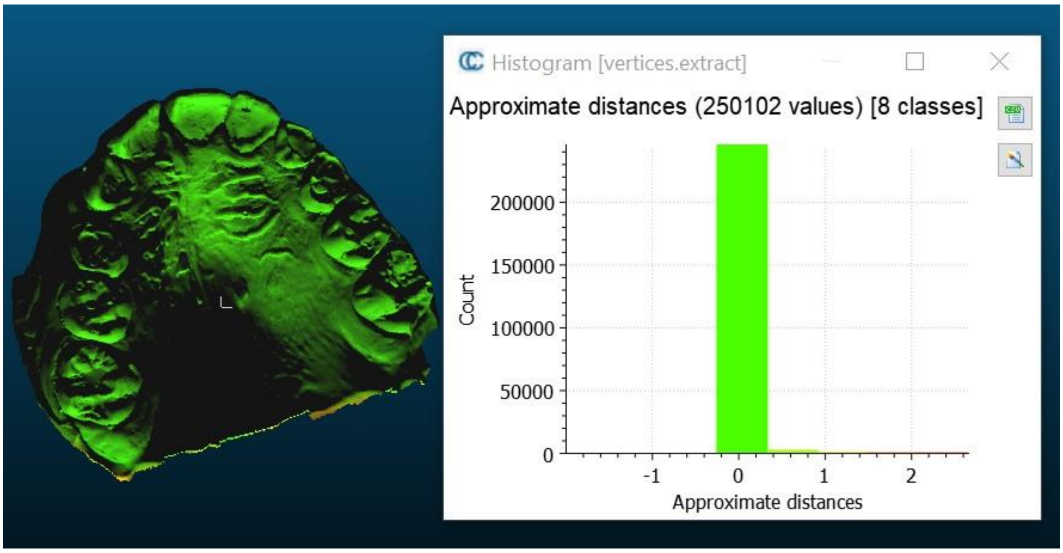

Fig 14: T06 upper jaw .

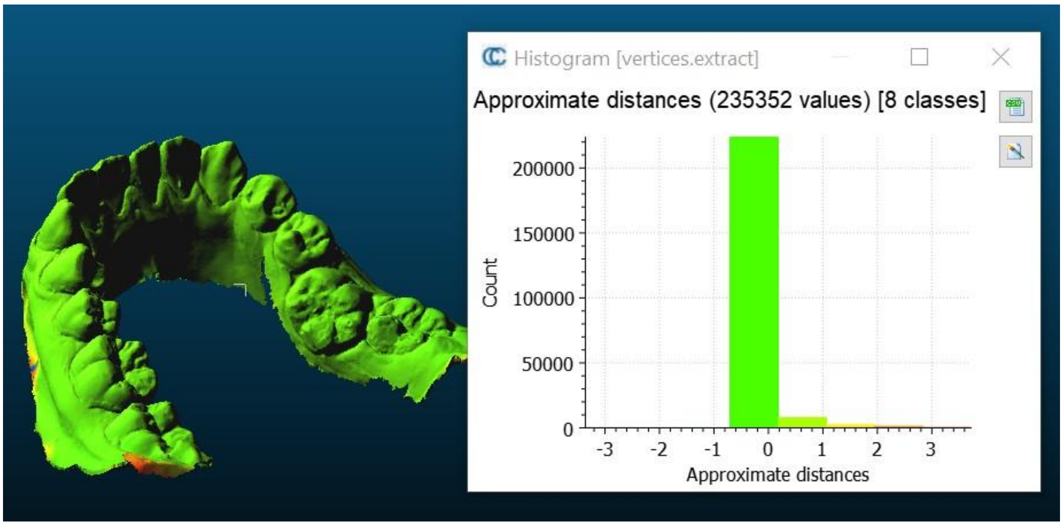

Fig 15: T07 lower jaw .

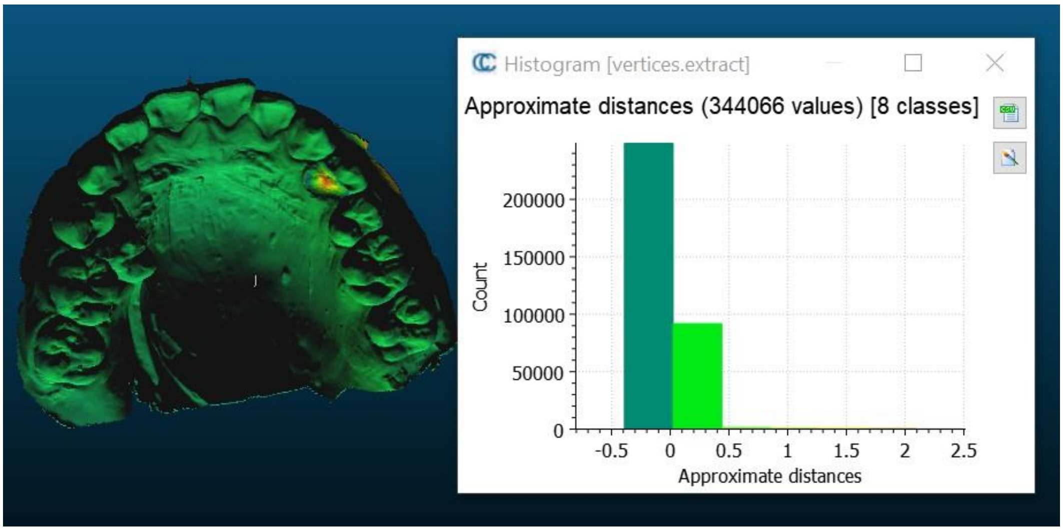

Fig 16: T07 upper jaw .

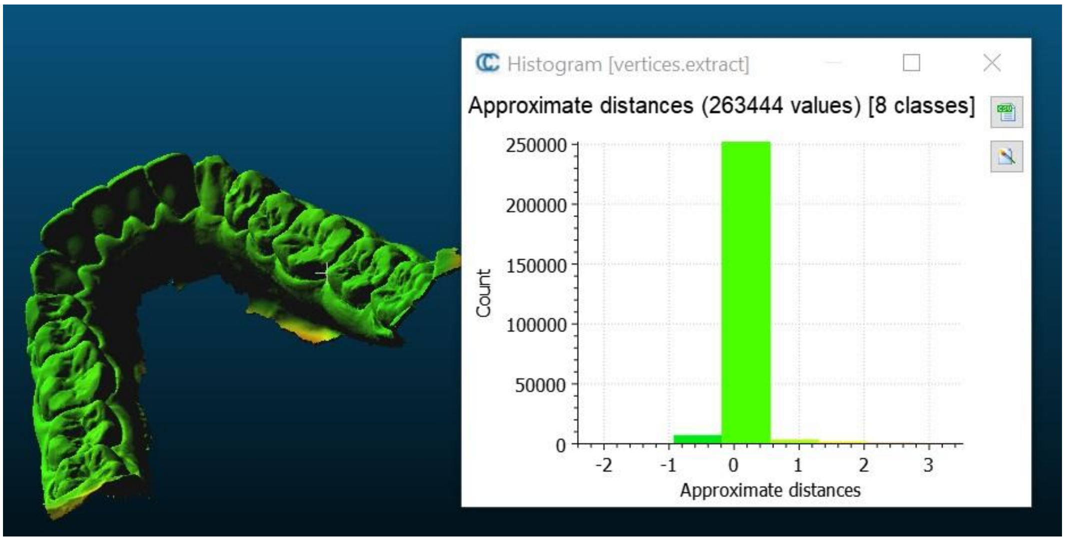

Fig 17: T08 lower jaw .

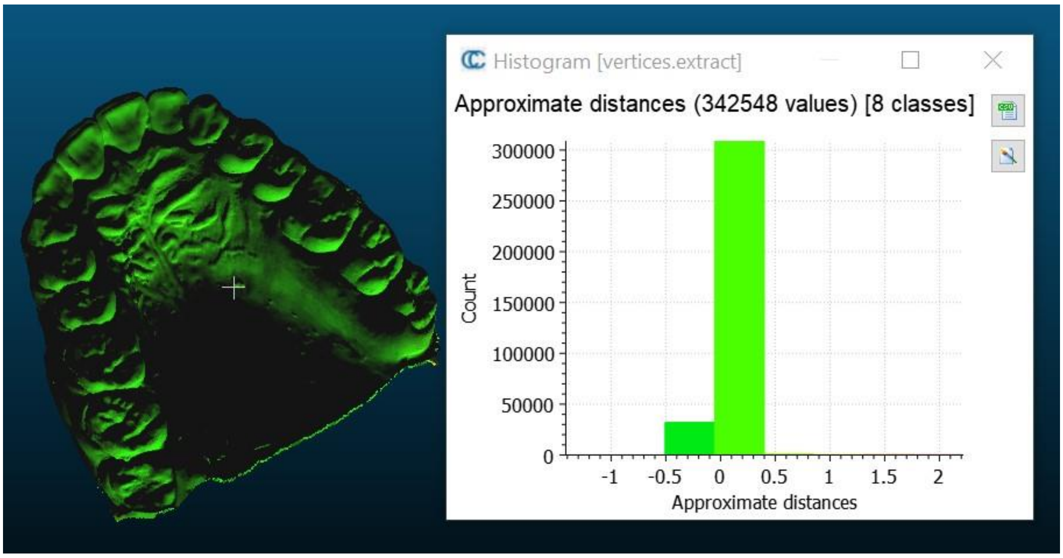

Fig 18: T08 upper jaw .

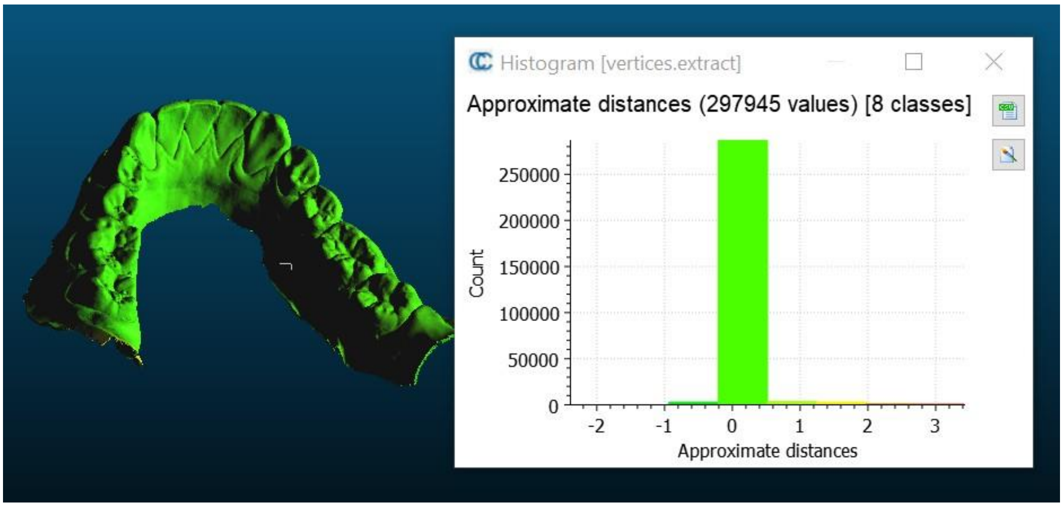

Fig 19: T09 lower jaw .

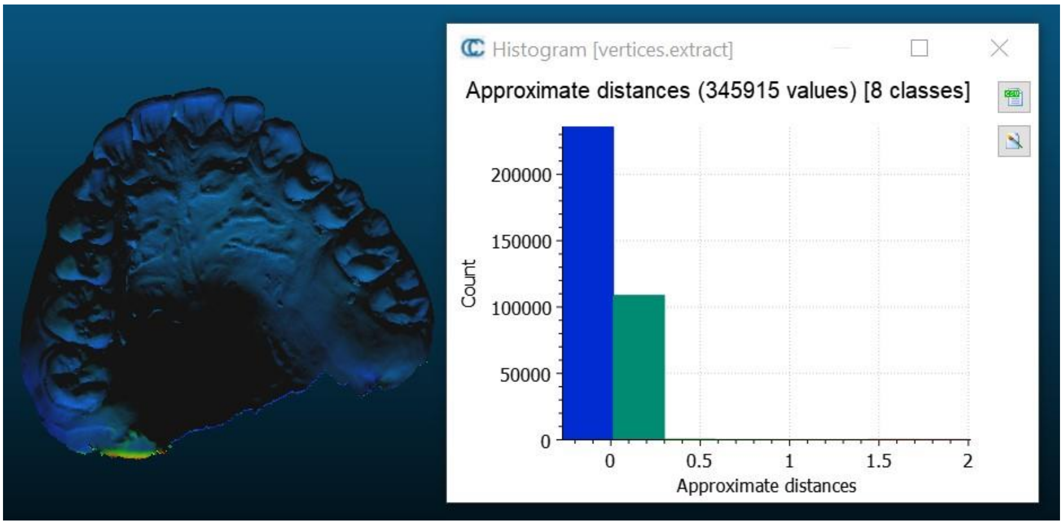

Fig 20: T09 upper jaw .

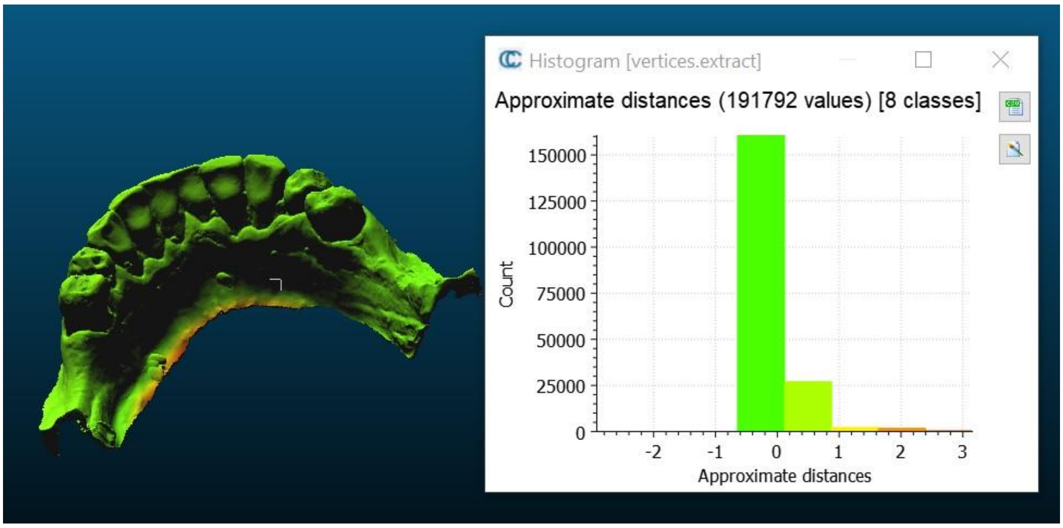

Fig 21: T10 lower jaw .

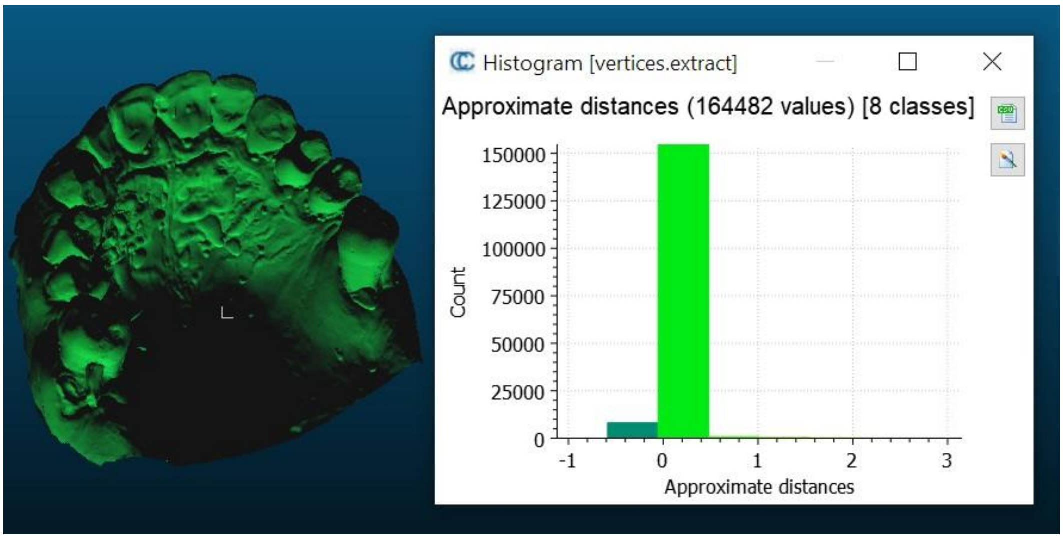

Fig 22: T10 upper jaw .

IOS2 + OP1

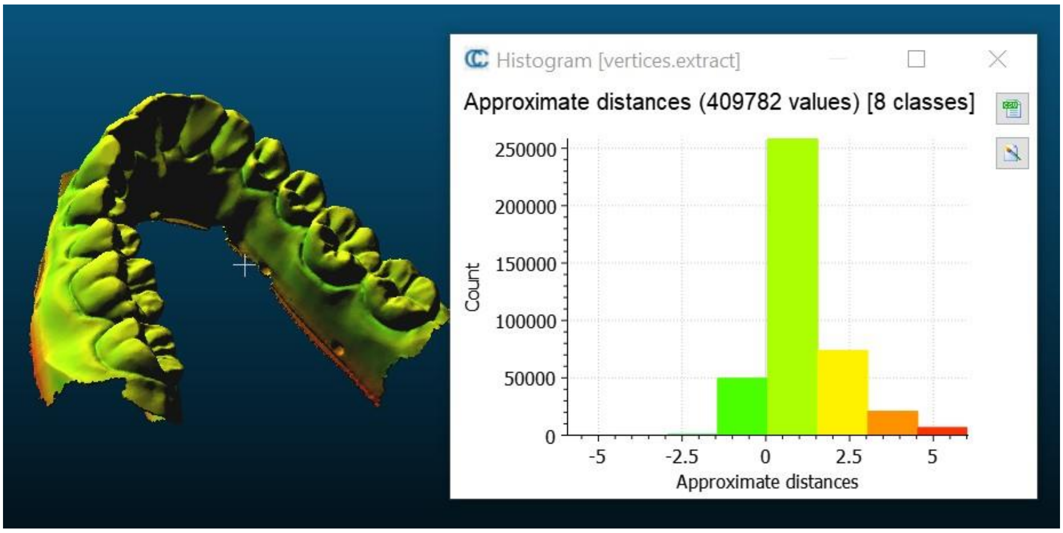

Fig 1: J standard model lower jaw .

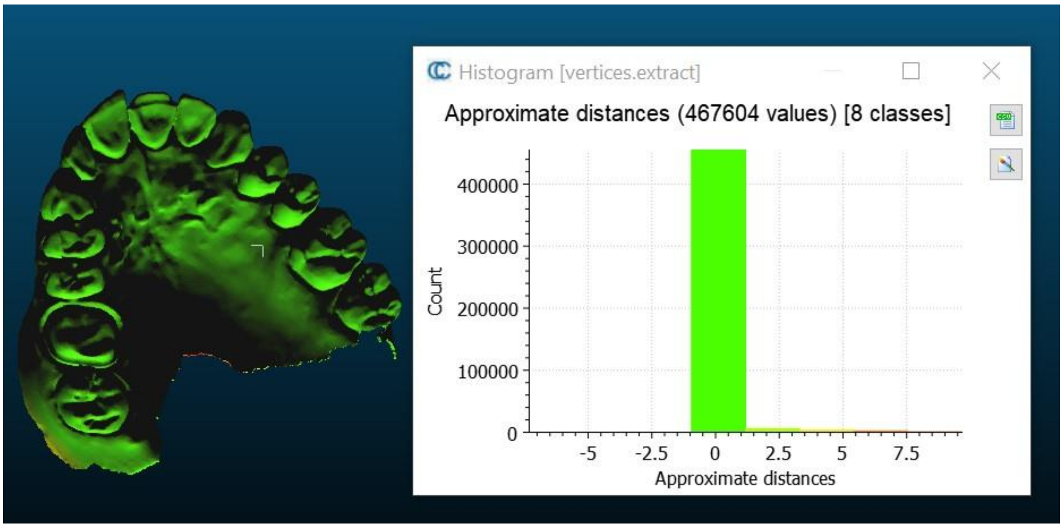

Fig 2: J standard model upper jaw .

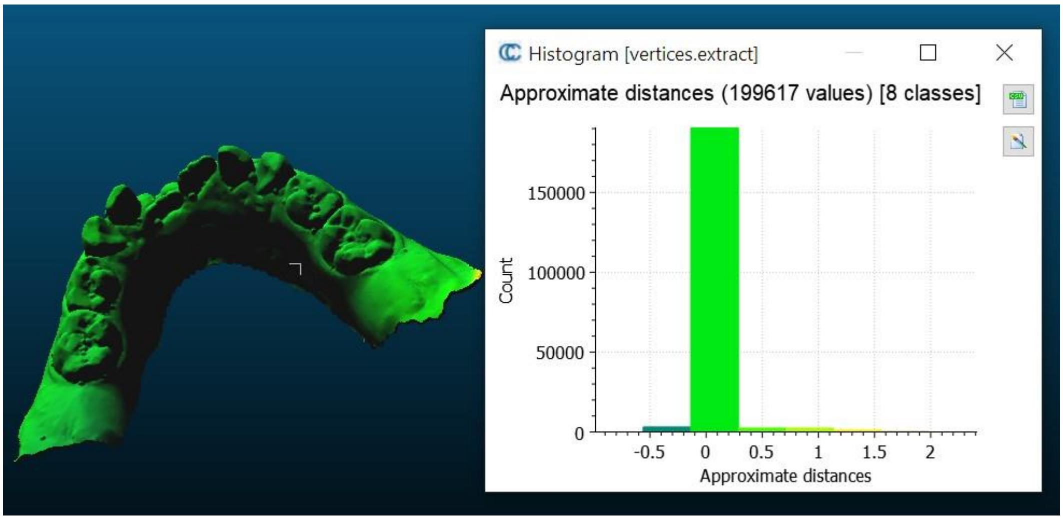

Fig 3: J01 lower jaw .

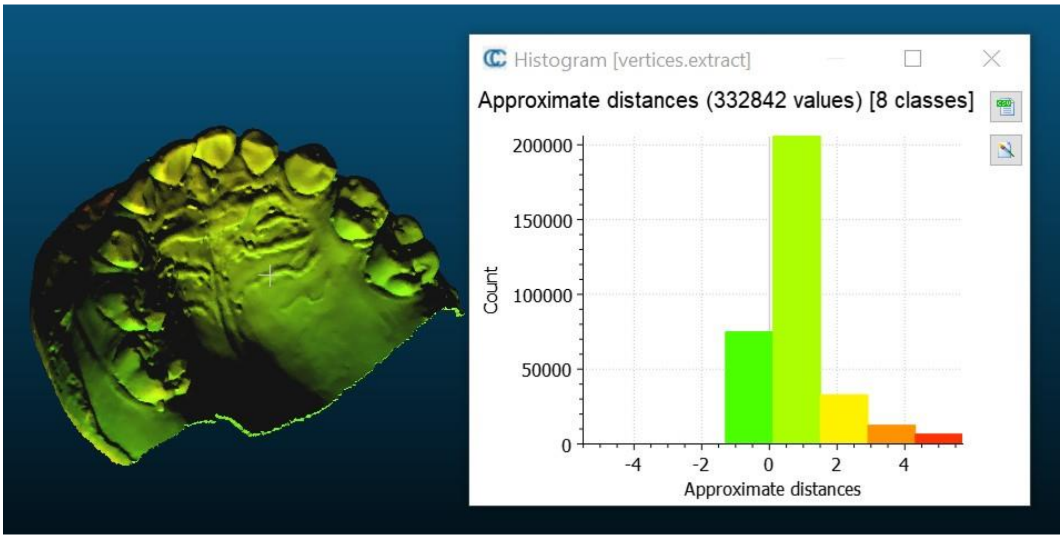

Fig 4: J01 upper jaw .

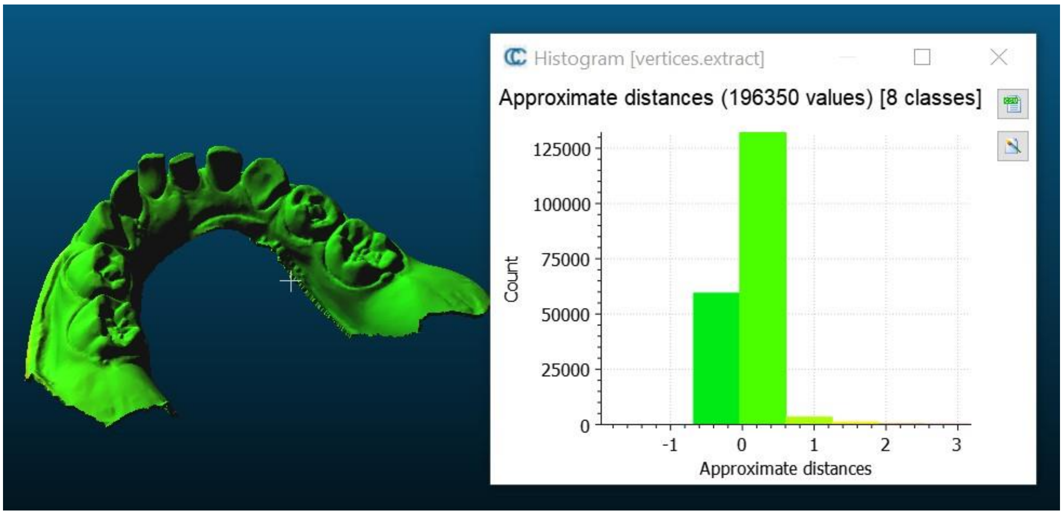

Fig 5: J02 lower jaw .

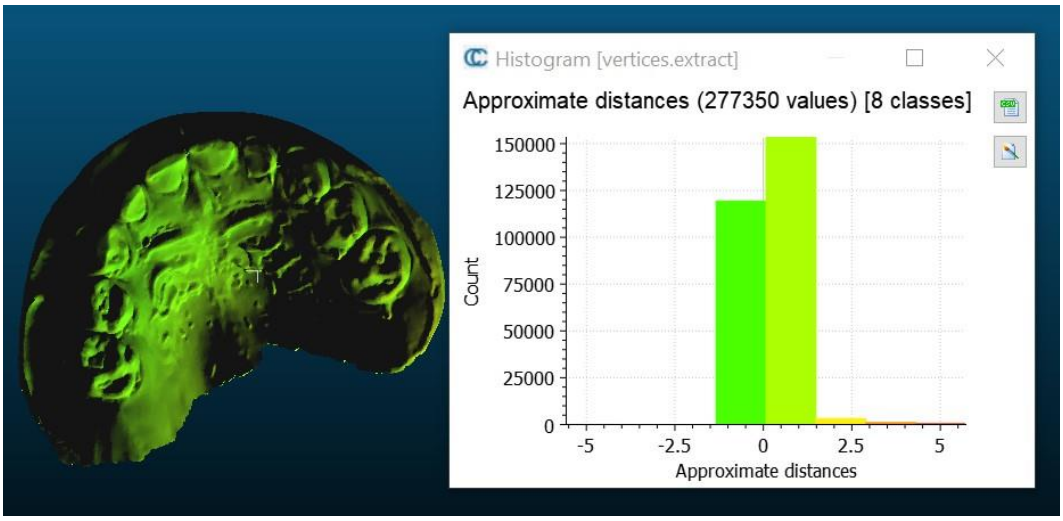

Fig 6: J02 upper jaw .

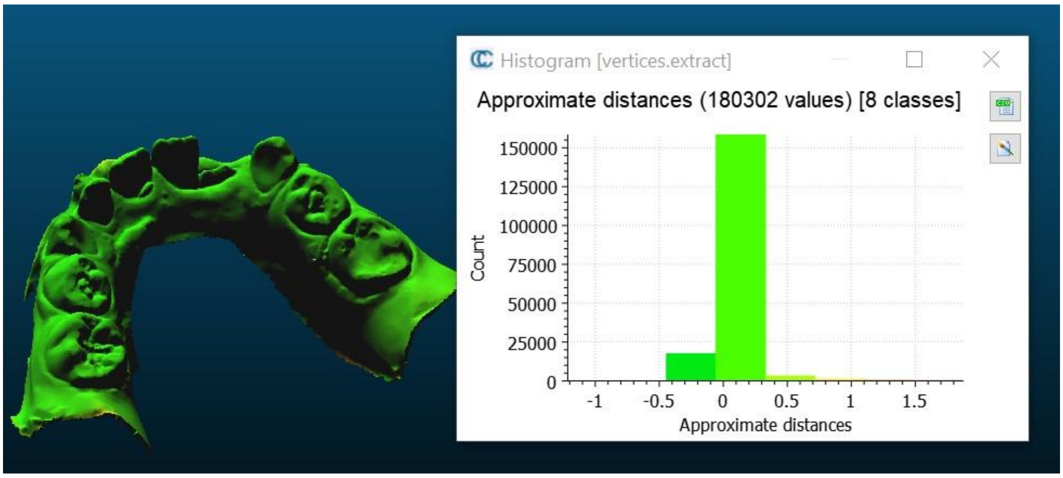

Fig 7: J03 lower jaw .

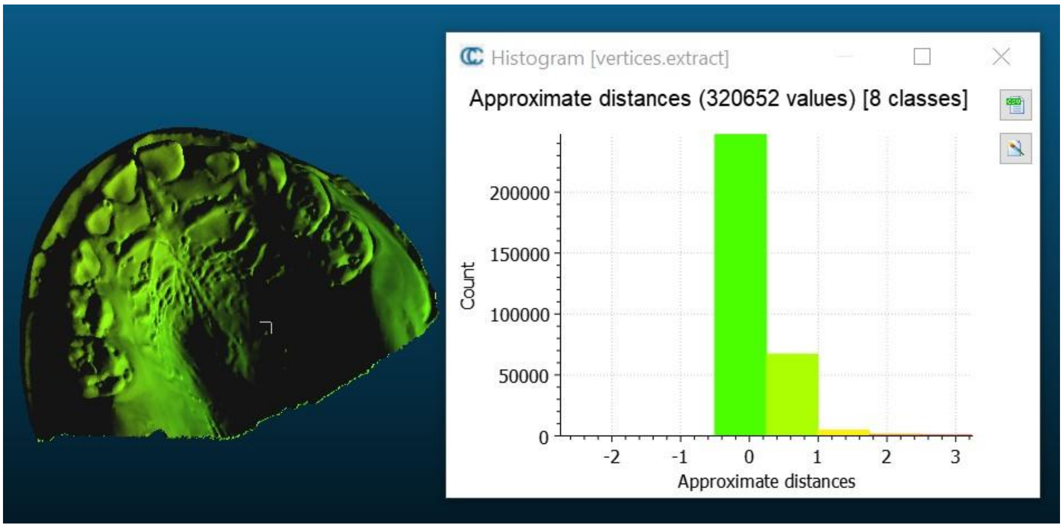

Fig 8: J03 upper jaw .

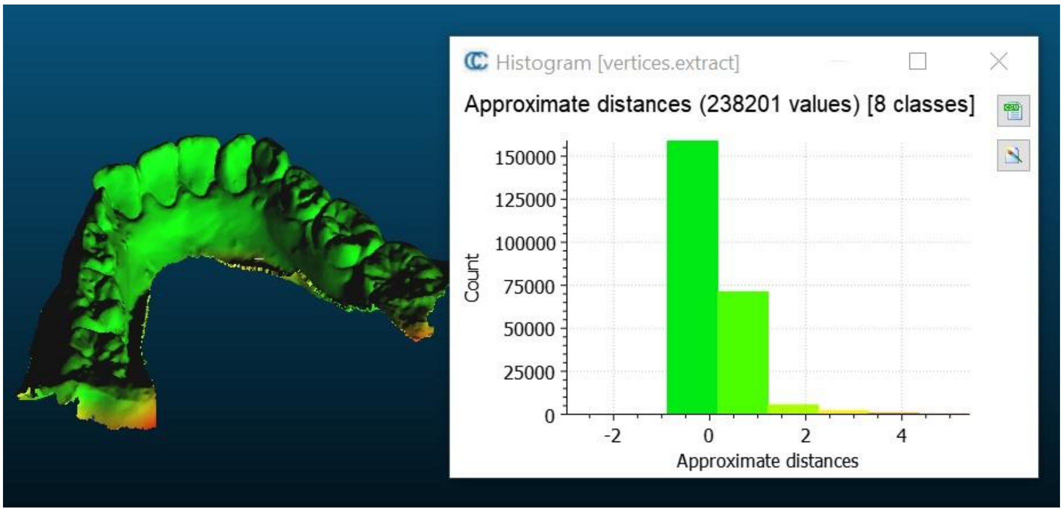

Fig 9: J04 lower jaw .

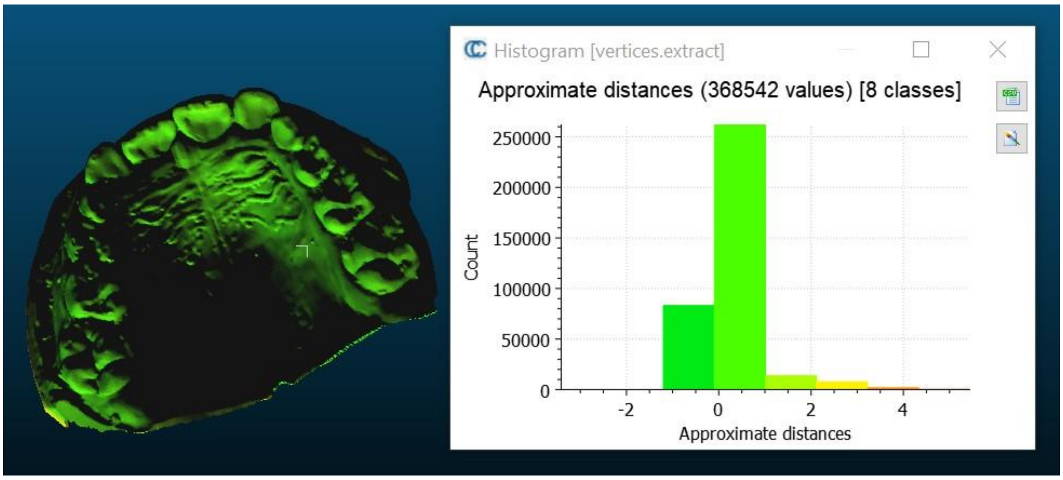

Fig 10: J04 upper jaw .

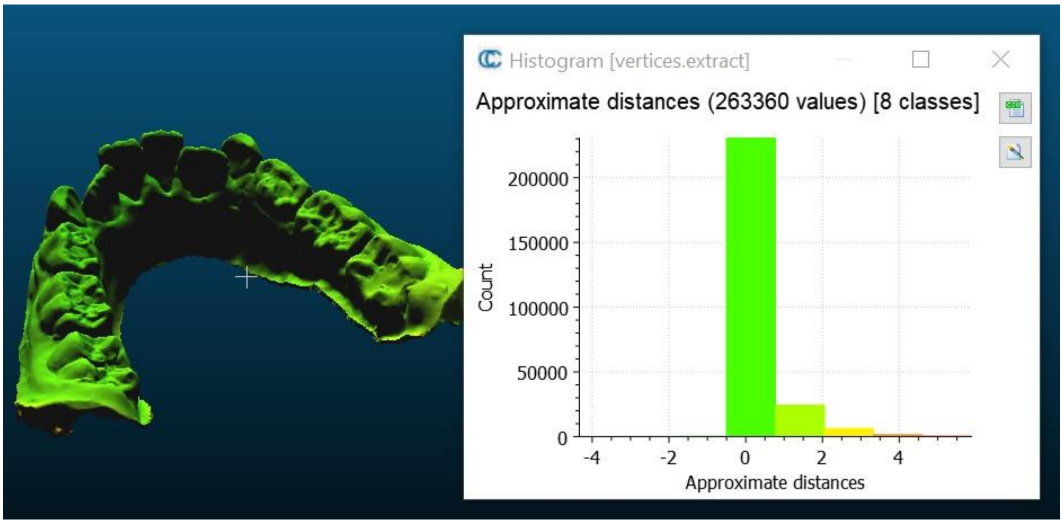

Fig 11: J05 lower jaw .

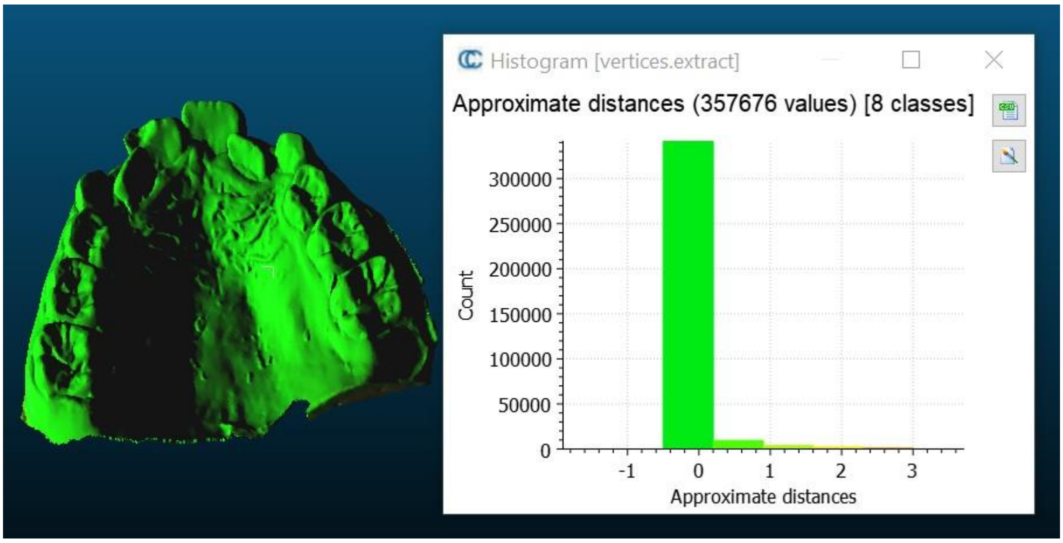

Fig 12: J05 upper jaw .

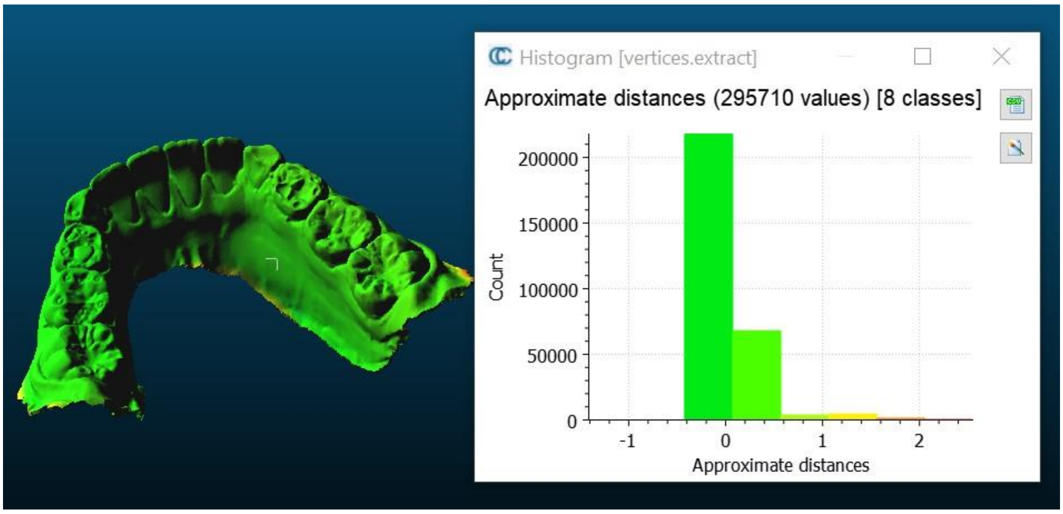

Fig 13: J06 lower jaw .

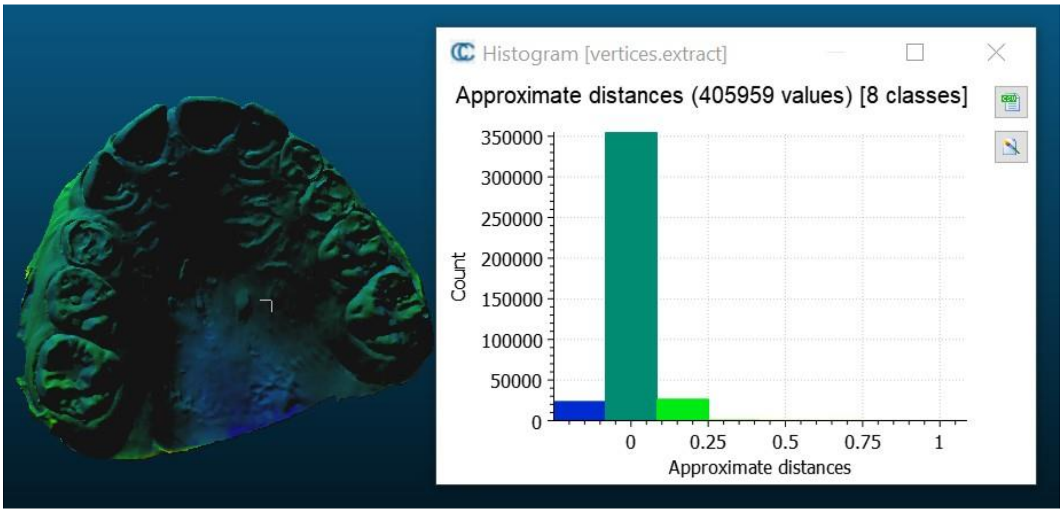

Fig 14: J06 upper jaw .

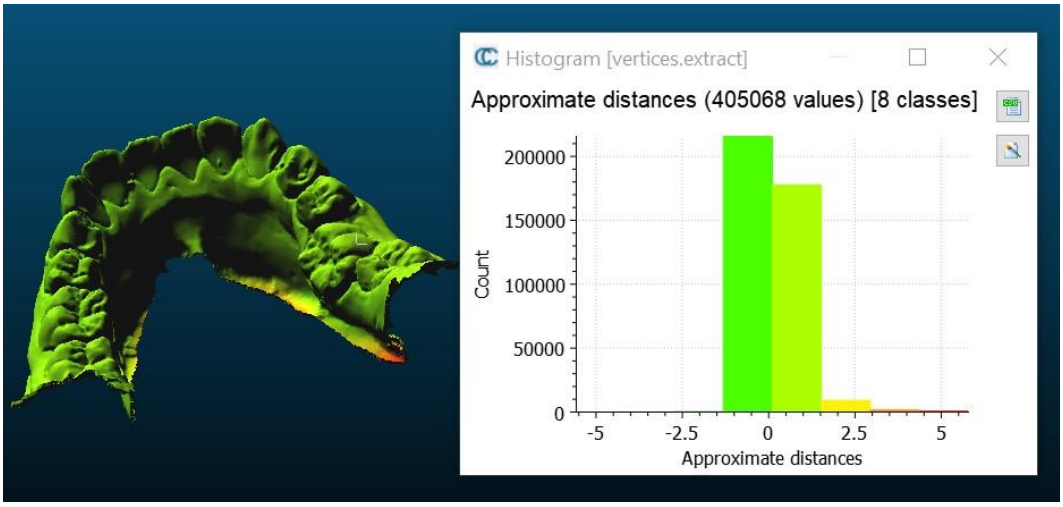

Fig 15: J07 lower jaw .

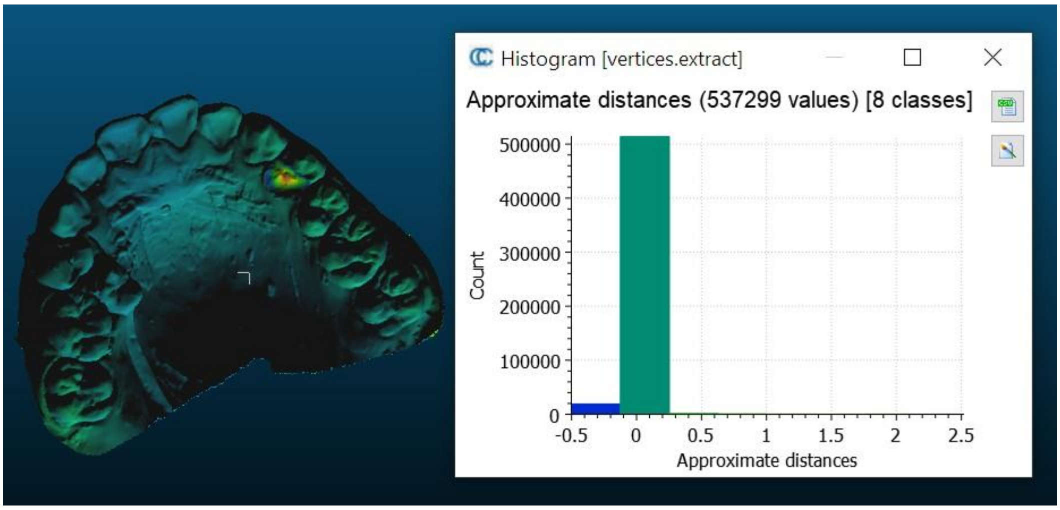

Fig 16: J07 upper jaw .

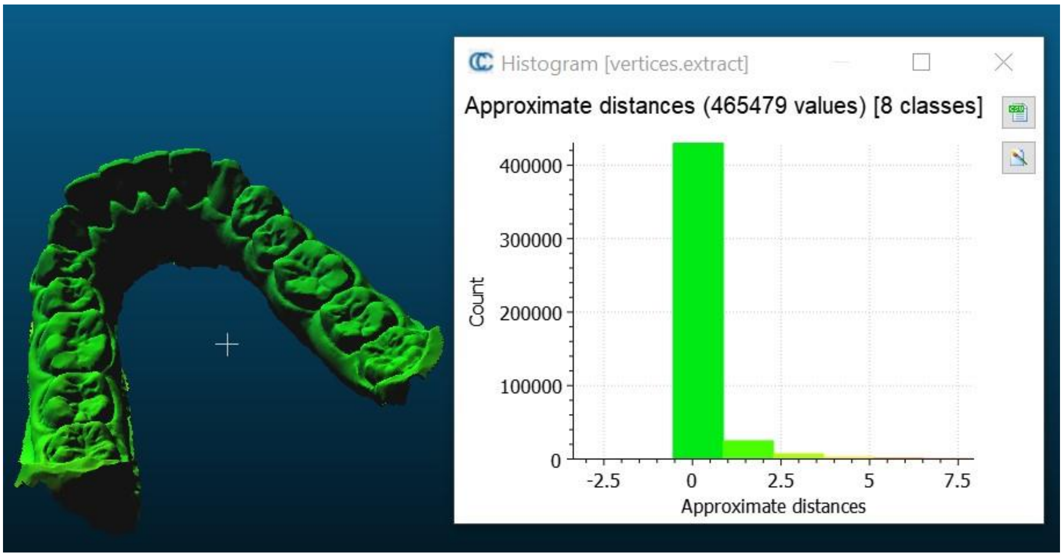

Fig 17: J08 lower jaw .

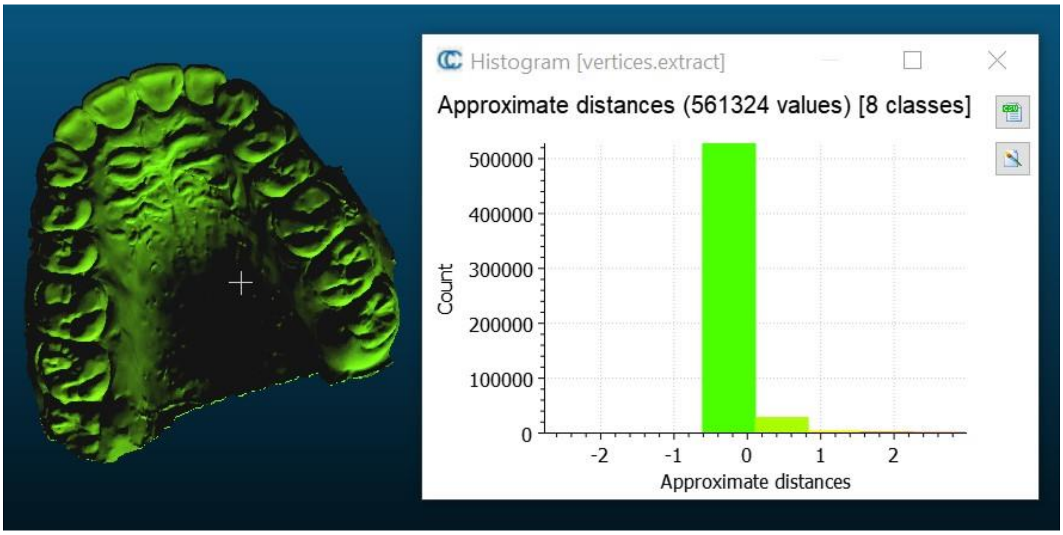

Fig 18: J08 upper jaw .

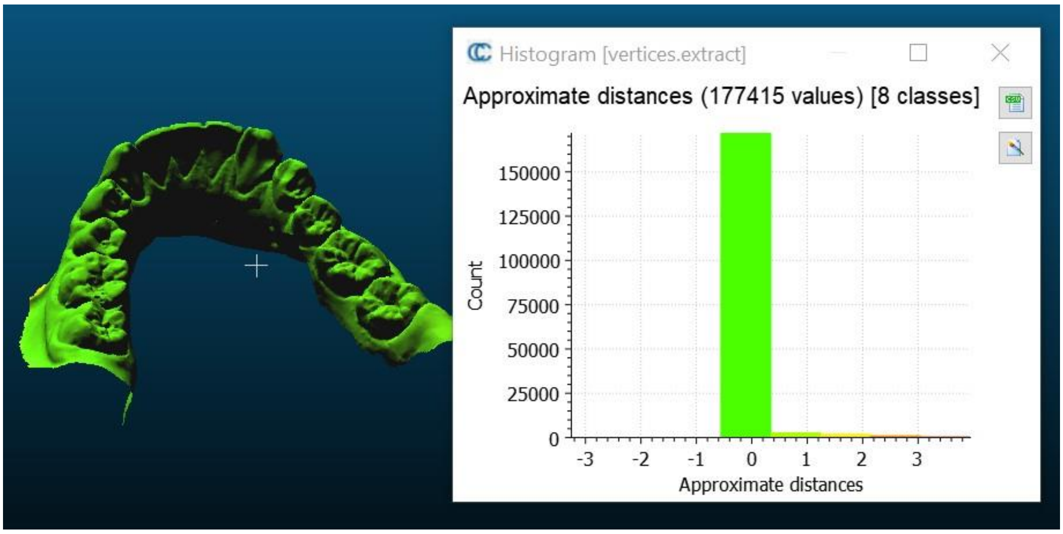

Fig 19: J09 lower jaw .

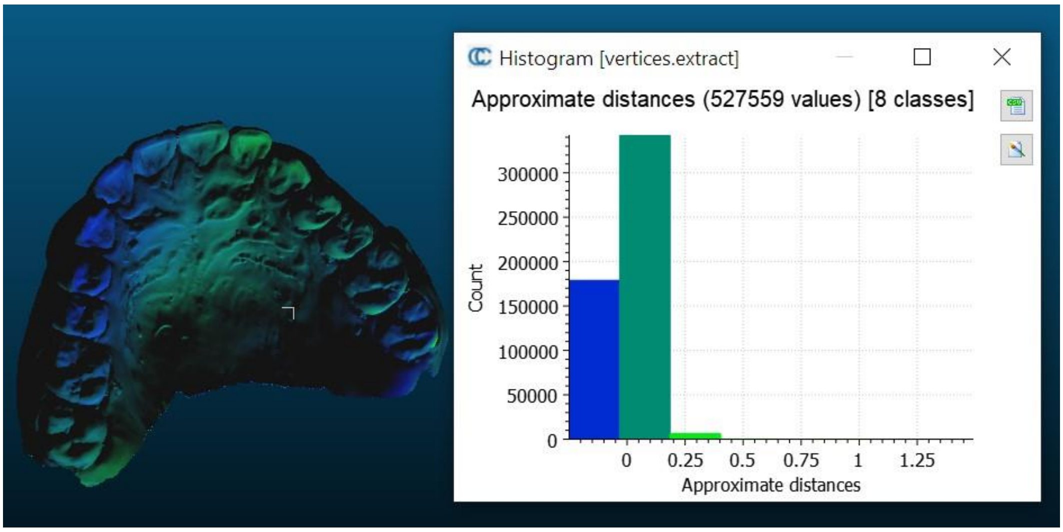

Fig 20: J09 upper jaw .

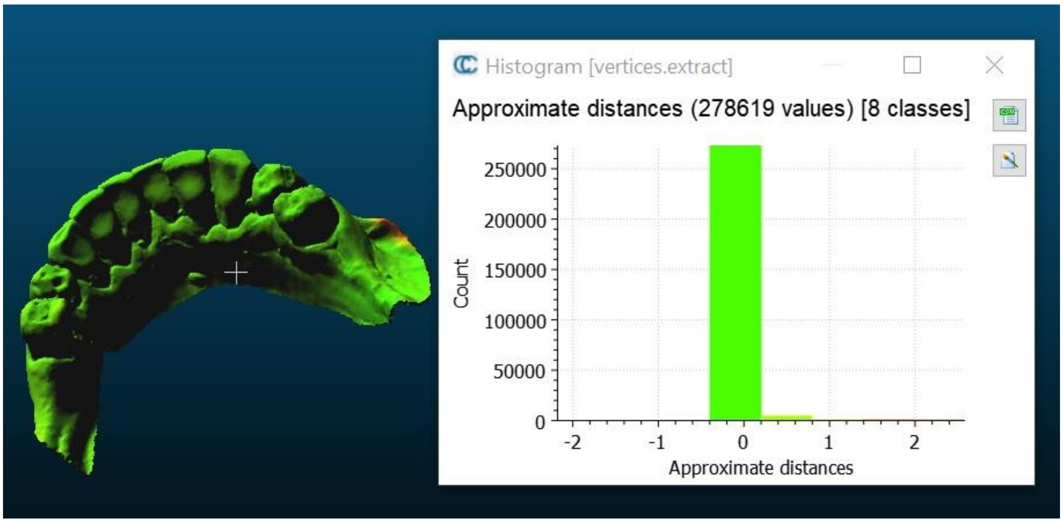

Fig 21: J10 lower jaw .

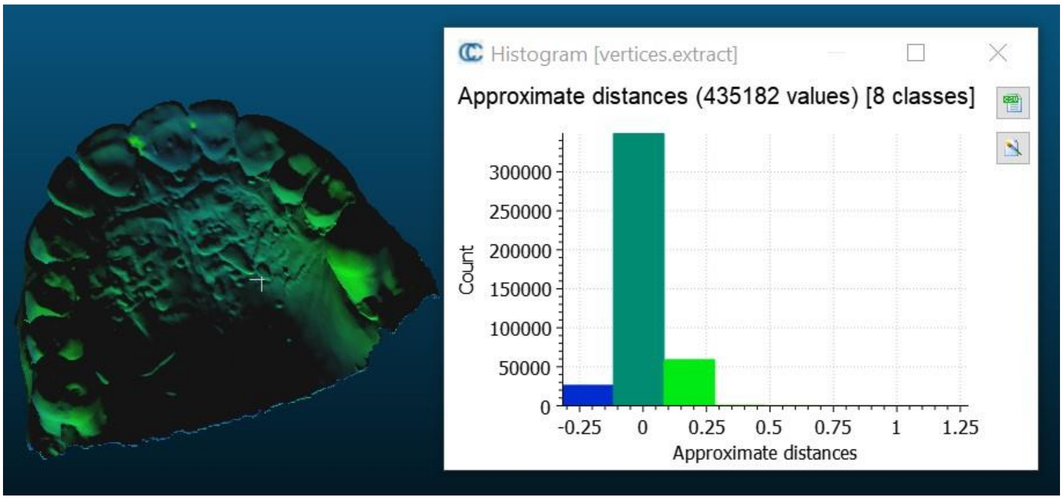

Fig 22: J10 upper jaw .

IOS2 + OP2

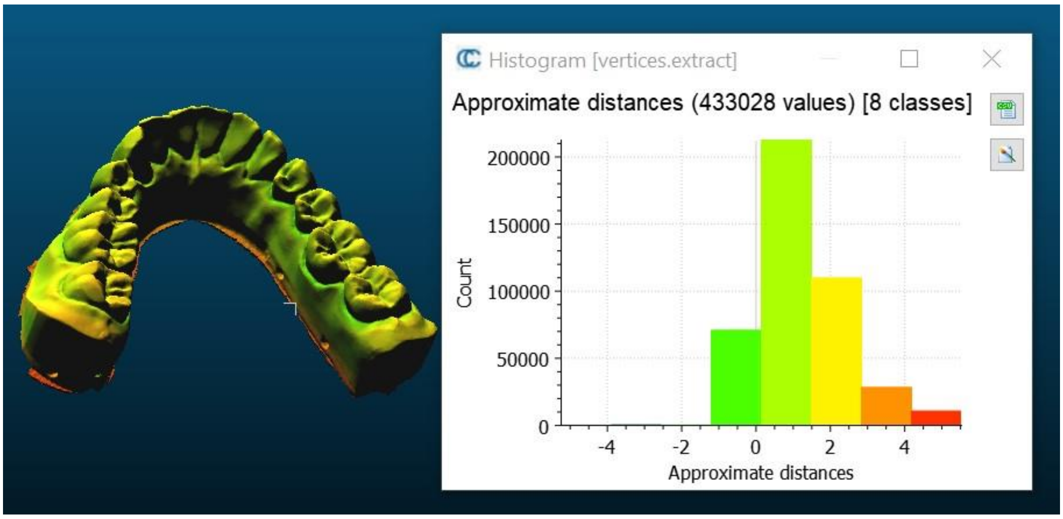

Fig 1: T standard model lower jaw .

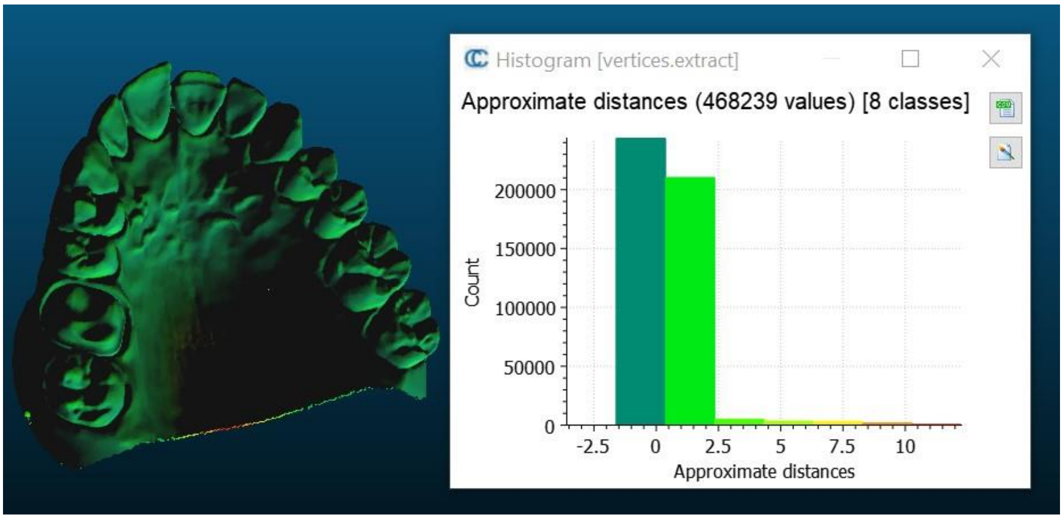

Fig 2: T standard model upper jaw .

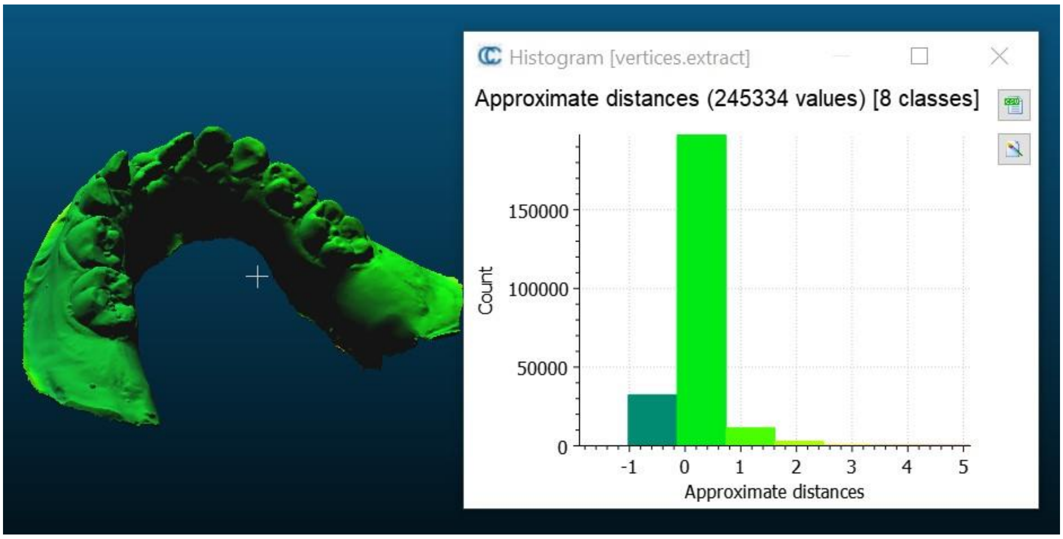

Fig 3: T01 lower jaw .

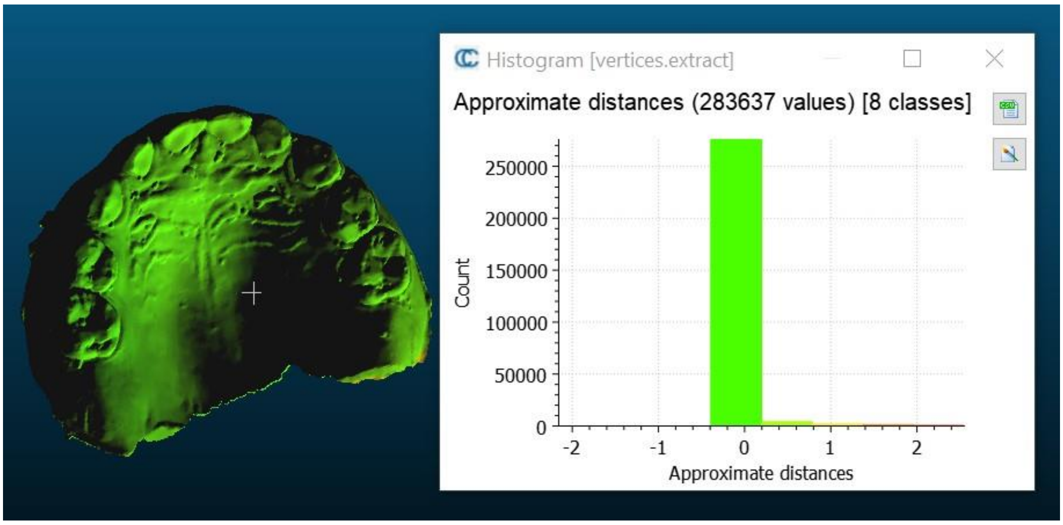

Fig 4: T01 upper jaw .

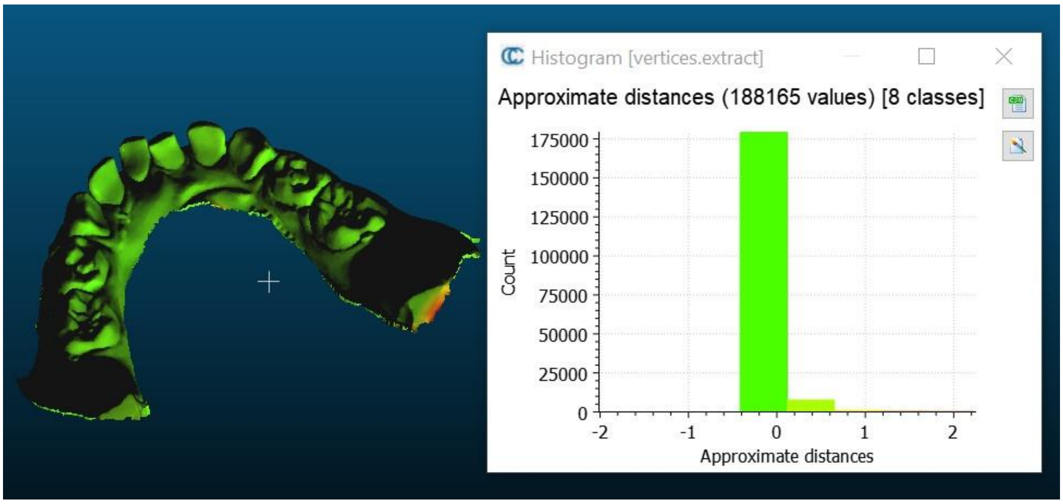

Fig 5: T02 lower jaw .

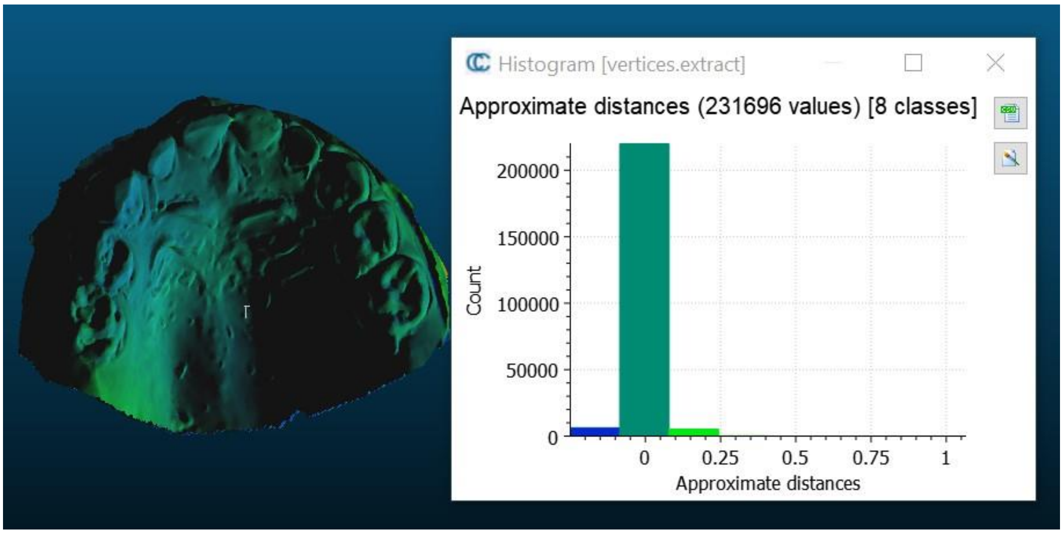

Fig 6: T02 upper jaw .

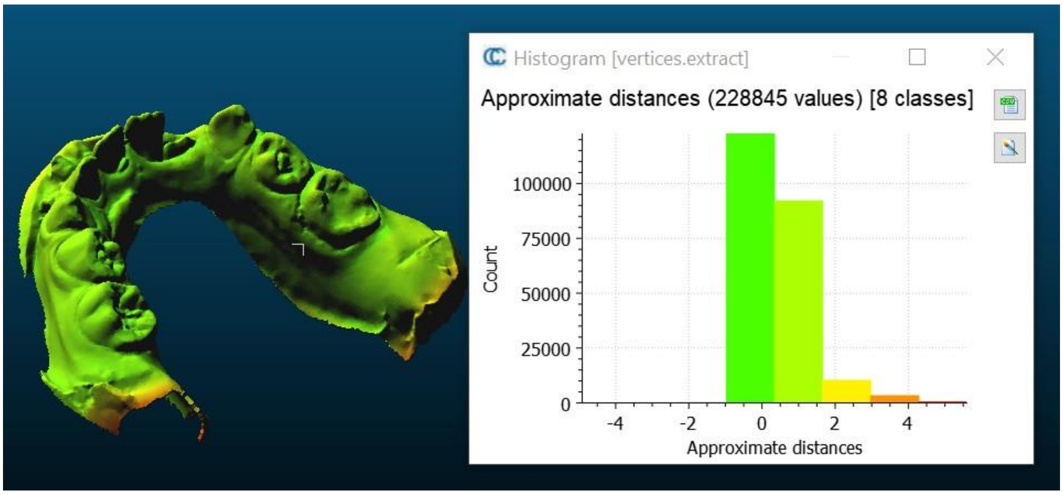

Fig 7: T03 lower jaw .

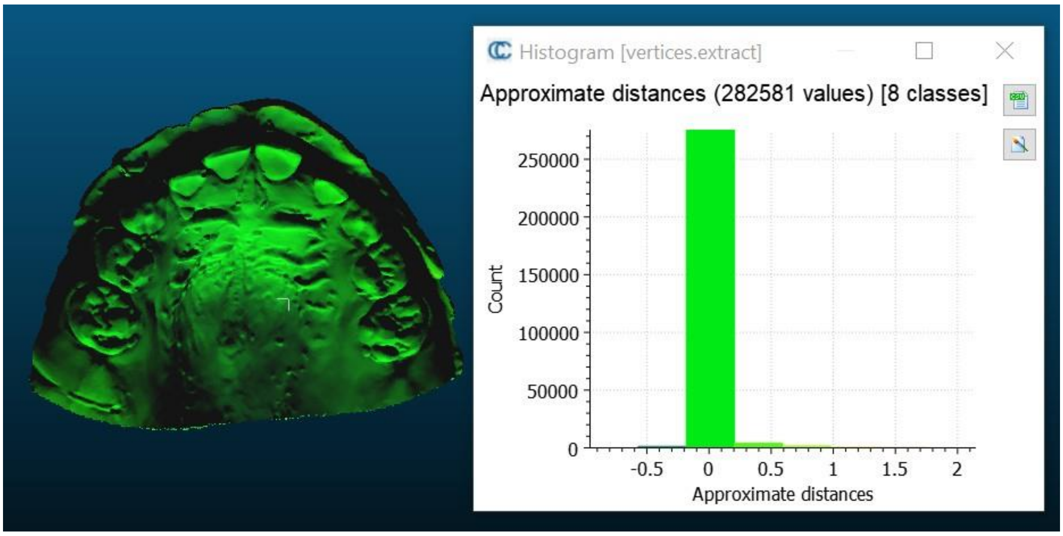

Fig 8: T03 upper jaw .

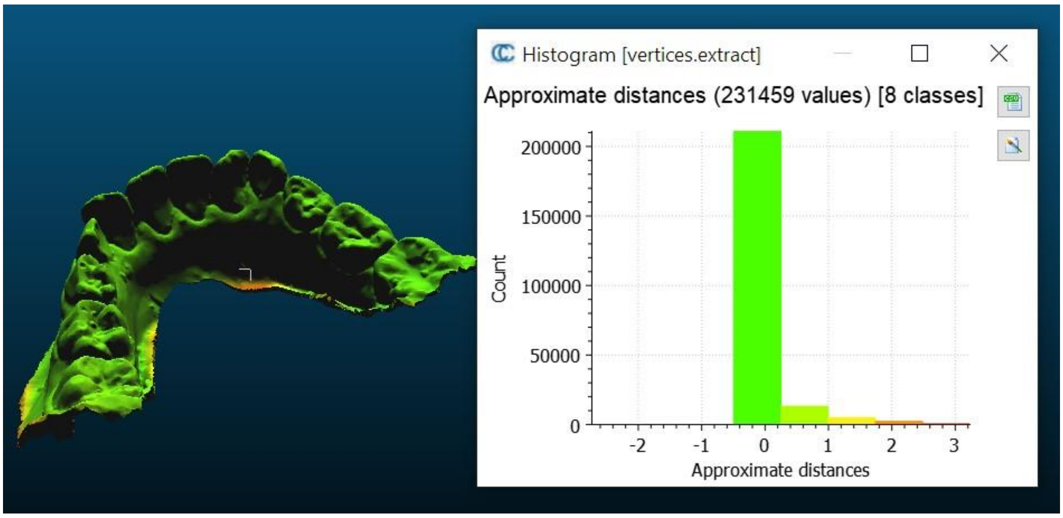

Fig 9: T04 lower jaw .

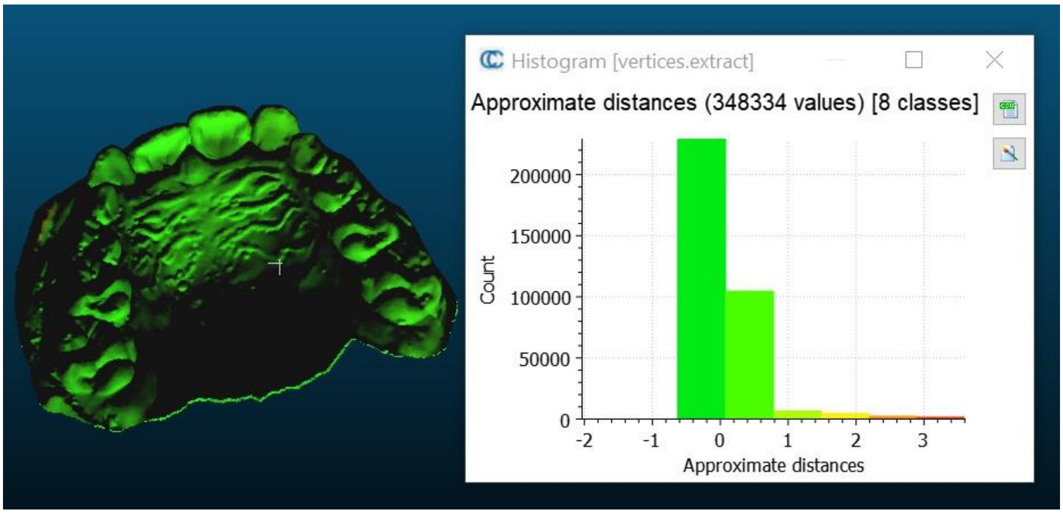

Fig 10: T04 upper jaw .

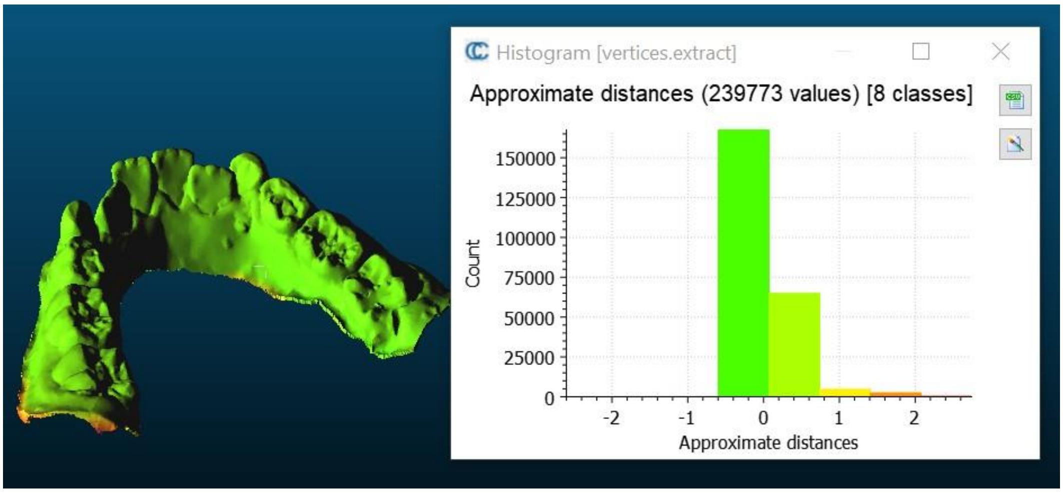

Fig 11: T05 lower jaw .

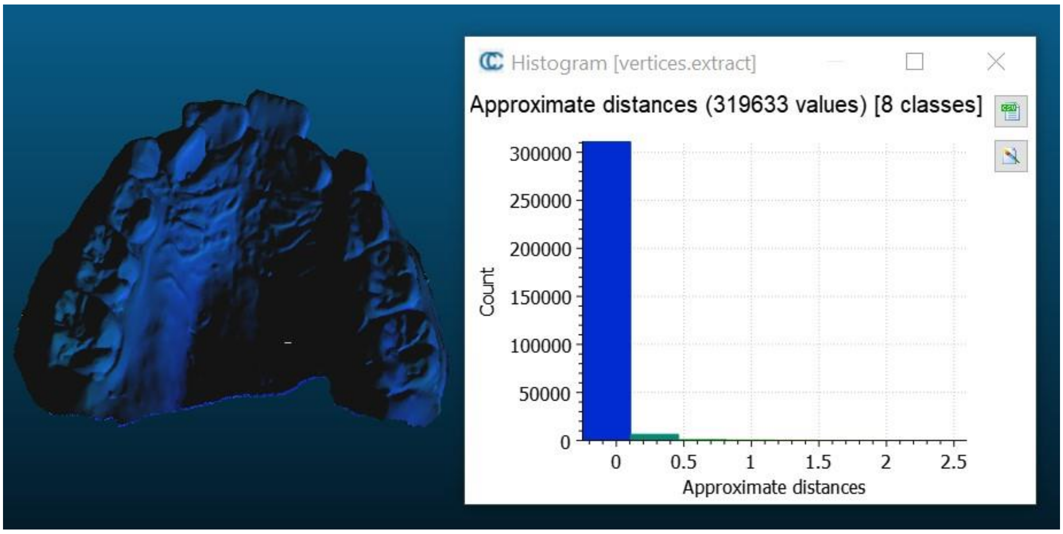

Fig 12: T05 upper jaw .

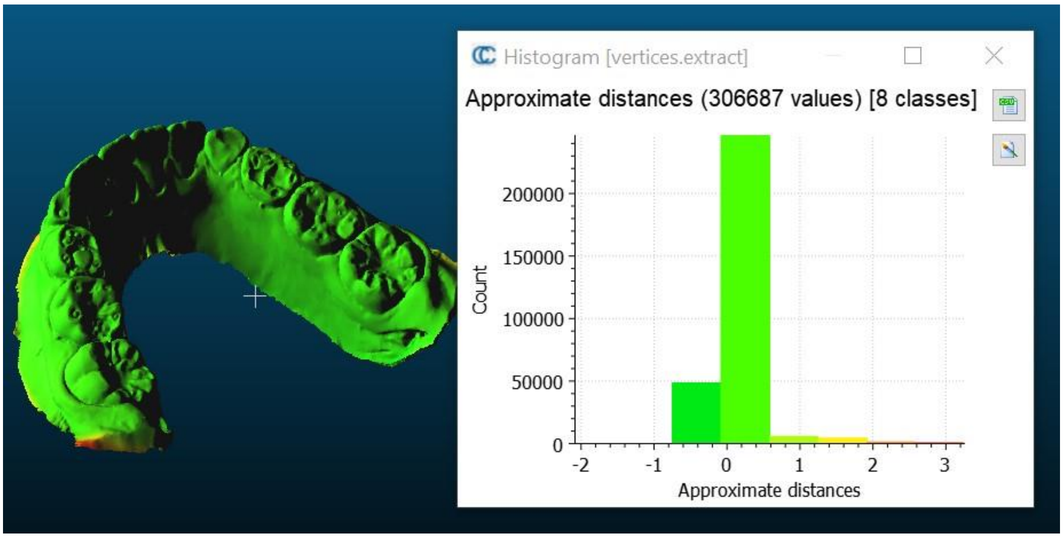

Fig 13: T06 lower jaw .

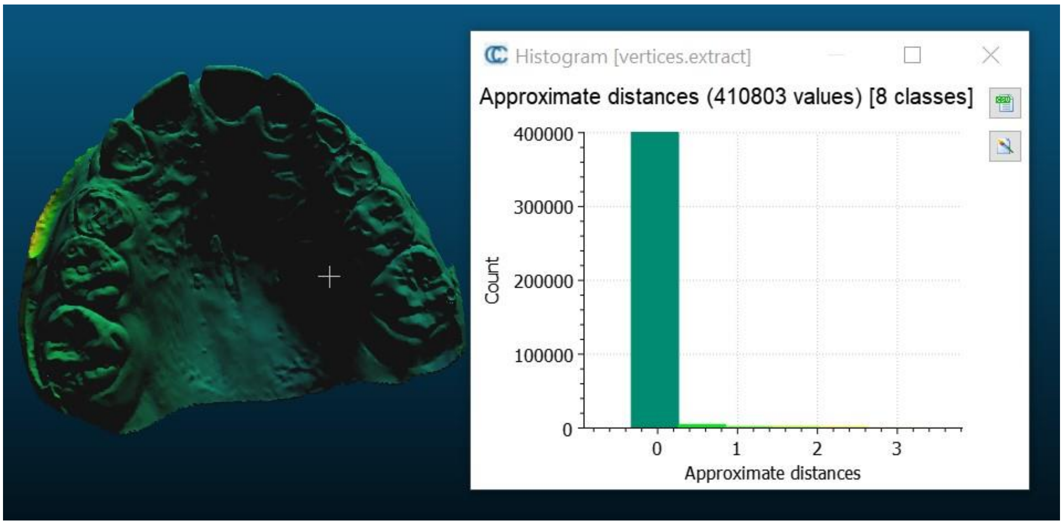

Fig 14: T06 upper jaw .

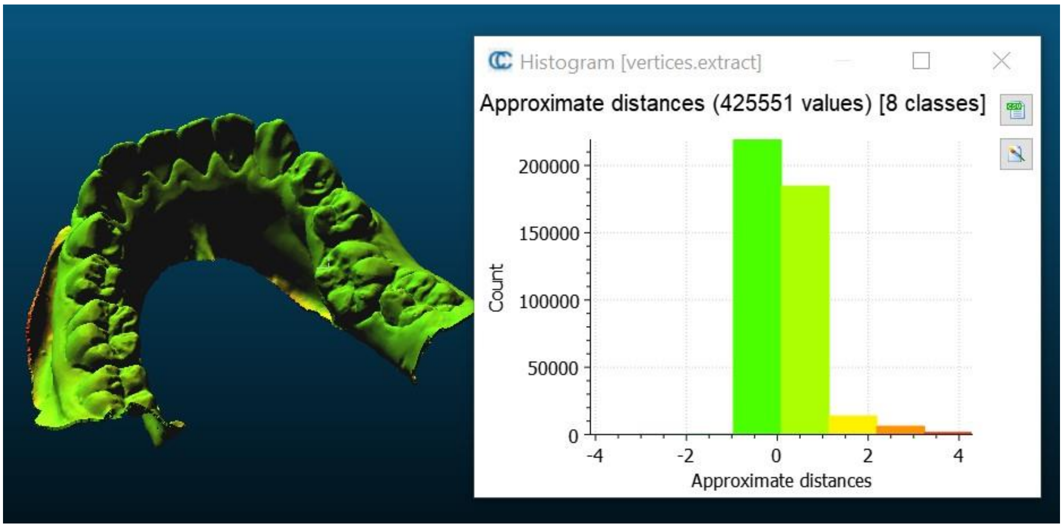

Fig 15: T07 lower jaw .

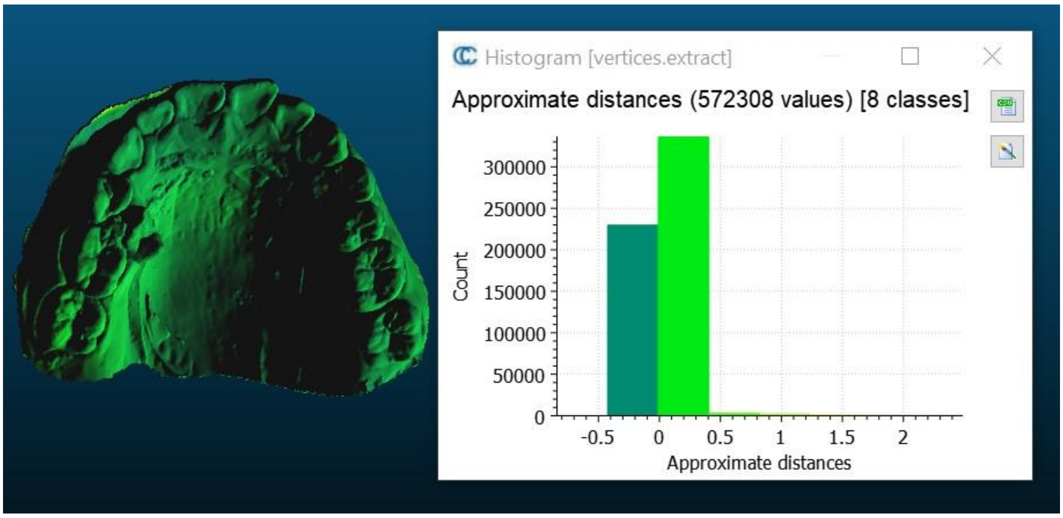

Fig 16: T07 upper jaw .

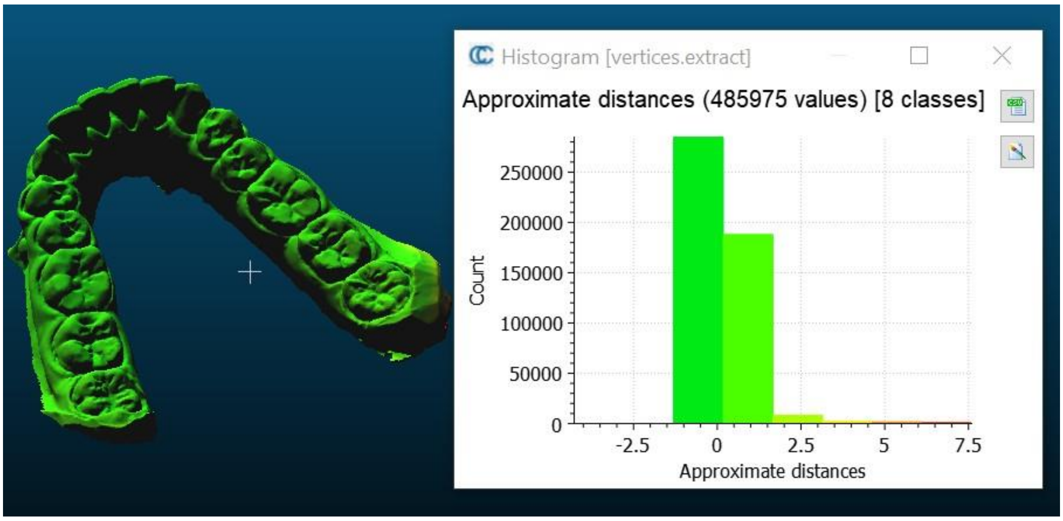

Fig 17: T08 lower jaw .

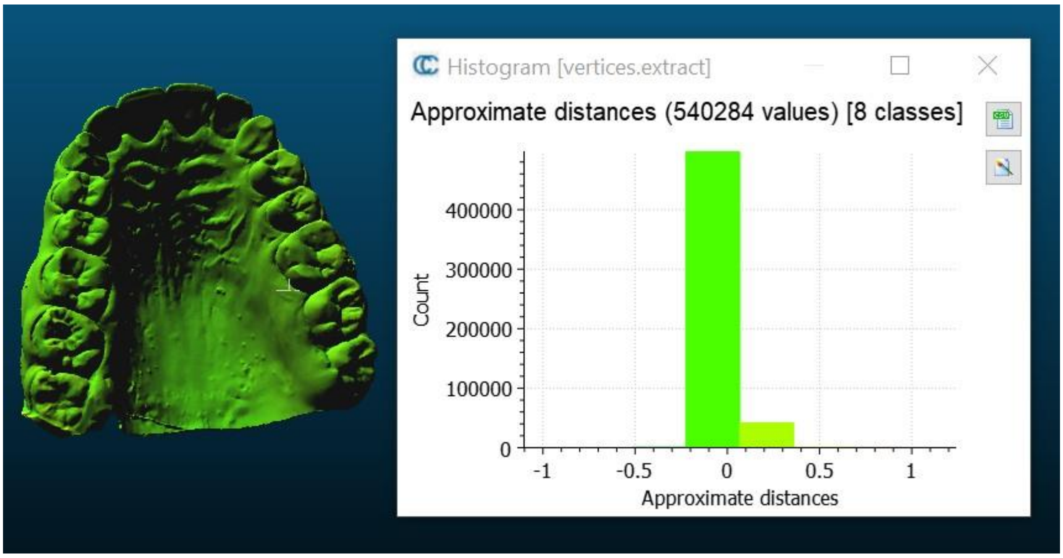

Fig 18: T08 upper jaw .

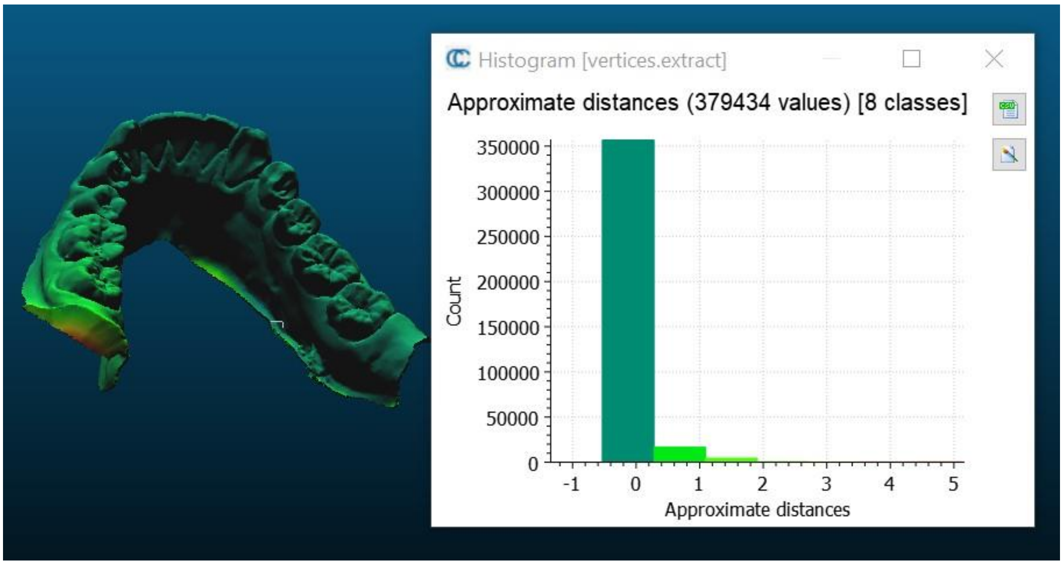

Fig 19: T09 lower jaw .

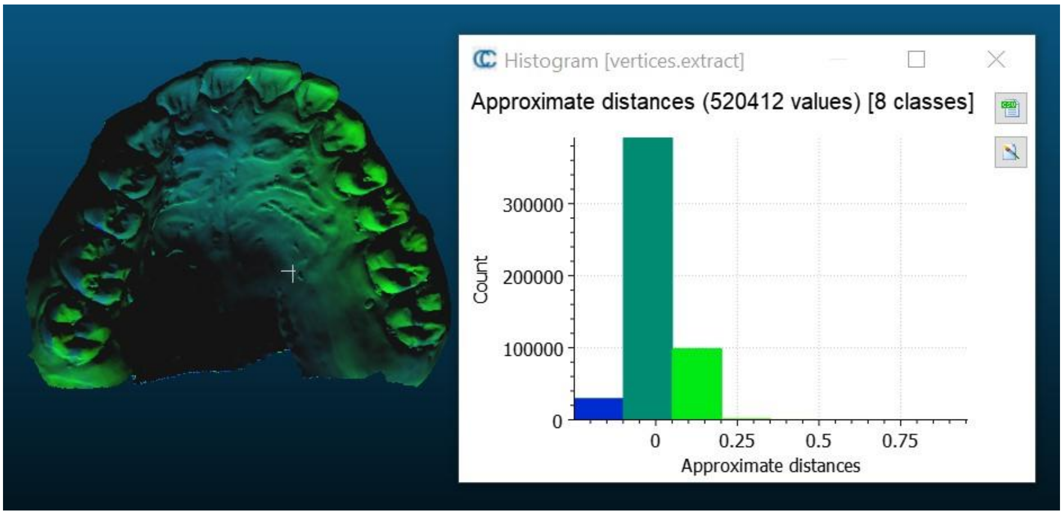

Fig 20: T09 upper jaw .

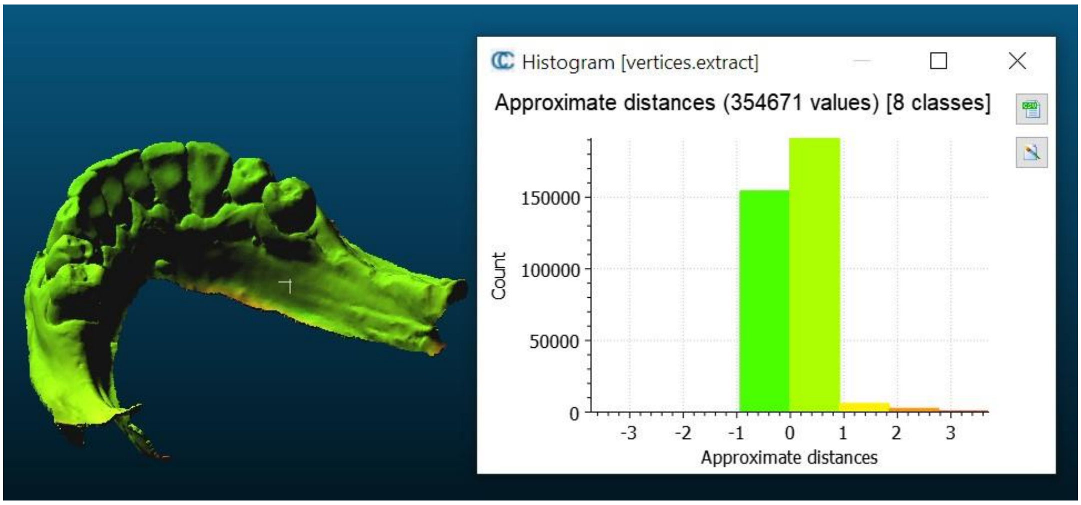

Fig 21: T10 lower jaw .

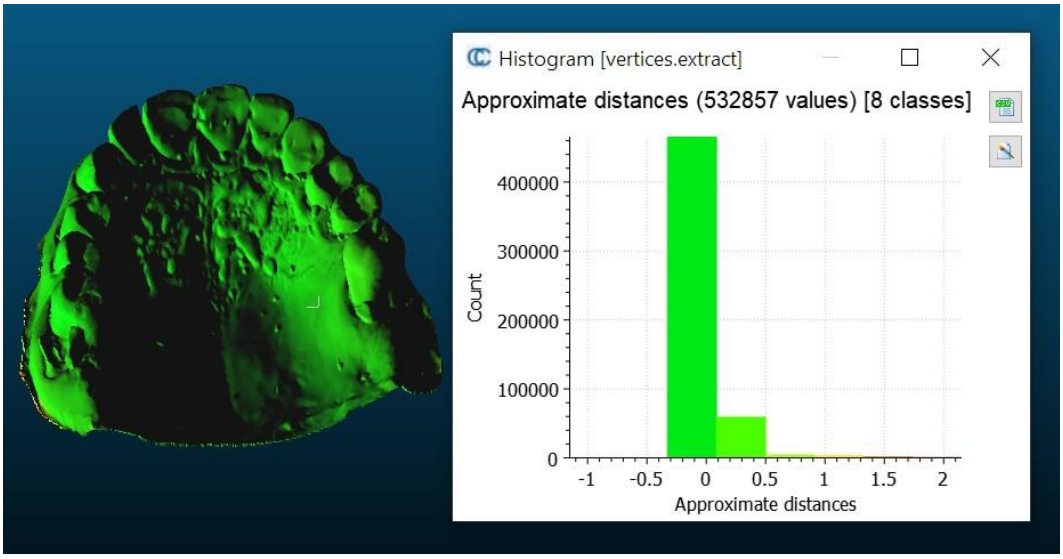

Fig 22: T10 upper jaw .

Table S6. Validation Accuracy outcomes from 3DViT

	IOS1		IOS2	
	**OP1**	**OP2**	**OP1**	**OP2**
3D printed lower				
3D printed upper				
01 Lower				
01 Upper	☑	☒	☑	☑
02 Lower	☑	☑	☑	☑
02 Upper				
03 Lower				
03 Upper	☑	☑	☑	☑
04 Lower	☑	☑	☑	☒
04 Upper				
05 Lower	☑	☑	☑	☑
05 Upper				
06 Lower				
06 Upper	☑	☑	☑	☑
07 Lower	☑	☑	☒	☑
07 Upper				
08 Lower				
08 Upper	☑	☑	☑	☑
09 Lower	☑	☑	☒	☑
09 Upper				
10 Lower				
10 Upper	☑	☑	☑	☑
	**10**	**9**	**8**	**9**

Additional References

[i] Ranftl R, Bochkovskiy A, Koltun V. Vision transformers for dense prediction. InProceedings of the IEEE/CVF International Conference on Computer Vision 2021 (pp. 12179-12188).

[ii] Liu AT, Li SW, Lee HY. Tera: Self-supervised learning of transformer encoder representation for speech. IEEE/ACM Transactions on Audio, Speech, and Language Processing. 2021 Jul 8;29:2351-66.

[iii] Gardner MW, Dorling SR. Artificial neural networks (the multilayer perceptron)—a review of applications in the atmospheric sciences. Atmospheric environment. 1998 Aug 1;32(14-15):2627-36.

[iv] Niu Z, Zhong G, Yu H. A review on the attention mechanism of deep learning. Neurocomputing. 2021 Sep 10;452:48-62.

[v] Pedamonti D. Comparison of non-linear activation functions for deep neural networks on MNIST classification task. arXiv preprint arXiv:1804.02763. 2018 Apr 8.

[vi] Nitta T. Orthogonality of decision boundaries in complex-valued neural networks. Neural computation. 2004 Jan 1;16(1):73-97.

[vii] Liu Y, Wang T, Zhang X, Sun J. Petr: Position embedding transformation for multi-view 3d object detection. InComputer Vision–ECCV 2022: 17th European Conference, Tel Aviv, Israel, October 23–27, 2022, Proceedings, Part XXVII 2022 Oct 30 (pp. 531-548). Cham: Springer Nature Switzerland.

[viii] Raghu M, Unterthiner T, Kornblith S, Zhang C, Dosovitskiy A. Do vision transformers see like convolutional neural networks?. Advances in Neural Information Processing Systems. 2021 Dec 6;34:12116-28.
